# Potential involvement of *Streptococcus mutans* possessing collagen binding protein Cnm in infective endocarditis

**DOI:** 10.1038/s41598-020-75933-6

**Published:** 2020-11-05

**Authors:** Ryota Nomura, Masatoshi Otsugu, Masakazu Hamada, Saaya Matayoshi, Noboru Teramoto, Naoki Iwashita, Shuhei Naka, Michiyo Matsumoto-Nakano, Kazuhiko Nakano

**Affiliations:** 1 Department of Pediatric Dentistry, Osaka University Graduate School of Dentistry, 1-8 Yamada-oka, Suita, Osaka 565-0871 Japan; 2 Department of Oral and Maxillofacial Surgery II, Osaka University Graduate School of Dentistry, Suita, Osaka Japan; 3 OSU Co., Ltd., Daito, Osaka Japan; 4 Department of Pharmacology, School of Veterinary Medicine, Azabu University, Sagamihara, Kanagawa Japan; 5 Department of Pediatric Dentistry, Okayama University Graduate School of Medicine, Dentistry and Pharmaceutical Sciences, Okayama, Japan

**Keywords:** Bacterial pathogenesis, Bacteriology

## Abstract

*Streptococcus mutans*, a significant contributor to dental caries, is occasionally isolated from the blood of patients with infective endocarditis. We previously showed that *S. mutans* strains expressing collagen-binding protein (Cnm) are present in the oral cavity of approximately 10–20% of humans and that they can effectively invade human umbilical vein endothelial cells (HUVECs). Here, we investigated the potential molecular mechanisms of HUVEC invasion by Cnm-positive *S. mutans*. The ability of Cnm-positive *S. mutans* to invade HUVECs was significantly increased by the presence of serum, purified type IV collagen, and fibrinogen (*p* < 0.001). Microarray analyses of HUVECs infected by Cnm-positive or -negative *S. mutans* strains identified several transcripts that were differentially upregulated during invasion, including those encoding the small G protein regulatory proteins *ARHGEF38* and *ARHGAP9*. Upregulation of these proteins occurred during invasion only in the presence of serum. Knockdown of *ARHGEF38* strongly reduced HUVEC invasion by Cnm-positive *S. mutans*. In a rat model of infective endocarditis, cardiac endothelial cell damage was more prominent following infection with a Cnm-positive strain compared with a Cnm-negative strain. These results suggest that the type IV collagen–Cnm–*ARHGEF38* pathway may play a crucial role in the pathogenesis of infective endocarditis.

## Introduction

Oral bacteria affect the pathogenesis of cardiovascular diseases^1^, including the life-threatening disease infective endocarditis (IE)^2^. Among oral streptococci, mitis group streptococci such as *Streptococcus sanguinis* and *Streptococcus mitis* are recognized as the major causative microorganisms of IE^3,4^. These bacteria are thought to invade the bloodstream following invasive dental treatments, such as tooth extraction, endodontic treatment, and periodontal surgery^5^. The bacteria subsequently adhere to abnormal heart valves to initiate IE^6^. If the bacteria are capable of invading vascular endothelial cells, the pathology of IE worsens considerably because of the cellular damage^7,8^.

Although *Streptococcus mutans* present in the oral cavity is a major causative pathogen of dental caries, it is rarely associated with the development of IE^9^. Collagen-binding protein (Cnm) has been characterized as a novel LPXTG-anchored protein of *S. mutans*^10^. Cnm-positive *S. mutans* strains are detected in the oral cavity of 10–20% of healthy subjects^11^. In a rat IE model, large bacterial masses were detected on heart valve tissue following infection with Cnm-positive *S. mutans*^12^. Interestingly, Cnm-positive *S. mutans* strains are also associated with the deterioration of intracerebral hemorrhages, which are a major complication of IE^13^.

Activation and inactivation of small G proteins involves the cytoskeletal rearrangement of human cells^14^. Various bacterial species, such as members of the genera *Staphylococcus*, *Salmonella*, *Shigella*, and *Yersinia*, invade human cells via cytoskeletal rearrangement by expressing Rho guanine nucleotide exchange factors (ARHGEFs, also known as RhoGEF), which are involved in small G protein activation, and Rho GTPase-activating proteins (ARHGAPs, also known as RhoGAP), which are involved in small G protein inactivation^15–19^. Although Cnm-positive *S. mutans* strains are capable of effectively invading human venous endothelial cells^20^, whether this occurs in an analogous manner via collagen–Cnm interactions is unknown.

In the present study, we investigated this question by analyzing the involvement of Cnm and major serum extracellular matrix (ECM) proteins in the invasion of human umbilical vein endothelial cells (HUVECs) by *S. mutans* strains, exploring genes that are differentially regulated during Cnm-dependent invasion of HUVECs, and employing a rat IE model to verify the pathophysiological relevance of our findings in vivo. The results support a potentially important role for Cnm–collagen IV interactions and the regulation of small G proteins in the development of IE following *S. mutans* infection.

## Results

### Invasion of HUVECs by Cnm-positive *S. mutans* is dependent on a serum component(s)

**Figure 1:**
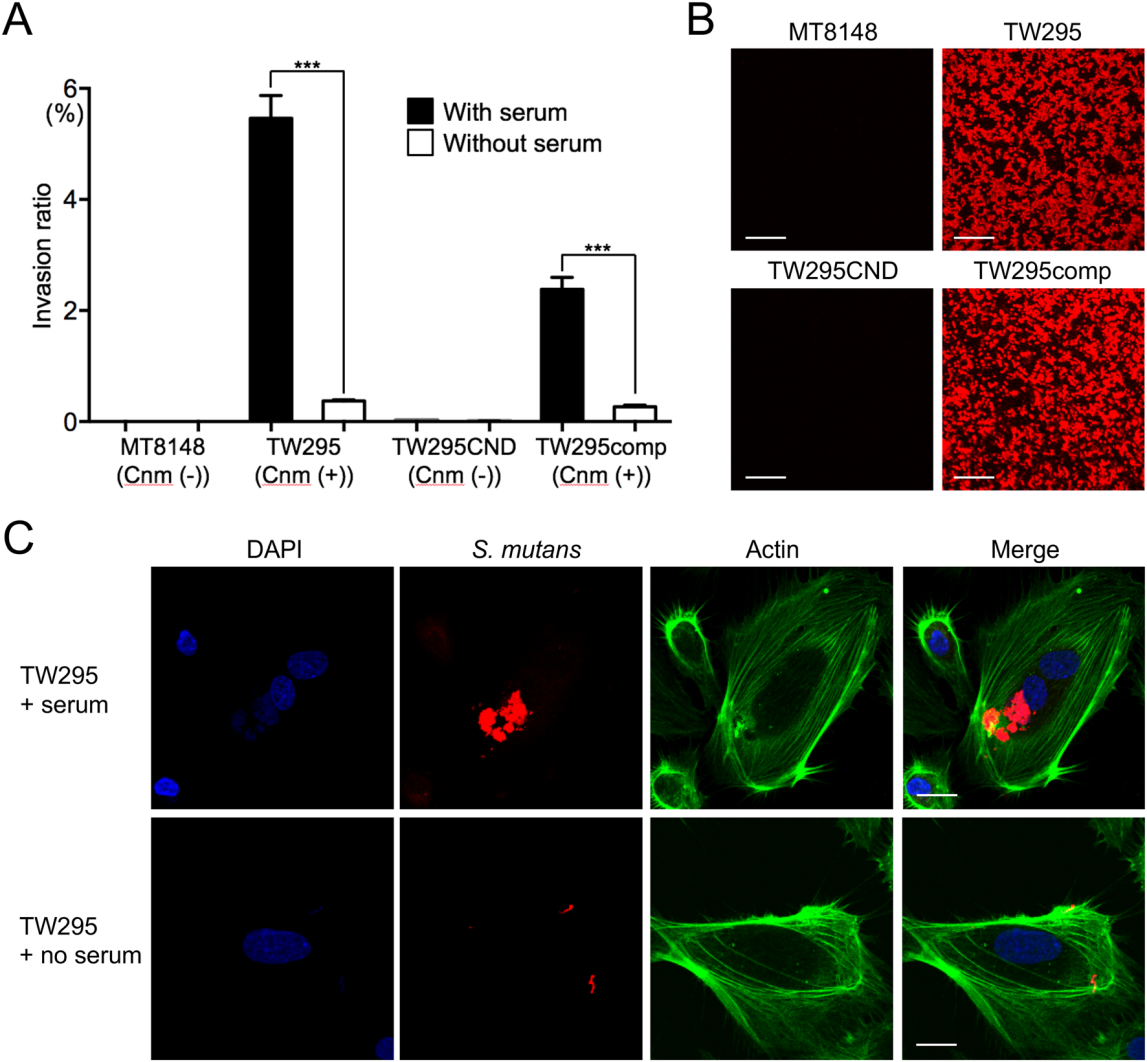
Invasion of HUVECs by *S. mutans* strains in the presence or absence of serum. (**A**) Invasion ratios of HUVECs after their incubation with the indicated *S. mutans* strains for 2 h at a multiplicity of infection of 100. Data are presented as the means ± SD of four technical replicates. ***p < 0.001 by ANOVA followed by Bonferroni’s post hoc test. (**B**) Representative confocal laser scanning microscopy images of *S. mutans*. Bacteria cells were stained red by using Alexa Fluor 555-conjugated anti-Cnm antibody. Scale bar, 10 µm. (**C**) Representative confocal laser scanning microscopy images of *S. mutans* TW295 invading HUVECs. Nuclei are stained blue (DAPI), bacteria cells are stained red (Alexa Fluor 555-conjugated anti-Cnm antibody), and actin filaments are stained green (Alexa Fluor 448-labelled phalloidin). Scale bar, 10 µm. All confocal laser scanning microscope images were taken using LSM510 (Carl Zeiss, Oberkochem, Germany).

We first examined the ability of Cnm-positive and Cnm-negative *S. mutans* strains to invade HUVECs in the presence or absence of serum by using a HUVEC invasion assay. MT8148, a Cnm-negative clinical strain^21^, was unable to invade the cells regardless of the presence of serum (Fig. 1A). In contrast, TW295, a Cnm-positive *S. mutans* strain isolated from a subject with bacteremia after tooth extraction^22^, displayed strong invasion of HUVECs in the presence of serum and significantly weaker invasion in the absence of serum (*p* < 0.001). Notably, invasion was not observed following the incubation of HUVECs with TW295CND, a Cnm-defective isogenic mutant strain^23^, in either the presence or absence of serum (Fig. 1A), while TW295comp, a Cnm-complemented mutant strain^12^, displayed recovered cell invasion ability in the presence of serum (Fig. 1A). Thus, *S. mutans* invades HUVECs in a serum- and Cnm-dependent manner. Confocal laser scanning microscopy observations revealed that a Cnm-specific antibody specifically stained the Cnm-positive *S. mutans* strains (Fig. 1B) and confirmed that TW295 exhibited high levels of invasion only in the presence of serum (Fig. 1C). Additional experiments using an anti-*S. mutans* antibody that reacted with MT8148 confirmed that the Cnm-negative *S. mutans* strain MT8148 did not invade vascular endothelial cells (Supplementary Fig. 1A,B).

### Cnm-positive *S. mutans* binds to collagen and fibrinogen

**Figure 2:**
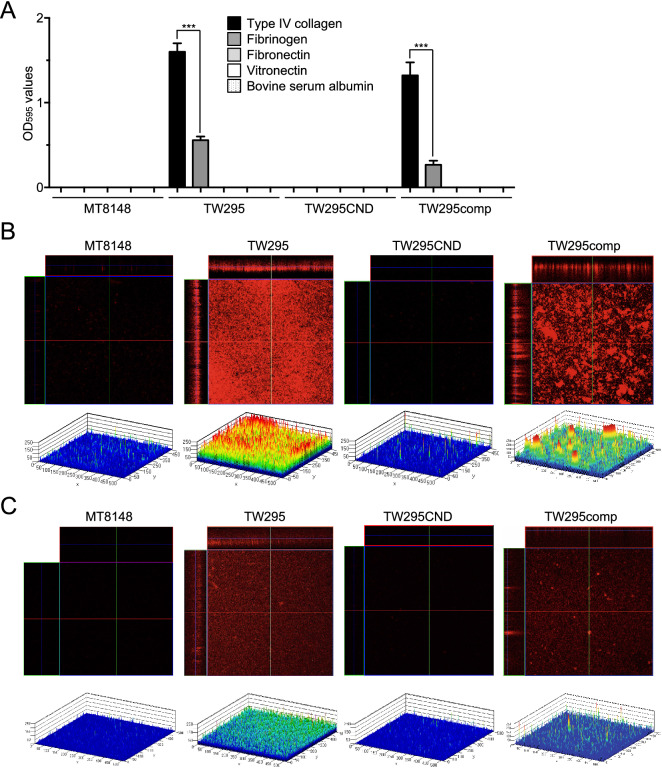
ECM binding of *S. mutans* strains. (**A**) Binding of the indicated *S*. *mutans* strains to type IV collagen, fibrinogen, fibronectin, or vitronectin. Data are presented as the means ± SD of three technical replicates. ***p < 0.001 by ANOVA followed by Bonferroni’s post hoc test. (**B**, **C**) Confocal laser scanning microscopy images (upper panels) and schematics (lower panels) of *S. mutans* binding to type IV collagen (**B**) or fibrinogen (**C**). Color coding of the biofilm thickness: 0–50 µm, blue; 50–100 µm, light blue; 100–150 µm, green; 150–200 µm, yellow; and 200–250 µm, red. All confocal laser scanning microscope images and schematics were made using LSM510.

We next sought to determine which major serum ECM component(s) was crucial for *S. mutans* invasion of HUVECs by examining bacterial adhesion to purified collagen, fibrinogen, fibronectin, and vitronectin in vitro. While MT8148 did not adhere to any of the tested ECM proteins, TW295 bound to both type IV collagen and fibrinogen, with type IV collagen being the preferred substrate (*p* < 0.001, Fig. 2A). This binding activity was dependent on Cnm expression, as illustrated by the lack of binding to either protein by TW295CND and the recovered binding demonstrated by TW295comp. We also evaluated *S. mutans* binding to type IV collagen and fibrinogen by confocal laser scanning microscopy of hexidium iodide-stained bacteria. This analysis confirmed the adhesion assay results, revealing that TW295 and TW295comp both formed thicker biofilms on type IV collagen than on fibrinogen, whereas no binding was observed by MT8148 or TW295CND (Fig. 2B,C).

### Type IV collagen promotes invasion of HUVECs by *S. mutans*

**Figure 3:**
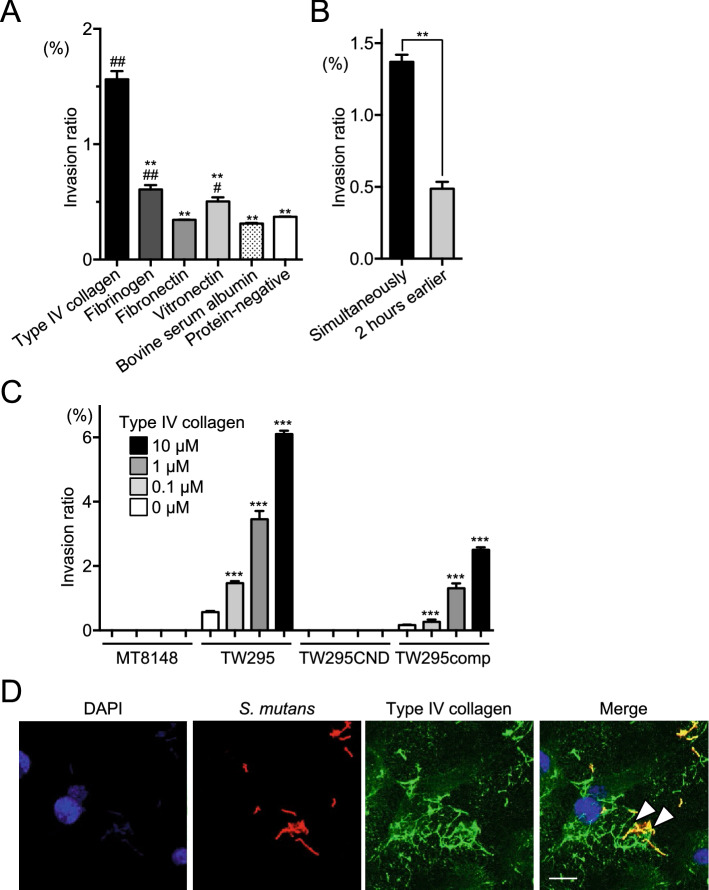
Invasion of HUVECs by *S. mutans* in the presence of purified ECM proteins. (**A**) Invasion ratios of HUVECs after their incubation with *S. mutans* strain TW295 for 2 h at a multiplicity of infection of 100 in the presence of type IV collagen (140 ng/ml), fibrinogen (2 mg/ml), fibronectin (0.2 mg/ml), vitronectin (500 µg/ml), or bovine serum albumin (1 mg/ml). Data are presented as the means ± SD of three technical replicates. *p < 0.05, **p < 0.01 versus TW295 and ^#^*p* < 0.05, ^##^*p* < 0.01 versus the protein-negative control, using ANOVA followed by Bonferroni’s post hoc test. (**B**) Ability of TW295 to invade HUVECs when type IV collagen and bacteria were added simultaneously or when type IV collagen was added 2 h before the bacterial infection. Data are presented as the means ± SD of three technical replicates. **p < 0.01 using a Student’s *t*-test. (**C**) HUVEC invasion ratio (as described for **A**) of the indicated strains in the presence of various concentrations of type IV collagen. Data are presented as the means ± SD of four technical replicates. ***p < 0.001 versus no collagen using ANOVA followed by Bonferroni’s post hoc test. (**D**) Representative confocal laser scanning microscopy images of *S. mutans* TW295 invading HUVECs. Bacteria are stained red (Alexa Fluor 555-conjugated anti-Cnm antibody), type IV collagen is stained green (Alexa Fluor 488), and nuclei are stained blue (DAPI). Arrowheads indicate areas were type IV collagen and bacteria colocalize. Scale bar, 10 µm. All confocal laser scanning microscope images were taken using LSM510.

To determine whether the ECM components are necessary for HUVEC invasion by *S. mutans*, we examined cell invasion by TW295 in serum-free medium individually supplemented with one of four ECM components at concentrations corresponding to those detected in healthy human blood [type IV collagen (140 ng/ml), fibrinogen (2 mg/ml), fibronectin (0.2 mg/ml), and vitronectin (500 µg/ml)^24–27^]. As expected, the level of HUVEC invasion by TW295 was higher in the presence of type IV collagen as compared with in the presence of the other tested proteins (Fig. 3A). When type IV collagen was added 2 h before bacterial infection, cell invasion by TW295 was inhibited, and the invasion ratio was significantly lower than that when type IV collagen and TW295 were added simultaneously (*p* < 0.01) (Fig. 3B). Additionally, the degree of invasion was positively associated with the type IV collagen concentration (Fig. 3C). Cnm expression was required for HUVEC invasion, as illustrated by the inability of *S. mutans* strains MT8148 and TW295CND to invade HUVECs, even in the presence of high type IV collagen concentrations. Confocal laser scanning microscopy of fluorescently labeled HUVECs and TW295 confirmed the presence of internalized bacteria in endothelial cells (Fig. 3D).

### Identification of differentially expressed genes upon Cnm-dependent invasion of HUVECs by *S. mutans*

**Figure 4:**
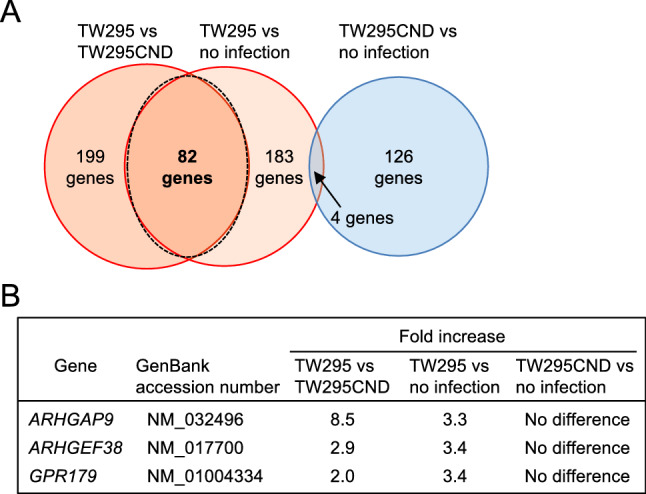
DNA microarray analysis of HUVEC genes involved in invasion by *S. mutans*. (**A**) Venn diagram showing the selection of genes by their differential expression (> 2.0-fold difference) in the indicated infected cells. A total of 82 genes were significantly upregulated both in TW295-infected HUVECs compared with TW295CND-infected HUVECs and in TW295-infected HUVECs compared with uninfected HUVECs but were not differentially expressed in TW295CND-infected HUVECs compared with uninfected HUVECs. (**B**) Three of the 82 differentially expressed genes were identified as regulators of small G proteins. Microarray data was analyzed using Agilent Feature Extraction (Agilent Technologies, Santa Clara, CA, USA).

To identify key regulators of Cnm-dependent *S. mutans* internalization, we performed a comparative DNA microarray analysis on three pairs of HUVECs: TW295CND-infected *vs* uninfected cells (to identify genes related to *S. mutans* invasion independently of Cnm); TW295-infected *vs* uninfected cells (to identify genes related to Cnm-positive *S. mutans* invasion); and TW295-infected *vs* TW295CND-infected cells (to identify genes specifically related to the involvement of Cnm in HUVEC invasion). Lists of genes with altered expression between each of these groups are shown in Supplementary Tables 1, 2 and 3. Among these variable genes, a total of 82 genes were significantly upregulated in HUVECs infected by TW295 in a Cnm-dependent manner (Fig. 4A, Supplementary Table 4). As expected, none of the 82 genes was overexpressed in TW295CND-infected HUVEC as compared with uninfected cells.

Various bacterial species invade human cells via cytoskeletal rearrangement by expressing ARHGEFs, which are involved in small G protein activation, and ARHGAPs, which are involved in small G protein inactivation^15–19^. To determine if genes related to the activation or inactivation of small G proteins might play a role here, we examined the list of differentially expressed genes for ARHGEFs and ARHGAPs, and we identified three: Rho GTPase-activating protein 9 (*ARHGAP9*), Rho guanine nucleotide exchange factor 38 (*ARHGEF38*), and G protein-coupled receptor 179 (*GPR179*) (Fig. 4B).

### Transcripts of small G protein regulators are involved in HUVEC invasion by *S. mutans* invasion

**Figure 5:**
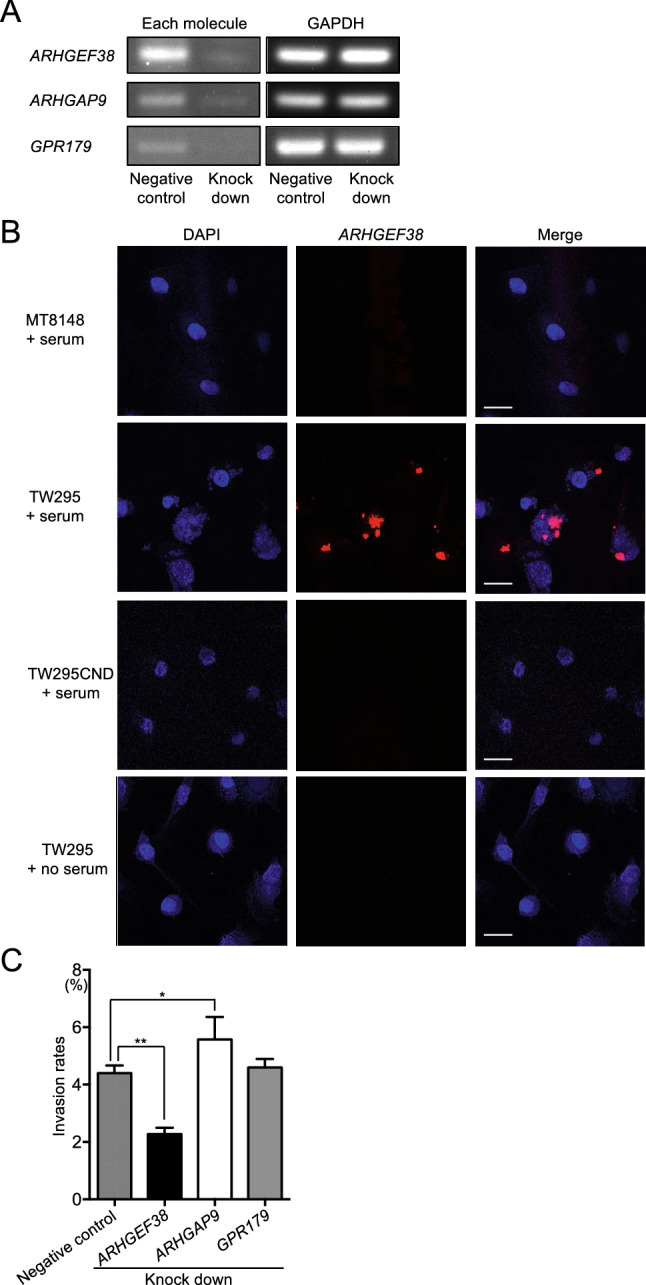
Effect of silencing genes involved in HUVEC invasion by *S. mutans*. (**A**) RT-PCR analysis of HUVECs transfected with siRNAs targeting *ARHGAP9*, *ARHGEF38*, or *GPR179*. GAPDH mRNA expression was analyzed as an internal control. (**B**) Representative confocal laser scanning microscopy images of HUVECs invaded by *S. mutans* in the presence or absence of serum. Nuclei are stained blue (DAPI) and each transcript (*ARHGAP9*, *ARHGEF38*, and *GPR179*) is stained red (Alexa Fluor 555). Arrowheads indicate the expression of each transcript. (**C**) HUVECs were transfected with *ARHGEF38*, *ARHGAP9*, *GPR179,* or control siRNAs. Invasion ratios of these HUVECs after their incubation with *S. mutans* strain TW295 for 2 h at a multiplicity of infection of 100. Data are presented as the means ± SD of four technical replicates. *p < 0.05, **p < 0.01 using ANOVA followed by Bonferroni’s post hoc test. Scale bar, 10 µm. All confocal laser scanning microscope images were taken using LSM510.

To confirm the involvement of the three small G protein regulatory genes in the internalization of Cnm-positive *S. mutans* into HUVECs, we knocked down the expression of each gene in HUVECs via transfecting the cells with specific small interfering RNA (siRNA). Effective silencing of *ARHGAP9*, *ARHGEF38*, and *GPR179* expression was confirmed by RT-PCR analyses of the transfected cells (Fig. 5A; full-length is shown in Supplementary Fig. 2). As shown in Fig. 5B (*ARHGEF38*) and Supplementary Fig. 3 (*ARHGAP9* and *GPR179*), all three genes were strongly upregulated during TW295 infection of HUVECs in the presence of serum, confirming their potential involvement in invasion. However, only the *ARHGEF38* knockdown resulted in a significant inhibition of HUVEC invasion by TW295 *S. mutans* (*p* < 0.01, Fig. 5C). Interestingly, the *ARHGAP9* knockdown in HUVECs significantly increased the invasion ratio as compared with cells transfected with the negative control siRNA (*p* < 0.05), whereas the *GPR179* knockdown had no significant effect on invasion (Fig. 5C).

### Evaluations of vascular endothelial cell damage and histopathological findings in a rat IE model

**Figure 6:**
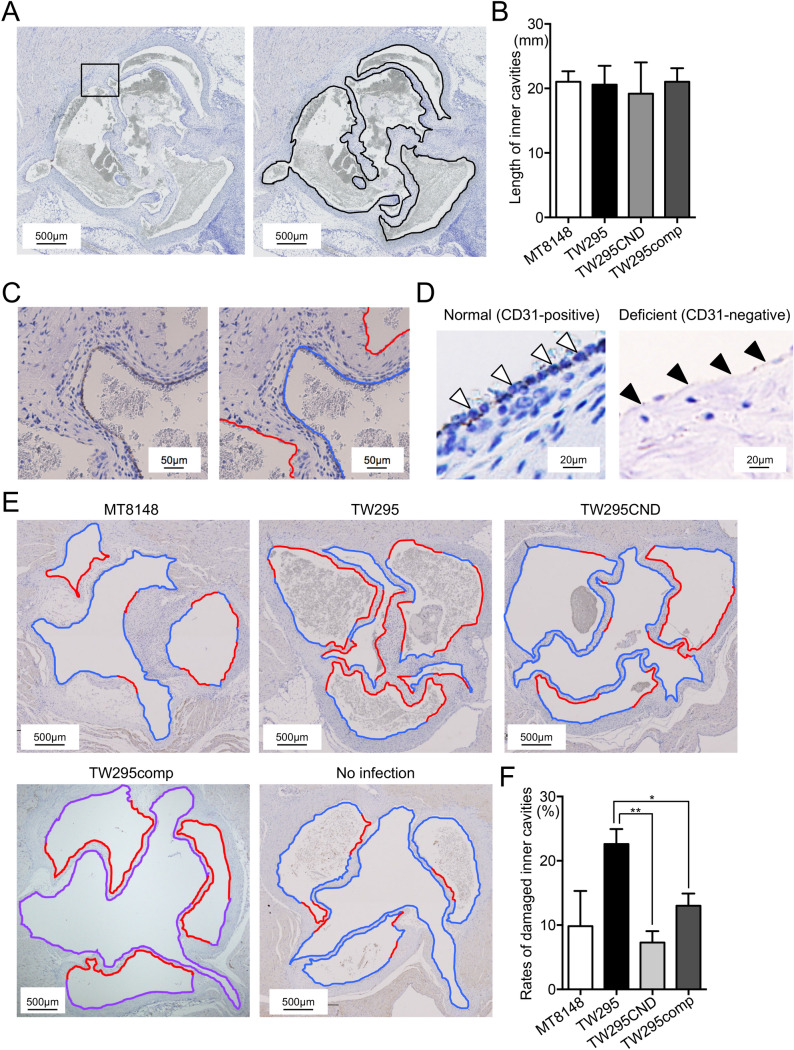
Evaluation of vascular endothelial cell damage in a rat IE model. (**A**) Representative IHC images of anti-CD31-stained sections of extirpated heart valves from rats infected with *S. mutans* strain TW295. Black lines indicate the total length of the endocardium. (**B**) Total length of the endocardium of extirpated hearts from rats infected with the indicated *S. mutans* strain. Data are presented as the mean ± SD of six biological replicates per strain. (**C**) Magnified images of the box outlined in (**A**). Right panel shows endothelial cell damage. Blue and red lines indicate non-damaged (CD31-positive) and damaged (CD31-negative) endocardium, respectively. (**D**) Representative magnified images of normal (CD31-positive; arrowheads in left panel) and deficient (CD31-negative; arrowheads in right panel) endocardium. (**E**) Representative images of vascular endothelial cell damage in hearts from rats infected with the indicated *S. mutans* strains. Blue and red lines indicate non-damaged (CD31-positive) and damaged (CD31-negative) endocardium, respectively. (**F**) Rates of damaged endocardium following infection by each *S. mutans* strain. Data are presented as the mean ± SD of six biological replicates per strain. *p < 0.05, **p < 0.01 using ANOVA followed by Bonferroni’s post hoc test. The lengths of total, CD31-positive, and CD31-negative endocardium in the heart valves were measured using WinROOF Vol 5.0 software (Mitsuya Co., Ltd., Tokyo, Japan).

Finally, we verified our findings in vivo by evaluating the effects of *S. mutans* infection on vascular endothelial cells using a rat IE model. An aortic valve injury was induced in rats by using a sterile polyethylene catheter, which was inserted through the right carotid artery and then immediately removed after causing the heart valve injury. The rats were then infected with one strain of *S. mutans* via the jugular vein. All rats survived for 7 days after the bacterial infection, after which they were euthanized, and their hearts were extirpated. To visualize healthy (CD31-positive) and damaged (CD31-negative) endothelial cells, sections of the rat hearts were stained for CD31 expression by using immunohistochemistry (IHC). The lengths of the damaged and healthy endocardium were then measured and compared using imaging software. Representative IHC images displaying the total lengths of the endocardium are shown in Fig. 6A. No significant differences were detected in the total endocardium length between hearts from rats infected with MT8148, TW295, or TW295CND (Fig. 6B). Representative images of vascular endothelial cell damage in the heart sections are shown in Fig. 6C–F. An evaluation of the proportions of undamaged and damaged endothelial cells between rat groups revealed a significantly greater percentage of damaged endocardium in the hearts of rats infected with TW295 compared with those infected with TW295CND (*p* < 0.01, Fig. 6F). Collectively, these data underscore the potential pathological relevance of Cnm-dependent invasion of endothelial cells by *S. mutans* in the development of IE.

In addition to evaluating the vascular endothelial cell damage, we assessed the histopathology by performing hematoxylin–eosin staining of tissue sections (Supplementary Fig. 4), then scoring them with the following scale: 0 (none), 1 (mild), 2 (moderate), and 3 (severe). Among the six evaluation points by which the histopathological findings were assessed, five in the TW295-infected group and two in the TW295comp-infected group showed significantly higher values compared with those for the non-infected group (*p* < 0.05) (Table 1). In contrast, only one evaluation point had a significantly higher score for the MT8148 strain-infected group as compared with the non-infected group (*p* < 0.05), and none of the evaluation point scores were significantly different between the TW295CND-infected group and non-infected group.

**Table 1:** Histopathological evaluation of extirpated heart tissues from IE model rats.

Histopathological finding	MT8148 (n = 6)	TW295 (n = 6)	TW295CND (n = 6)	TW295comp (n = 6)	No infection (n = 6)
Infiltration of inflammatory cells	1.17 ± 0.28**	1.83 ± 0.40**	0.17 ± 0.17^#^	2.17 ± 0.40**	0.17 ± 0.17^##^
Hypertrophy of the endocardium	0.75 ± 0.11^##^	1.83 ± 0.31*	0.33 ± 0.21^#^	0.50 ± 0.34^##^	0.33 ± 0.33^#^
Hypertrophy of the annulus	1.08 ± 0.42	1.33 ± 0.49	0.00 ± 0.00^#^	0.17 ± 0.17	0.33 ± 0.33
Acceleration of fibrosis	1.25 ± 0.34	1.50 ± 0.41*	0.00 ± 0.00^#^	1.67 ± 0.33**	0.17 ± 0.17^#^
Fibrin-like deposition	1.00 ± 0.34	1.58 ± 0.27*	0.08 ± 0.08^##^	1.00 ± 0.52	0.33 ± 0.33^#^
Bacterial mass	0.08 ± 0.08^#^	1.33 ± 0.42*	0.00 ± 0.00^#^	0.17 ± 0.17^#^	0.00 ± 0.00^#^

Values in each column indicate mean ± standard error. There were significant differences between no infection (*P < 0.05, **P < 0.01) and TW295 groups (^#^*P* < 0.05, ^##^*P* < 0.01).

## Discussion

The invasion of *S. mutans* into vascular endothelial cells is considered an exacerbating factor in the development of IE^28^. Although we previously showed that Cnm-positive, but not Cnm-negative, *S. mutans* strains exhibited high levels of invasion into vascular endothelial cells^20^, the invasion mechanism was unknown. Because Cnm-positive *S. mutans* form aggregates when mixed with serum^12^, we suspected that a serum component may be crucial to the internalization of Cnm-positive *S. mutans* strains into vascular endothelial cells.

Bacterial adhesion to ECM proteins is important for host cell invasion^29^. Fibronectin-binding proteins are the major adhesins involved in the invasion of vascular endothelial cells by *S. aureus*, a major pathogen of acute IE^30^. In the present study, however, we found that type IV collagen was the major serum ECM protein involved in HUVEC invasion by Cnm-positive *S. mutans*. Thus, Cnm-positive *S. mutans* and fibronectin-binding proteins-positive *S. aureus* use different ECM proteins to invade vascular endothelial cells.

Most streptococcal species are isolated from the oral cavity or nasopharynx^31^, and adherence is a major virulence factor for streptococcal species. Collagen-binding proteins have been reported to be present in some streptococcal species, including *Streptococcus pyogenes*, *Streptococcus pneumoniae*, *Streptococcus equi*, and *Streptococcus gallolyticus*^31–34^. *S. mutans* Cnm has no homology with the collagen-binding proteins of *S. pyogenes* and *S. pneumoniae*, which are commensal bacteria in humans that are sometimes associated with severe systemic diseases. In contrast, *S. mutans* Cnm has 40–50% homology with the collagen-binding proteins of *S. equi* and *S. gallolyticus*^10,35^, which are infrequently detected in humans. *S. gallolyticus*, which is rarely involved in the onset of IE, can adhere to and invade vascular endothelial cells^36^; however, the mechanism by which these bacteria invade into vascular endothelial cells has not been elucidated. The bacterial species that encode a collagen-binding protein with homology to *S. mutans* Cnm, including *S. gallolyticus*, may have an invasion mechanism similar to that of Cnm-positive *S. mutans*.

In our previous study, Cnm was found to be an important factor for the invasion of *S. mutans* into vascular endothelial cells^37^. Cnm is co-transcribed with PgfS, which is located immediately downstream and encodes a putative glycosyltransferase; notably, the deletion of PgfS reduces the cell’s ability to invade vascular endothelial cells^38^. Therefore, a lack of PgfS expression in the Cnm-complemented mutant strain may be one reason why the pathogenicity of the complemented mutant strain was not completely restored to wildtype levels. Furthermore, in a two-component system of *S. mutans*, CovR is known as a positive regulator of Cnm, while VicRKX is a negative regulator of Cnm^39^, and both a VicRKX overexpression strain and a CovR inactivation mutant strain have a reduced ability to invade vascular endothelial cells. These proteins that regulate Cnm expression may affect the mechanism by which Cnm-positive *S. mutans* invade into vascular endothelial cells.

Bacterial invasion of host cells is accompanied by an increased expression of host genes involved in the regulation of small G proteins, leading to actin cytoskeleton remodeling^15,40^. Here, we performed a microarray analysis to identify genes related to Cnm-dependent HUVEC invasion by *S. mutans,* and we used RNA interference and invasion assays to verify the microarray results. These analyses revealed a crucial role for *ARHGEF38* and *ARHGAP9* in endothelial cell invasion by Cnm-positive *S. mutans*. Although our understanding of the detailed functions of the ARHGEF family is incomplete^41^, these proteins are guanine nucleotide exchange factors that promote the activation of Rho family G proteins by inducing GDP dissociation, thereby enabling GTP binding^14^. In turn, the GDP–GTP exchange promotes a cytoskeletal rearrangement that facilitates bacterial internalization, as demonstrated with *Yersinia* species, which are Gram-negative bacteria associated with digestive diseases^18,19^. In the present study, we found that an *ARHGEF38* knockdown significantly inhibited HUVEC invasion by TW295, but this inhibition was incomplete, suggesting that other pathways also support the invasion of vascular endothelial cells by Cnm-positive *S. mutans.*

Intriguingly, we found that an *ARHGAP9* knockdown in Cnm-positive *S. mutans* significantly increased their ability to invade HUVECs. *ARHGAP9* is a member of the ARHGAP family of GTPase-activating proteins, which promote the inactivation of Rho family G proteins through the hydrolysis of bound GTP^42^. A previous study showed that *ARHGAP9* overexpression in human leukemia cells significantly reduced their level of adhesion to type IV collagen^43^. Thus, our results suggest that *ARHGAP9* normally prevents the invasion of Cnm-positive *S. mutans* by inhibiting type IV collagen binding.

Several animal models of IE have been developed, including dog, pig, rabbit, and rat models; of these, the rat model is the most commonly used^44,45^. We recently examined the virulence of *S. mutans* strains using the rat IE model^12^, and histopathological analyses revealed increased bacterial masses in abnormal heart valves. In the present study, we evaluated endothelial cell damage in *S. mutans*-infected rats by staining heart sections with anti-CD31 antibody, which allowed us to quantify the damage to endothelial cells.

Recent studies have reported correlations between Cnm-positive *S. mutans* infection and intracerebral hemorrhage^13,46^, which is a major complication of IE that occurs in approximately 10% of cases^47^. We previously demonstrated in a mouse model that Cnm-positive *S. mutans* strains caused deterioration of intracerebral hemorrhages that involved photochemical damage to the endothelium of the middle cerebral artery^13^. Cnm-positive *S. mutans* were observed around the damaged blood vessels, suggesting that they may contribute to the hemorrhage. This possibility should be analyzed in more detail in future studies.

**Figure 7:**
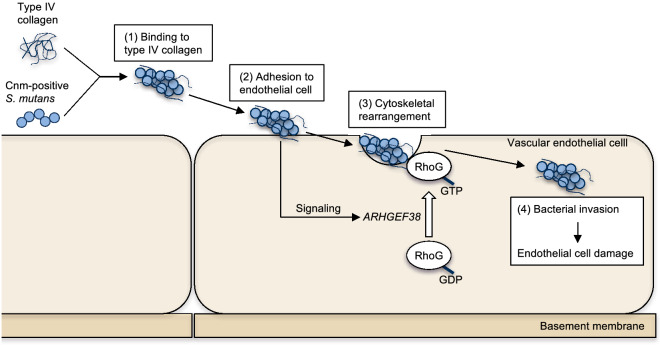
Proposed model of the interaction between Cnm-positive *S. mutans* and vascular endothelial cells. Cnm-positive *S. mutans* strains adhere to type IV collagen in the serum, and (2) then adhere to vascular endothelial cell surfaces. (3) The binding of bacteria activates *ARHGEF38* in the endothelial cells, leading to GDP–GTP exchange and the activation of Rho family G proteins, cytoskeletal rearrangement, and bacterial internalization. (4) Invasion by Cnm-positive *S. mutans* induces vascular endothelial cell damage.

A schematic of the mechanism by which Cnm-positive *S. mutans* may invade vascular endothelial cells during IE is shown in Fig. 7. We speculate that Cnm-positive *S. mutans* in the blood adhere to type IV collagen in serum and attach to endothelial cell surfaces, inducing an upregulation of *ARHGEF38* expression in the endothelial cells. In turn, *ARHGEF38* induces the activation of Rho family G proteins, leading to cytoskeletal rearrangement, which facilitates bacterial internalization. The invasion of Cnm-positive *S. mutans* results in vascular endothelial cell damage that subsequently contributes to many cardiovascular and cerebrovascular diseases.

In summary, we showed here that *S. mutans* Cnm, its interaction with type IV collagen in serum, and the upregulation of *ARHGEF38* in endothelial cells are all important for the invasion of Cnm-positive *S. mutans* into vascular endothelial cells. These events may therefore contribute to the deterioration of IE associated with Cnm-positive *S. mutans* infection.

## Methods

### Ethics statement

All rats were treated humanely, in accordance with the guidelines of the National Institutes of Health and the AERI-BBRI Animal Care and Use Committee. Animal experiments were approved by the Institutional Animal Care and Use Committee of Osaka University Graduate School of Dentistry (approval number 24-019-0).

### *S. mutans* strains

MT8148 (serotype *c*) is a Cnm-negative strain isolated from the oral cavity of a healthy child^21^, TW295 (serotype *k*) is a Cnm-positive strain isolated from an individual with bacteremia after tooth extraction, TW295CND is the Cnm-defective isogenic mutant strain of TW295^22,23^, and TW295comp is the Cnm-complemented mutant strain of TW295^12^. All strains were confirmed to be *S. mutans* on the basis of their biochemical properties and the observation of rough colony morphology on Mitis-Salivarius agar plates (Difco Laboratories, Detroit, MI, USA) containing bacitracin (0.2 U/ml; Sigma-Aldrich, St. Louis, MO, USA) and 15% (wt/vol) sucrose (MSB agar), as well as the results of a sequence analysis targeting 16S rRNA, with the primers 8UA (5′-AGAGTTTGATCCTGGCTCAG-3′) and 1540R (5′-AAGGAGGTGATCCAGCC-3′), as described previously^48^. For routine growth, all strains were cultured overnight in brain heart infusion (BHI) broth (Difco Laboratories). BHI broth containing erythromycin (10 µg/ml) was used to culture TW295CND, and BHI broth containing erythromycin (10 µg/ml) and spectinomycin (1 mg/ml) was used to culture TW295comp.

The presence or absence of *cnm* was determined by applying the PCR method, using primers cnm-1F (5′-GACAAAGAAATGAAAGATGT-3′) and cnm-1R (5′-GCAAAGACTCTTGTCCCTGC-3′), which can amplify the entire length of the *cnm* gene^11^. The putative amino acid sequence of Cnm from *S. mutans* strain Z1 (GenBank accession number AB102689), found in a previous study^10^, had a region with homology with the collagen-binding domain of the *Staphylococcus aureus* collagen-binding protein Cna with 54.8% identity, and this region of Cnm had collagen-binding ability^10^. The Cnm-positive *S. mutans* strain TW295 used in the present study also had the Cnm collagen-binding domain, which was previously registered in GenBank (accession number AB469913).

The Cnm-defective isogenic mutant strain TW295CND was generated in our previous study^23^. Briefly, the erythromycin resistance gene was inserted into the gene encoding the collagen-binding domain, located in the central part of *cnm*. A plasmid containing a complex of the erythromycin resistance gene and the collagen-binding gene was then introduced into a Cnm-positive *S. mutans* strain using a homologous recombination method. The appropriate insertional inactivation of the isogenic mutant was confirmed by Southern hybridization^23^ and western blotting^49^. The absence of collagen-binding ability in the isogenic mutant strain was confirmed by a collagen-binding assay^23^, in which the strain was attached to the collagen-coated plate using a previously described method^11^.

The Cnm-complemented strain TW295comp was generated in a previous study^12^. Briefly, the entire length of *cnm* was amplified and cloned into the shuttle vector pDL278. The plasmid containing *cnm* was then added to bacterial broth of TW295CND, which was cultured in Todd–Hewitt medium supplemented with 10% heat-inactivated horse serum (Invitrogen, Carlsbad, CA, USA). After being incubated for 2 h at 37 °C, the cells were plated on Mitis-salivarius agar containing spectinomycin (1 mg/ml) and erythromycin (10 µg/ml), followed by anaerobic incubation at 37 °C for 48 h, to select for the complemented mutant TW295comp strain. The successful generation of TW295comp was confirmed in our previous study by performing a PCR analysis for the presence of pDL278 in the *cnm* gene and collagen-binding assays^12^.

### HUVEC invasion assay

HUVECs were purchased from Lonza (Walkersville, MD, USA), and bacterial invasion was evaluated as previously described^37,50^, with some modifications. In brief, 1 × 10^5^ HUVECs were seeded in 24-well plates (Corning Inc., Corning, NY, USA) and washed three times with phosphate-buffered saline (PBS). Antibiotic-free EBM-2 medium (Lonza) and *S. mutans* (1 × 10^7^ colony-forming units [CFU]) were added to the HUVECs. When purified ECM proteins were present, serum was omitted, and type IV collagen (Cellmatrix Type IV; Nitta Gelatin, Osaka, Japan), fibrinogen, fibronectin, and vitronectin (all from human plasma; Sigma-Aldrich) were added to the medium at final concentrations equivalent to those found in healthy human blood^24–27^. The cells were incubated for 2 h, after which the supernatant was removed, and the infected cells were washed three times with PBS. Medium containing penicillin (50 µg/ml) and gentamycin (300 µg/ml) was added to the wells, and the plates were then incubated for an additional 3 h. The preliminary experiments for this study revealed that this mixture of antibiotics could kill 1 × 10^9^ CFU of each *S. mutans* strain used in the present study within 3 h. Additionally, all invasion assays included controls (no HUVECs), in which antibiotics were used to verify the killing of each *S. mutans* strain. The cells were lysed by the addition of sterile distilled water, and dilutions of the resulting lysates were plated onto MSB plates and cultured at 37 °C for 2 days. The invasion ratio was calculated as the ratio of recovered bacteria to the initial inoculum. Data are expressed as the mean ± standard deviation (SD) of triplicate experiments.

### Fluorescence microscopy of *S. mutans* and HUVECs

The observation of *S. mutans* strains using confocal laser scanning microscopy was performed as follows. Each *S. mutans* strain, adjusted to a concentration of 1 × 10^9^ CFU using PBS, was streaked on cover glasses, and fixed with 3% paraformaldehyde (Wako Pure Chemical Industries, Osaka, Japan) for 10 min. The *S. mutans* strains were then stained with rabbit anti-Cnm serum^35^ or anti-*S. mutans* antibody (whole cell as immunogen; Abcam, Cambridge, MA, USA) followed by detection with Alexa Fluor 555-conjugated goat anti-rabbit IgG (Molecular Probes, Life Technologies, Eugene, OR, USA).

Bacterial invasion of HUVECs was confirmed using confocal laser scanning microscopy as described previously^20^. In brief, invaded cells were washed three times with PBS, fixed with 3% paraformaldehyde for 10 min, washed with PBS, and incubated with primary antibodies for 1 h. After being washed, the cells were incubated with secondary antibodies for 30 min. If necessary, the cells were permeabilized by the addition of 0.4% Triton X-100 for 5 min. The following antibodies were used: rabbit anti-Cnm serum, detected with Alexa Fluor 555-conjugated goat anti-rabbit IgG; anti-ARHGEF38, anti-ARHGAP9, or anti-GPR179 (all Funakoshi Corporation, Tokyo, Japan), detected with Alexa Fluor 555-conjugated goat anti-rabbit IgG (Molecular Probes); and anti-type IV collagen (Abcam), detected with Alexa Fluor 488-conjugated goat anti-rabbit IgG (Molecular Probes). The cytoskeleton was visualized by staining actin filaments with Alexa Fluor 488-conjugated phalloidin (Molecular Probes). Nuclei were stained with 4′,6-diamidino-2-phenylindole dihydrochloride (DAPI; Wako Pure Chemical Industries). All antibodies were diluted 1:500 in 0.5% bovine serum albumin (BSA) in PBS. Cells were examined using a confocal laser scanning microscope (LSM510; Carl Zeiss, Oberkochem, Germany) with a 63 × oil immersion objective.

### Bacterial ECM-binding assay

The ECM-binding properties of *S. mutans* were evaluated as described by Nomura et al*.*^11^, using a protocol originally developed by Waterhouse and Russell^51^ with some modifications. Type IV collagen, fibrinogen, fibronectin, and vitronectin (the major ECM components of serum) were added to the wells of 96-well tissue culture plates (Becton Dickinson, Franklin Lakes, NJ, USA) and incubated overnight at 4 °C. The plates were then washed three times with PBS, blocked for 1.5 h with 5% BSA in PBS at 37 °C, and washed again with PBS containing 0.01% Tween 20. *S. mutans* were collected by centrifugation from overnight cultures in BHI broth, diluted with PBS, and added to the coated wells (concentration: 2 × 10^9^ CFU/well). After a 3-h incubation at 37 °C, non-adherent cells were removed by washing the plates with PBS three times, and the adherent cells were fixed with 25% formaldehyde (200 µl) at room temperature for 30 min. The cells were then washed three times with PBS and stained by the addition of 0.05% crystal violet (Wako Pure Chemical Industries) in water (200 µl) for 1 min. After the plates were washed again, the dye was dissolved by the addition of 7% acetic acid (200 µl), and the absorbance at 595 nm was recorded. Data are expressed as the mean ± SD of triplicate experiments.

### Microscopy of ECM-bound cells

*Streptococcus mutans* binding to type IV collagen and fibrinogen was also assessed using confocal laser scanning microscopy as described previously^52^ with some modifications. Type IV collagen or fibrinogen were added to chambered coverglass wells (CultureWell; Grace Bio Labs, Bend, OR, USA) and incubated overnight at 4 °C. The coated wells were washed three times with PBS, blocked for 1.5 h with 5% BSA in PBS at 37 °C, and washed again with PBS containing 0.01% Tween 20. *S. mutans* cells were collected, stained with hexidium iodide (Molecular Probes), and added to the coated wells (2 × 10^9^ CFU/well) in PBS. The cells were cultured anaerobically at 37 °C for 18 h in the dark. Non-attached *S. mutans* cells were removed by washing with PBS, and the adherent cells were observed using a confocal laser scanning microscope (LSM510; Carl Zeiss, Oberkochem, Germany) with a 63 × oil immersion objective.

### DNA microarray assays

Genes involved in HUVEC invasion by Cnm-positive *S. mutans* were identified by DNA microarray assays as described previously^53^. Amino-allyl-amplified RNA was obtained from total RNA using an Amino-allyl MessageAmp aRNA kit (Ambion, Austin, TX, USA), and samples were labeled with Cy3 or Cy5 using FluoroLink Cy3 or Cy5 monofunctional Dye 5-Packs (Amersham Bioscience, London, UK). Samples were applied to a gene chip system (Sigma Genosis Japan, Sapporo, Japan), gene expression was detected by Cy3 and Cy5 fluorescence intensity, and signal transduction-related genes whose transcripts were considered to be important for bacterial internalization were identified. Genes significantly differentially expressed between (1) uninfected and TW295-infected HUVECs and (2) TW295- and TW295CND-infected HUVECs were identified, and overlapping genes were noted (Fig. 4A). Genes significantly differentially expressed between TW295CND-infected and uninfected HUVECs were excluded to remove genes involved in infection by Cnm-negative *S. mutans* strains. Genes were considered significantly differentially expressed if the change in expression was > 2.0-fold.

### Silencing of gene expression

siRNA transfection of HUVECs was performed as described previously^54^ with some modifications. siRNAs targeting *ARHGEF38*, *ARHGAP9*, *GPR179*, or a negative control sequence (siRNA AllStars Negative Control siRNA) were purchased from Qiagen (Düsseldorf, Germany). HUVECs (1 × 10^5^ cells/well) were seeded in 24-well plates and incubated for 24 h. The cell culture medium was then discarded and replaced with serum- and antibiotic-free medium containing siRNAs (final concentration: 40 nM) and Lipofectamine 2000 (Thermo Fisher Scientific, Waltham, MA, USA).

### RT-PCR assay

Silencing of gene expression was assessed by RT-PCR at 24 h post-transfection. Total RNA was prepared from the HUVECs after the cells had been transfected with siRNA using a Pure Link RNA Mini Kit (Thermo Fisher Scientific) in accordance with the manufacturer’s instructions. A High Capacity RNA-to-cDNA Kit (Thermo Fisher Scientific) was then used to amplify cDNA synthesized from the mRNA. The primers used to detect the transcription of each gene are shown in Supplementary Table 5. PCR assays were performed in 20-µl reaction mixtures containing TaKaRa Ex *Taq* polymerase (TAKARA BIO, Inc., Otsu, Shiga, Japan) in accordance with the manufacturer’s protocols. PCR amplification was performed under the following conditions: initial denaturation at 95 °C for 4 min, followed by 30 cycles at 95 °C for 30 s, 55 °C for 30 s, and 72 °C for 30 s, with a final extension step at 72 °C for 7 min. The PCR products were fractionated using a 1.5% (w/v) agarose gel containing Tris-acetate-EDTA buffer, stained with ethidium bromide (0.5 µg/ml), and visualized under UV illumination.

### Rat model of IE

A previously described rat IE model was used^55^ with some modifications. Briefly, 30 Sprague-Dawley male rats (weighing 250–300 g) were anesthetized via injection of xylazine and midazolam, and a sterile polyethylene catheter with a guide wire was inserted through the right carotid artery to injure the aortic valve. The catheter was immediately removed after the heart valve injury. A bacterial suspension (10^8^ cells/rat in PBS) was then intravenously administered through the jugular vein. All rats survived for 7 days after the bacterial infection. At this time, the rats were euthanized by anesthesia overdose, and their hearts were extirpated, sectioned transversely, and stained with anti-CD31 antibody (Abcam) to visualize vascular endothelial cells. The lengths of total, CD31-positive, and CD31-negative endocardium in the heart valves were measured using WinROOF Vol 5.0 software (Mitsuya Co., Ltd., Tokyo, Japan). The rate of endocardium damage was calculated as: ([length of CD31-negative endocardium in each heart valve of infected rats]/[length of total endocardium in each heart valve of infected rats]) − ([average length of CD31-negative endocardium in heart valves of uninfected rats]/[average length of total endocardium in heart valves of uninfected rats]) × 100. Histopathological features were evaluated by performing hematoxylin–eosin staining of tissue sections and scoring them as 0 (none), 1 (mild), 2 (moderate), or 3 (severe), as previously described^55^. All scoring evaluations were performed in a double-blinded fashion by a pathologist (Sept. Sapie Co., Ltd., Tokyo, Japan).

### Statistical analysis

Statistical analyses were performed using GraphPad Prism 6 (GraphPad Software, La Jolla, CA, USA). Intergroup differences were compared using an analysis of variance (ANOVA) with Bonferroni’s post hoc test. Comparisons between two groups were performed using a Student’s *t *test. A *p *value of < 0.05 was considered to indicate a significant difference.

## Supplementary information

Supplementary Information.

## Data Availability

All data generated or analyzed during the described study are included in this published article (and its “Supplementary Information” files).
