# Supplementary material for: Potential involvement of Streptococcus mutans possessing collagen binding protein Cnm in infective endocarditis
Source: Sci Rep. 2020 Nov 5;10:19118. doi: 10.1038/s41598-020-75933-6 (PMC7645802; doi:10.1038/s41598-020-75933-6)
Supplement: Supplementary file 1 — Supplementary Information. [file 41598_2020_75933_MOESM1_ESM.pdf]

Potential involvement of *Streptococcus mutans* possessing collagen binding protein Cnm in infective endocarditis

Ryota Nomura, Masatoshi Otsugu, Masakazu Hamada, Saaya Matayoshi, Noboru Teramoto, Naoki Iwashita, Shuhei Naka, Michiyo Matsumoto-Nakano, Kazuhiko Nakano

Supplementary Table 1. Changes in gene expression of TW295CND with respect to TW295.

Sequence ID	Sequence Code	Primary Sequenc	Sequence Description	Accession #	Log(Ratio)	Fold Change	P-value
2313823	A_33_P3754922	RNU11	BP873537 Sugano cDNA library, embryonal kidney Homo sapiens ‹	BP873537	2	100	3.30E-23
2313224	A_33_P3403270		coiled-coil domain containing 33 [Source:HGNC Symbol;Acc:2655:	CN265765	2	100	6.45E-23
1145348	A_23_P82099	NOX3	Homo sapiens NADPH oxidase 3 (NOX3), mRNA [NM_015718]	NM_015718	2	100	2.90E-23
886683	A_32_P54274	DRD5	Homo sapiens dopamine receptor D5 (DRD5), mRNA [NM_000798]	NM_000798	2	100	2.68E-23
1155509	A_24_P492083		AF150244 Human mRNA from cd34+ stem cells Homo sapiens cD	AF150244	1.95368	89.88314	1.46E-22
879364	A_23_P155057	CYTH4	Homo sapiens cytohesin 4 (CYTH4), mRNA [NM_013385]	NM_013385	1.9024	79.87289	2.20E-22
2333454	A_19_P00329766		lincRNA:chrX:115401722-115413847 forward strand		1.85721	71.98043	1.07E-22
2332992	A_33_P3276386		Unknown		1.84041	69.24859	1.17E-22
2325685	A_33_P3214670	HK2	Homo sapiens hexokinase 2 (HK2), mRNA [NM_000189]	NM_000189	1.82895	67.44449	2.71E-22
885170	A_23_P355394	STK32A	Homo sapiens serine/threonine kinase 32A (STK32A), transcript v	NM_145001	1.80305	63.5404	6.87E-22
1139895	A_23_P304304	ARSF	Homo sapiens arylsulfatase F (ARSF), mRNA [NM_004042]	NM_004042	1.73614	54.46828	1.34E-22
2313161	A_33_P3331150		Human IG rearranged H-chain mRNA V-region, partial cds. [L0383	L03830	1.72954	53.64606	2.08E-21
1137544	A_23_P425681	CCK	Homo sapiens cholecystokinin (CCK), transcript variant 1, mRNA		NM_000729	1.72347	52.90116	1.72E-22
2332858	A_33_P3261695	LOC647310	Homo sapiens testis expressed gene 22 (LOC647310), mRNA [NM	NM_001195082	1.6761	47.4346	1.81E-22
2313619	A_19_P00805987		lincRNA:chr18:53398227-53474402 reverse strand		1.64171	43.82396	3.54E-22
2317053	A_33_P3236122		Unknown		1.63463	43.11478	1.50E-20
2313034	A_33_P3303982	UBTFL1	Homo sapiens upstream binding transcription factor, RNA polymer	NM_001143975	1.63372	43.02512	1.49E-20
2316927	A_33_P3221578		PREDICTED: Homo sapiens FLJ45872 protein (FLJ45872), miscRI	XR_114214	1.60106	39.90776	3.48E-20
2328342	A_19_P00807753		lincRNA:chr5:67086094-67113044 reverse strand		1.59765	39.59581	2.06E-20
2325165	A_33_P3358943	GRM2	Homo sapiens glutamate receptor, metabotropic 2 (GRM2), transc	NM_000839	1.58812	38.73644	6.52E-22
2332835	A_19_P00324742		lincRNA:chr5:54162493-54208143 forward strand		1.58462	38.4257	7.66E-22
1147323	A_23_P316472	DNHD1	Homo sapiens dynein heavy chain domain 1 (DNHD1), transcript v.	NM_144666	1.57709	37.7648	2.72E-22
1140900	A_23_P259049	SLC45A2	Homo sapiens solute carrier family 45, member 2 (SLC45A2), tran:	NM_016180	1.57612	37.68072	5.67E-22
2317054	A_19_P00813078		lincRNA:chr4:74537611-74598511 forward strand		1.57167	37.29701	4.47E-22
2332860	A_19_P00804713		lincRNA:chrX:39980256-39991281 forward strand		1.54869	35.37449	1.41E-19
2312901	A_19_P00323817		lincRNA:chr11:129133890-129145940 forward strand		1.54452	35.03653	1.74E-19
2330100	A_33_P3507270	EFCAB5	Homo sapiens EF-hand calcium binding domain 5 (EFCAB5), trans	NM_198529	1.53012	33.89347	2.99E-19
1160252	A_24_P169507	MUC17	Homo sapiens mucin 17, cell surface associated (MUC17), mRNA		NM_001040105	1.52064	33.16204	3.64E-19
2332602	A_33_P3404257	VN1R103P	Homo sapiens cDNA FLJ43558 fis, clone PROST2018607. [AK125	AK125546	1.51067	32.40945	4.80E-21
2332859	A_33_P3294277	CYP4F3	Homo sapiens cytochrome P450, family 4, subfamily F, polypeptide	NM_000896	1.506	32.06258	4.33E-21
1139286	A_32_P202182	SPDYE8P	Homo sapiens speedy homolog E8 (Xenopus laevis), pseudogene (:	NR_003664	1.49854	31.51696	1.82E-21
1161067	A_23_P148641	TTTY12	Homo sapiens testis-specific transcript, Y-linked 12 (non-protein	NR_001551	1.49183	31.03371	5.37E-20
2328230	A_33_P3348395		Uncharacterized protein ENSP00000372125 [Source:UniProtKB/Swiss-Prot;Acc:A		1.44062	27.58173	5.63E-21
869222	A_24_P390495	CX3CL1	Homo sapiens chemokine (C-X3-C motif) ligand 1 (CX3CL1), mRN	NM_002996	1.39741	24.96954	4.13E-22
2313089	A_33_P3311543		Unknown		1.39519	24.84209	1.55E-20
2316774	A_19_P00807075		lincRNA:chr1:224624052-224647602 reverse strand		1.3921	24.66581	5.83E-17
2313136	A_19_P00321802		lincRNA:chr6:64472442-64498999 reverse strand		1.31658	20.72898	2.25E-15
2332480	A_33_P3265254		Unknown		1.30304	20.09263	1.05E-15
882334	A_24_P203000	IL2RB	Homo sapiens interleukin 2 receptor, beta (IL2RB), mRNA [NM_00	NM_000878	1.2979	19.85643	3.00E-20
1140976	A_24_P30557	TBX5	Homo sapiens T-box 5 (TBX5), transcript variant 1, mRNA [NM_0C	NM_000192	1.2879	19.4043	5.91E-18
2336005	A_33_P3227736	C6orf227	Homo sapiens chromosome 6 open reading frame 227 (C6orf227),	NR_027908	1.28489	19.27038	2.05E-13
881201	A_24_P128713		ankyrin repeat domain 44 [Source:HGNC Symbol;Acc:25259] [ENS	AK097086	1.27919	19.01906	7.29E-20
2327680	A_19_P00321448		lincRNA:chr7:19970681-20008020 forward strand		1.25681	18.06401	8.06E-14
1160858	A_23_P307570	ABCC13	Homo sapiens ATP-binding cassette, sub-family C (CFTR/MRP),	NR_003087	1.2538	17.93913	9.39E-14
1162291	A_23_P215331	CRHR2	Homo sapiens corticotropin releasing hormone receptor 2 (CRHR:	NM_001883	1.24344	17.51615	9.67E-22
2333525	A_19_P00319066		lincRNA:chr8:122966846-123139423 reverse strand		1.23982	17.37085	1.17E-20
879191	A_23_P347610	HAVCR1	Homo sapiens hepatitis A virus cellular receptor 1 (HAVCR1), trar	NM_012206	1.19902	15.81309	1.62E-19
1141555	A_23_P385199	EPHA10	Homo sapiens EPH receptor A10 (EPHA10), transcript variant 2, r	NM_173641	1.19387	15.6269	1.17E-20
2312865	A_33_P3247589		Unknown		1.19194	15.55767	2.38E-12
2334800	A_33_P3723448	ART3	Homo sapiens ADP-ribosyltransferase 3 (ART3), transcript variar	NM_001130017	1.18942	15.46744	5.51E-16
880981	A_24_P25544	GDNF	Homo sapiens glial cell derived neurotrophic factor (GDNF), transc	NM_000514	1.18334	15.25245	9.11E-16
882326	A_23_P29608	IQCF5	Homo sapiens IQ motif containing F5 (IQCF5), mRNA [NM_001145	NM_001145059	1.15665	14.34336	6.08E-16
1166646	A_23_P38696	DSC1	Homo sapiens desmocollin 1 (DSC1), transcript variant Dsc1b, mR	NM_004948	1.10407	12.7078	7.13E-14
881712	A_24_P267592	SAMHD1	Homo sapiens SAM domain and HD domain 1 (SAMHD1), mRNA [I	NM_015474	1.08396	12.1327	9.89E-20
2323131	A_33_P3213617		V_segment translation product Fragment [Source:UniProtKB/TrE	A25493	1.06154	11.52233	5.76E-09
2327866	A_33_P3218316	VSTM2B	Homo sapiens V-set and transmembrane domain containing 2B (V	NM_001146339	1.05737	11.41215	7.72E-09
2325567	A_19_P00322088		lincRNA:chr16:11477618-11478868 reverse strand		1.02826	10.6724	2.13E-13
2312599	A_19_P00800410		lincRNA:chr18:68000270-68008995 reverse strand		0.99296	9.83931	1.85E-07
2312663	A_33_P3250899		Unknown		0.96346	9.193	6.84E-07
2333164	A_33_P3209135		Unknown		0.96037	9.12785	6.40E-07
2333382	A_33_P3875570	LOC100270679	UI-1-BC1p-akz-b-02-0-UI.s1 NCI_CGAP_Pl3 Homo sapiens cDN/	BQ013595	0.9556	9.02821	1.27E-06
1144094	A_23_P31798	NAT2	Homo sapiens N-acetyltransferase 2 (arylamine N-acetyltransfera	NM_000015	0.93999	8.70938	1.95E-20
2312753	A_33_P3264657	CYP27C1	Homo sapiens cytochrome P450, family 27, subfamily C, polypepti	NM_001001665	0.9384	8.67758	1.71E-14
1163074	A_32_P84373	FAM153C	Homo sapiens family with sequence similarity 153, member C (FA!	NM_001079527	0.9322	8.55454	3.17E-06
886971	A_23_P97402	CAMK1G	Homo sapiens calcium/calmodulin-dependent protein kinase IG (C	NM_020439	0.92428	8.40006	8.17E-06
2333100	A_33_P3227482	FLJ43944	Homo sapiens cDNA FLJ43944 fis, clone TESTI4014392. [AK1259	AK125932	0.90683	8.06916	2.86E-08
2326094	A_33_P3383189	SP9	Homo sapiens Sp9 transcription factor homolog (mouse) (SP9), ml	NM_001145250	0.8905	7.77147	7.10E-18
1142161	A_32_P23308	LOC257358	Homo sapiens hypothetical LOC257358 (LOC257358), non-coding	NR_026945	0.88489	7.6717	1.22E-06
887149	A_23_P381172	MRAP	Homo sapiens melanocortin 2 receptor accessory protein (MRAP)	NM_206898	0.88455	7.66574	2.23E-08
2325770	A_33_P3327642	AIM1L	Homo sapiens absent in melanoma 1-like (AIM1L), mRNA [NM_001	NM_001039775	0.88313	7.64073	0.00003

Supplementary Table 1. Continued.

2313354	A_19_P00328920		lincRNA:chr12:65991133-66071408 reverse strand		0.87642	7.52342	2.18E-08
2330096	A_33_P3305710		Unknown		0.87052	7.42204	0.00221
2326138	A_33_P3361067	ABCG2	Homo sapiens ATP-binding cassette, sub-family G (WHITE), meml	NM_004827	0.86983	7.41024	5.75E-19
2336019	A_33_P3465703	SNORA60	601888042F1 NIH_MGC_17 Homo sapiens cDNA clone IMAGE:412	BF304636	0.86634	7.35093	2.36E-16
2333099	A_33_P3872115	LOC727710	Homo sapiens cDNA FLJ25723 fis, clone TST05459. [AK098589]	AK098589	0.84797	7.04652	0.00002
1144506	A_23_P42897	MGAM	Homo sapiens maltase-glucoamylase (alpha-glucosidase) (MGAM)	NM_004668	0.83719	6.87368	0.00015
886684	A_23_P360924	CLDN17	Homo sapiens claudin 17 (CLDN17), mRNA [NM_012131]	NM_012131	0.83639	6.86101	0.00015
2333453	A_19_P00805888		lincRNA:chr6:28134146-28139947 forward strand		0.82915	6.74768	0.00019
2312797	A_19_P00804825		lincRNA:chr3:112308735-112319860 reverse strand		0.82638	6.70467	2.20E-13
1164351	A_24_P379353	CCDC24	Homo sapiens coiled-coil domain containing 24 (CCDC24), mRNA	NM_152499	0.81689	6.55973	8.25E-15
2312654	A_33_P3211968	LOC100133207	PREDICTED: Homo sapiens hypothetical LOC100133207 (LOC10C	XR_110242	0.81465	6.52607	0.00027
2323250	A_33_P3223022		CR745051 NCI_CGAP_Co16 Homo sapiens cDNA clone IMAGp971	CR745051	0.81331	6.50594	0.00035
2327782	A_19_P00329113		lincRNA:chr7:80367-94117 forward strand		0.81164	6.48101	1.00E-16
2313028	A_33_P3354688		full-length cDNA clone CS0DB006YB20 of Neuroblastoma Cot 10	CR625008	0.80785	6.42462	1.76E-16
2329974	A_33_P3346691		Unknown		0.80697	6.41165	0.00042
2332403	A_19_P00804120		lincRNA:chr13:41416375-41460125 forward strand		0.80402	6.36827	5.51E-13
2326173	A_33_P3362063	LOC440925	Homo sapiens hypothetical LOC440925 (LOC440925), non-coding	NR_027433	0.7888	6.14888	0.00075
1137521	A_24_P550924	BTBD19	Homo sapiens BTB (POZ) domain containing 19 (BTBD19), mRNA	NM_001136537	0.78811	6.13916	0.00065
880437	A_23_P47728	MAP6	Homo sapiens microtubule-associated protein 6 (MAP6), transcrip	NM_033063	0.78702	6.12378	4.34E-16
2327513	A_19_P00316061		lincRNA:chr1:234845129-234857092 forward strand		0.78532	6.09979	0.00077
2325759	A_33_P3310588		Unknown		0.7775	5.99106	0.00051
2313741	A_19_P00327103		lincRNA:chr3:37886971-37901969 forward strand		0.77346	5.93557	1.06E-14
2313088	A_33_P3341007		cDNA FLJ40133 fis, clone TESTI2012231 [Source:UniProtKB/Tr	XR_114873	0.7715	5.9088	1.34E-06
2313318	A_33_P3234482	LOC100132919	PREDICTED: Homo sapiens hypothetical protein LOC100132919 (	XM_001715268	0.7666	5.84245	0.00107
2332688	A_19_P00321586		lincRNA:chrX:97828872-98120013 forward strand		0.75881	5.73866	0.00144
2312990	A_33_P3246774	LOC649201	PREDICTED: Homo sapiens paraneoplastic antigen like 6A-like (L	XM_001127211	0.75574	5.69817	0.0006
2316995	A_33_P3210418	SLC5A10	Homo sapiens solute carrier family 5 (sodium/glucose cotransport	NM_152351	0.75466	5.68412	0.00174
889093	A_23_P167585	GDF9	Homo sapiens growth differentiation factor 9 (GDF9), mRNA [NM_	NM_005260	0.74921	5.61314	8.83E-07
2333946	A_33_P3251801	KLRG2	Homo sapiens killer cell lectin-like receptor subfamily G, member	NM_198508	0.74753	5.59149	0.00999
2312798	A_33_P3334000		Unknown		0.74628	5.57541	0.00031
880995	A_24_P216014	GH2	Homo sapiens growth hormone 2 (GH2), transcript variant 3, mRN	NM_022558	0.74281	5.53103	0.00004
2331548	A_19_P00810108		lincRNA:chrX:38716431-38792681 reverse strand		0.74192	5.5198	0.00053
2312771	A_33_P3416757	PRLR	Homo sapiens prolactin receptor (PRLR), mRNA [NM_000949]	NM_000949	0.73966	5.4911	0.00256
2325597	A_33_P3314352		Unknown		0.73913	5.48445	0.00268
2320385	A_33_P3327381		BX097352 Soares_testis_NHT Homo sapiens cDNA clone IMAGp9	BX097352	0.73722	5.46029	0.00279
2312879	A_19_P00318216		lincRNA:chr8:129022630-129113453 reverse strand		0.7359	5.44376	0.00262
2316883	A_33_P3209772	POU4F1	Homo sapiens POU class 4 homeobox 1 (POU4F1), mRNA [NM_00	NM_006237	0.73584	5.44301	0.00268
2314589	A_19_P00321664		lincRNA:chr4:6672454-6675524 forward strand		0.73433	5.42413	0.00003
1159480	A_24_P56330	C13orf16	Homo sapiens chromosome 13 open reading frame 16 (C13orf16),	NM_152324	0.72447	5.30233	0.0002
885381	A_23_P76322	PIK3C2G	Homo sapiens phosphoinositide-3-kinase, class 2, gamma polypep	NM_004570	0.72252	5.27856	0.00009
2313950	A_33_P3279276	GRIP2	Homo sapiens glutamate receptor interacting protein 2 (GRIP2), m	NM_001080423	0.72178	5.26958	0.00374
2330070	A_33_P3409458		Uncharacterized protein C9orf171 [Source:UniProtKB/Swiss-Prot;Acc:Q6ZQR2] [		0.71807	5.22485	5.95E-09
886914	A_23_P207879	CARD14	Homo sapiens caspase recruitment domain family, member 14 (CA	NM_024110	0.71643	5.20513	0.00475
2319089	A_19_P00800085		lincRNA:chrX:73422428-73423502 forward strand		0.71572	5.19664	0.00422
2312560	A_33_P3228985	LOC100132396	Homo sapiens zinc finger protein 705D-like (LOC100132396), mRI	NM_001193630	0.71147	5.14596	0.00463
2325771	A_33_P3570193	FLJ36000	Homo sapiens hypothetical FLJ36000 (FLJ36000), non-coding RN	NR_027084	0.71031	5.13225	0.0043
2327311	A_33_P3241184		Unknown		0.70549	5.07566	6.65E-15
2334089	A_33_P3231247	LOC727838	Putative uncharacterized protein ENSP00000391318 [Source:UniProtKB/TrEMBL:		0.70461	5.06534	0.00574
1151276	A_24_P870620	PTN	Homo sapiens pleiotrophin (PTN), mRNA [NM_002825]	NM_002825	0.70375	5.05535	0.00015
2315360	A_33_P3620832	ZNF483	Homo sapiens zinc finger protein 483 (ZNF483), transcript variant	NM_001007169	0.70133	5.0272	0.00022
2323266	A_33_P3219537	NCRNA00207	Homo sapiens non-protein coding RNA 207 (NCRNA00207), trans	NR_028409	0.69725	4.98025	0.00684
2323392	A_33_P3560176		BX096849 Soares_NFL_T_GBC_S1 Homo sapiens cDNA clone IMA	BX096849	0.69635	4.96994	0.00742
1162739	A_24_P84657	MUC2	Homo sapiens mucin 2, oligomeric mucus/gel-forming (MUC2), mF	NM_002457	0.69408	4.94399	0.00779
2333317	A_19_P00320499		lincRNA:chr6:132224787-132228642 forward strand		0.69133	4.91275	1.95E-11
2312796	A_19_P00320255		lincRNA:chr15:21165302-21166134 forward strand		0.68718	4.86608	0.00006
2314399	A_33_P3275702	FMO2	Homo sapiens flavin containing monooxygenase 2 (non-functional)	NM_001460	0.6861	4.85395	0.00032
1143034	A_23_P16694	CPS1-IT	Homo sapiens CPS1 intronic transcript (non-protein coding) (CPS	NR_002763	0.67543	4.73625	4.01E-07
2313011	A_19_P00804574		lincRNA:chr19:56789963-56824563 forward strand		0.67321	4.71206	0.00256
2316929	A_33_P3354553	LOC390638	PREDICTED: Homo sapiens Golgin subfamily A member 2-like (LC	XM_001715670	0.66397	4.61281	0.00156
1138131	A_32_P88415	MYOZ3	Homo sapiens myozenin 3 (MYOZ3), transcript variant 2, mRNA [N	NM_133371	0.65744	4.544	3.89E-14
2332836	A_33_P3369016		PREDICTED: Homo sapiens hypothetical LOC645685 (LOC64568	XM_928694	0.64815	4.44784	1.38E-06
2334995	A_19_P00808323		lincRNA:chr1:147706526-147727651 reverse strand		0.64539	4.41967	0.0031
2330315	A_19_P00321078		lincRNA:chr19:52588083-52597524 forward strand		0.64358	4.40131	6.63E-14
1157254	A_32_P155826	LOC158572	Homo sapiens hypothetical LOC158572 (LOC158572), non-coding	NR_026742	0.64341	4.39954	0.0052
2322691	A_33_P3372212	PSG10P	Human processed pseudo-pregnancy-specific glycoprotein (PSG1	L14723	0.6425	4.39038	0.00847
2330071	A_33_P3375269		Unknown		0.64208	4.38617	0.00704
1138097	A_24_P928969	PTPN3	Homo sapiens protein tyrosine phosphatase, non-receptor type 3	NM_001145369	0.63754	4.34054	0.0016
2330192	A_19_P00328485		lincRNA:chr5:124817426-124830024 reverse strand		0.6214	4.18219	3.29E-09
2327713	A_33_P3305058		Homo sapiens cDNA FLJ43238 fis, clone HCHON2007881. [AK12	AK125228	0.61905	4.1596	3.58E-06
2328986	A_19_P00331229		lincRNA:chr8:17355272-17386123 forward strand		0.61285	4.10059	5.41E-10
1159420	A_23_P418485	C11orf65	Homo sapiens chromosome 11 open reading frame 65 (C11orf65),	NM_152587	0.61224	4.09491	0.00287
2329853	A_19_P00803281		lincRNA:chr12:3723064-3743064 forward strand		0.60759	4.05124	0.00015

Supplementary Table 1. Continued.

2326590	A_33_P3398187		Unknown		0.60387	4.01672	0.0047
2327555	A_33_P3215834	SORCS2	Homo sapiens sortilin-related VPS10 domain containing receptor :	NM_020777	0.60247	4.00378	0.00895
2318439	A_33_P3403595		Putative uncharacterized protein FLJ46792 [Source:UniProtKB/S	AK128633	0.60062	3.98676	0.00405
2319616	A_33_P3353996	PPP1R3G	Homo sapiens protein phosphatase 1, regulatory (inhibitor) subunit	NM_001145115	0.59606	3.94509	3.49E-06
1145452	A_23_P23646	USF1	Homo sapiens upstream transcription factor 1 (USF1), transcript v	NM_007122	0.59575	3.94228	2.38E-15
1143061	A_23_P91317	RAD21L1	Homo sapiens RAD21-like 1 (S. pombe) (RAD21L1), mRNA [NM_0(	NM_001136566	0.59544	3.93949	0.0001
888760	A_23_P134139	FABP7	Homo sapiens fatty acid binding protein 7, brain (FABP7), mRNA [	NM_001446	0.58451	3.84158	5.31E-08
882700	A_24_P181254	OLFM4	Homo sapiens olfactomedin 4 (OLFM4), mRNA [NM_006418]	NM_006418	0.58423	3.83914	0.00633
2313080	A_19_P00320641		lincRNA:chr16:72557245-72661585 reverse strand		0.58334	3.83123	1.60E-12
2331529	A_19_P00318297		lincRNA:chr7:77287012-77290598 reverse strand		0.58223	3.82146	0.00716
1155912	A_23_P114210	POU3F4	Homo sapiens POU class 3 homeobox 4 (POU3F4), mRNA [NM_00	NM_000307	0.58178	3.81748	0.00546
1142763	A_23_P2317	DDN	Homo sapiens dendrin (DDN), mRNA [NM_015086]	NM_015086	0.58153	3.81533	6.79E-06
2322379	A_33_P3331335	TET3	Homo sapiens tet oncogene family member 3 (TET3), mRNA [NM_	NM_144993	0.57871	3.7906	0.00673
1160842	A_23_P163697	SYT17	Homo sapiens synaptotagmin XVII (SYT17), mRNA [NM_016524]	NM_016524	0.57551	3.76279	4.36E-09
2316961	A_33_P3224190	SLC29A4	Homo sapiens solute carrier family 29 (nucleoside transporters), n	NM_001040661	0.57061	3.72054	0.00343
2316904	A_19_P00809247		lincRNA:chr13:91173499-91254624 reverse strand		0.55888	3.62139	0.00045
2314494	A_33_P3409493	LOC285095	PREDICTED: Homo sapiens hypothetical protein LOC285095, tran	XM_001722643	0.55883	3.62104	0.00598
2325438	A_33_P3299634	OR1L6	olfactory receptor, family 1, subfamily L, member 6 [Source:HGNC	AB529307	0.55478	3.58739	0.00003
1161185	A_23_P203267	TRIM29	Homo sapiens tripartite motif-containing 29 (TRIM29), mRNA [NM	NM_012101	0.5537	3.57851	0.00097
2313814	A_19_P00321176		lincRNA:chrX:110799569-110812724 reverse strand		0.55023	3.55004	0.0003
2319663	A_33_P3254579	KRTAP1-5	Homo sapiens keratin associated protein 1-5 (KRTAP1-5), mRNA	NM_031957	0.54988	3.54713	0.00146
2324441	A_19_P00324820		lincRNA:chr2:47735821-47746846 forward strand		0.54711	3.5246	0.00017
2315999	A_19_P00331971		lincRNA:chrX:133675909-133682959 reverse strand		0.54292	3.49072	0.00469
2317444	A_33_P3354574		Unknown		0.54224	3.48528	0.00452
2325421	A_33_P3221665	KIF21B	Homo sapiens kinesin family member 21B (KIF21B), mRNA [NM_01	NM_017596	0.53208	3.40472	0.00684
2326242	A_19_P00316995		lincRNA:chr17:75084810-75085353 reverse strand		0.52557	3.35402	0.00961
884722	A_23_P427645	TLL1	Homo sapiens tolloid-like 1 (TLL1), mRNA [NM_012464]	NM_012464	0.52538	3.35262	0.00538
1161527	A_32_P191840	LOC644662	PREDICTED: Homo sapiens hypothetical protein LOC644662, tran	XM_933803	0.52132	3.32141	0.00005
2333735	A_19_P00810231		lincRNA:chr12:47044233-47098483 forward strand		0.51507	3.27393	0.00011
2316759	A_33_P3264790	GJD4	Homo sapiens gap junction protein, delta 4, 40.1kDa, mRNA (cDNA	BC035898	0.51161	3.24796	0.00492
2327761	A_33_P3250555		Uncharacterized protein C18orf2 [Source:UniProtKB/Swiss-Prot	AF295728	0.511	3.24342	0.00006
1138046	A_24_P125561	SIGLEC9	Homo sapiens sialic acid binding Ig-like lectin 9 (SIGLEC9), transc	NM_014441	0.50805	3.22143	0.00828
2331899	A_19_P00329800		lincRNA:chr1:204525202-204541352 forward strand		0.50568	3.20392	5.28E-11
883932	A_23_P128503	IL26	Homo sapiens interleukin 26 (IL26), mRNA [NM_018402]	NM_018402	0.50431	3.19385	0.00266
2329975	A_33_P3277808		Unknown		0.49735	3.14301	5.96E-07
2328457	A_33_P3404489	MAMLD1	Homo sapiens mastermind-like domain containing 1 (MAMLD1), tr	NM_001177465	0.49719	3.1419	0.00356
2313311	A_33_P3226557	PRR23B	Homo sapiens proline rich 23B (PRR23B), mRNA [NM_001013650]	NM_001013650	0.49667	3.13815	0.00136
2316784	A_33_P3348794		UPF0627 protein ENSP00000341061/ENSP00000339743 [Source	XM_001130683	0.49426	3.12072	0.00236
1141675	A_32_P78101	IGSF21	Homo sapiens immunoglobin superfamily, member 21 (IGSF21), mF	NM_032880	0.49198	3.10445	1.54E-06
2316263	A_19_P00810328		lincRNA:chr16:11468274-11482224 forward strand		0.47578	2.99072	0.00928
2323865	A_19_P00802508		lincRNA:chr2:12453499-12466124 forward strand		0.47354	2.9754	0.0006
2320947	A_33_P3316728		Putative GED domain-containing protein LOC554175 [Source:Uni	BC006530	0.47309	2.97231	0.00667
2332940	A_33_P3339980	LOC100128429	Homo sapiens cDNA FLJ41329 fis, clone BRAMY2047676. [AK123	AK123323	0.47156	2.9618	4.49E-06
2315276	A_33_P3265866		Unknown		0.46857	2.94149	9.21E-09
2325561	A_19_P00803876		lincRNA:chr2:235342486-235378011 forward strand		0.46497	2.91721	5.74E-10
2319756	A_33_P3329241		DA734585 NT2RP2 Homo sapiens cDNA clone NT2RP2005523 5',	DA734585	0.46472	2.91553	0.00805
2319233	A_33_P3249958	LOC642852	Homo sapiens hypothetical LOC642852 (LOC642852), non-coding	NR_026943	0.46408	2.91128	0.00925
2312922	A_33_P3232273		Unknown		0.46397	2.91051	0.00145
2312878	A_19_P00810938		lincRNA:chr6:3176201-3194126 forward strand		0.46309	2.9046	0.00015
1155450	A_23_P399797	SMAD5OS	Homo sapiens SMAD family member 5 opposite strand (SMAD5OS	NR_026763	0.46041	2.88678	0.0074
1160116	A_23_P164068	DNAH9	Homo sapiens dynein, axonemal, heavy chain 9 (DNAH9), transcrip	NM_001372	0.45863	2.87493	0.00873
2335739	A_33_P3380196	AKAP9	Homo sapiens A kinase (PRKA) anchor protein (yotiao) 9, mRNA (	BC015533	0.45543	2.85384	0.00114
2321653	A_33_P3417487		signal peptide, CUB domain, EGF-like 1 [Source:HGNC Symbol;Ac	BC047916	0.44854	2.80889	0.0003
1168308	A_32_P925529		PREDICTED: Homo sapiens similar to core 1 synthase, glycoprote	XR_015594	0.44843	2.8082	0.00208
1140995	A_23_P254353	NOXA1	Homo sapiens NADPH oxidase activator 1 (NOXA1), mRNA [NM_0	NM_006647	0.44784	2.80439	2.59E-09
2319700	A_33_P3394175		V_segment translation product Fragment [Source:UniProtKB/TrE	AB308151	0.4452	2.78741	0.00883
1139044	A_24_P28722	RSAD2	Homo sapiens radical S-adenosyl methionine domain containing 2	NM_080657	0.4421	2.76759	0.00605
2313824	A_33_P3651282	SNORA20	HHAGE017587 Human liver regeneration after partial hepatectom	DW416857	0.43962	2.75185	3.95E-09
2322438	A_33_P3307490		Homo sapiens cDNA FLJ45043 fis, clone BRAWH3021012. [AK126	AK126988	0.43883	2.7468	0.0049
880992	A_23_P69497	CLEC3B	Homo sapiens C-type lectin domain family 3, member B (CLEC3B	NM_003278	0.43687	2.73446	0.00014
2328895	A_19_P00801014		lincRNA:chr4:80995226-81008001 reverse strand		0.4357	2.72706	0.00154
2334031	A_33_P3714482	LRP5L	Homo sapiens low density lipoprotein receptor-related protein 5-l	NM_001135772	0.43316	2.71116	0.00026
2331720	A_19_P00315637		lincRNA:chr2:120966064-120966496 reverse strand		0.43198	2.70382	0.0054
2326155	A_33_P3255716	LOC285095	PREDICTED: Homo sapiens hypothetical protein LOC285095, tran	XM_001722643	0.42766	2.67705	5.25E-09
888487	A_23_P3379	RASGRF1	Homo sapiens Ras protein-specific guanine nucleotide-releasing f	NM_002891	0.42619	2.66805	0.0055
2331799	A_19_P00330435		lincRNA:chr7:79083614-79104439 forward strand		0.42614	2.66772	0.00215
2323910	A_19_P00805265		lincRNA:chr6:29701971-29740296 forward strand		0.42401	2.65468	0.00013
2312799	A_33_P3370265	LOC100508645	PREDICTED: Homo sapiens hypothetical protein LOC100508645 (	XM_003120021	0.42194	2.64206	0.00193
2312726	A_33_P3298120	HEPN1	Homo sapiens HEPACAM opposite strand 1 (HEPN1), mRNA [NM_	NM_001037558	0.42125	2.63786	0.00306
2327926	A_19_P00802957		lincRNA:chr10:1028350-1033600 forward strand		0.42057	2.63369	0.00991
2335265	A_33_P3360530		Unknown		0.41944	2.62685	0.00905
1140073	A_23_P431505	XKR6	Homo sapiens primary neuroblastoma cDNA, clone:Nbla00437, full	AB073660	0.41583	2.60513	9.47E-08

Supplementary Table 1. Continued.

2332523	A_33_P3366391		Putative uncharacterized protein ENSP00000339838 [Source:Unil	AK128036	0.41414	2.59504	1.27E-08
2326983	A_33_P3271470	GREB1L	Homo sapiens growth regulation by estrogen in breast cancer-like	NM_001142966	0.41383	2.59314	0.00151
2322332	A_19_P00322412		lincRNA:chr6:6794382-6801404 reverse strand		0.4137	2.5924	0.00161
2336174	A_33_P3380652	ADAM28	Homo sapiens ADAM metallopeptidase domain 28 (ADAM28), tran:	NM_021777	0.41315	2.58914	0.00283
887463	A_23_P14986	HSD11B2	Homo sapiens hydroxysteroid (11-beta) dehydrogenase 2 (HSD11I	NM_000196	0.40867	2.56255	0.00433
2330229	A_19_P00810792		lincRNA:chr11:39499974-40108074 forward strand		0.40573	2.54528	8.07E-07
2328536	A_19_P00320716		lincRNA:chr3:106830589-106959470 reverse strand		0.40388	2.53445	0.0065
2319080	A_33_P3541279		Homo sapiens PRO0566 mRNA, complete cds. [AF116649]	AF116649	0.39892	2.50565	0.00595
2321635	A_19_P00318451		lincRNA:chr8:30724887-30746222 reverse strand		0.39398	2.47728	0.00709
2320762	A_19_P00324706		lincRNA:chr11:72446627-72462602 forward strand		0.39369	2.47567	0.00269
1154014	A_24_P917819	ANKRD30BP2	Homo sapiens ankyrin repeat domain 30B pseudogene 2 (ANKRD3	NR_026916	0.39279	2.47054	4.43E-08
2314385	A_19_P00329774		lincRNA:chr7:33679675-33695300 forward strand		0.39221	2.46723	3.57E-08
1150025	A_23_P68998	MIOX	Homo sapiens myo-inositol oxygenase (MIOX), mRNA [NM_017584	NM_017584	0.39103	2.46056	4.95E-12
2329461	A_19_P00801430		lincRNA:chr5:148229632-148269232 reverse strand		0.39023	2.45603	0.00032
1150153	A_32_P62090		Coiled-coil domain-containing protein ENSP00000299415 [Source:UniProtKB/Swi		0.38983	2.45373	0.00566
1143083	A_24_P357726	PSG8	Homo sapiens pregnancy specific beta-1-glycoprotein 8 (PSG8), t	NM_182707	0.38714	2.4386	0.00241
2328112	A_33_P3291160		Unknown		0.38691	2.4373	9.68E-06
2319702	A_19_P00809537		lincRNA:chr3:129614085-129688060 reverse strand		0.38492	2.42616	0.00968
1160504	A_24_P233078	PYY2	Homo sapiens peptide YY, 2 (seminalplasmin) (PYY2), non-coding	NR_003064	0.38252	2.41282	6.35E-08
2330389	A_33_P3304764		Unknown		0.38209	2.41038	0.00002
883692	A_23_P62588	CALML6	Homo sapiens calmodulin-like 6 (CALML6), mRNA [NM_138705]	NM_138705	0.38126	2.40579	0.00226
2322641	A_19_P00808575		lincRNA:chr14:23018310-23025460 reverse strand		0.38122	2.4056	0.00197
2312824	A_33_P3418571		Unknown		0.38101	2.40442	0.00107
1144010	A_23_P66328	ACSM2B	Homo sapiens acyl-CoA synthetase medium-chain family member	NM_182617	0.38073	2.40285	0.00184
2316143	A_33_P3287907	LOC728723	Homo sapiens hypothetical LOC728723 (LOC728723), non-coding	NR_024398	0.37868	2.39153	0.00486
1155758	A_23_P44867	AMBN	Homo sapiens ameloblastin (enamel matrix protein) (AMBN), mRN/	NM_016519	0.37774	2.38638	0.00281
2325472	A_33_P3210368		microRNA 762 [Source:HGNC Symbol;Acc:37303] [ENST0000038(	BC058863	0.37759	2.38558	0.00133
2325163	A_33_P3332166	PRSS38	Homo sapiens protease, serine, 38 (PRSS38), mRNA [NM_183062]	NM_183062	0.376	2.37682	0.00008
2334665	A_33_P3354514	SLC2A13	Homo sapiens solute carrier family 2 (facilitated glucose transport	BC047507	0.37463	2.36936	0.00895
1145578	A_32_P223173		PREDICTED: Homo sapiens similar to acetyl-Coenzyme A acyltrai	XR_037043	0.37456	2.36899	2.55E-07
2323429	A_19_P00331284		lincRNA:chr8:29414656-29675506 forward strand		0.3737	2.36427	0.00468
2317137	A_33_P3276087		Unknown		0.37347	2.36304	0.00201
2314843	A_19_P00325961		lincRNA:chr8:28912381-28922959 forward strand		0.37304	2.36068	1.11E-07
2322761	A_19_P00805134		lincRNA:chr12:105804881-105805536 reverse strand		0.37222	2.35627	0.00171
2326327	A_33_P3413353		Unknown		0.36624	2.32401	0.00597
2318395	A_33_P3382849	ACR	Homo sapiens acrosin (ACR), mRNA [NM_001097]	NM_001097	0.36559	2.32055	0.00048
2329503	A_33_P3278916		PHD finger protein 20-like 1 [Source:HGNC Symbol;Acc:24280] [ENST0000036199		0.36323	2.30799	0.0017
2332723	A_19_P00803767		lincRNA:chr8:110172174-110224124 reverse strand		0.35919	2.28662	0.00919
2315136	A_33_P3381613		Unknown		0.35668	2.27342	0.00442
1143491	A_23_P117851	CPLX3	Homo sapiens complexin 3 (CPLX3), mRNA [NM_001030005]	NM_001030005	0.3551	2.26515	0.00138
2333251	A_19_P00326790		lincRNA:chr7:106355964-106363089 reverse strand		0.35506	2.26494	0.00007
1136694	A_32_P68942	C21orf81	Homo sapiens chromosome 21 open reading frame 81 (C21orf81),	NR_027270	0.35251	2.25169	0.00137
2313552	A_33_P3321996		DB053889 TESTI2 Homo sapiens cDNA clone TESTI2044585 5', n	DB053889	0.35029	2.24021	0.00008
2322385	A_19_P00800632		lincRNA:chr2:152619954-152626029 reverse strand		0.34947	2.236	5.15E-06
2314133	A_33_P3233731		PREDICTED: Homo sapiens hypothetical LOC100129238 (LOC10C	XM_001716016	0.3487	2.23202	0.00228
2325505	A_33_P3415617		Unknown		0.34833	2.23011	7.18E-07
2326408	A_33_P3310548	LOC100131131	PREDICTED: Homo sapiens AHPA9419 (LOC100131131), miscRN/	XR_109928	0.34823	2.22959	0.0025
2322354	A_33_P3314531	LOC646853	PREDICTED: Homo sapiens non-histone chromosomal protein HM	XM_001719033	0.34744	2.22556	0.00004
2332029	A_33_P3221177	ZDHHC20	Homo sapiens zinc finger, DHHC-type containing 20 (ZDHHC20), ı	NM_153251	0.34698	2.2232	5.19E-06
2312945	A_19_P00809185		lincRNA:chr19:15400850-15442450 forward strand		0.34687	2.22264	0.00094
2315751	A_33_P3388983	C5orf56	Homo sapiens cDNA: FLJ21568 fis, clone COL06492. [AK025221]	AK025221	0.34608	2.21858	0.00039
2316950	A_19_P00331084		lincRNA:chr2:203179905-203194480 forward strand		0.34577	2.21701	0.00693
2313658	A_33_P3231407		pyrin and HIN domain family, member 1 [Source:HGNC Symbol;Aci	AK308350	0.34439	2.20999	0.00078
2320766	A_33_P3805839		Unknown		0.34402	2.20811	6.24E-06
2335186	A_19_P00331406		lincRNA:chr6:106935857-106946432 forward strand		0.34279	2.20186	1.04E-06
881282	A_23_P9883	NLRP3	Homo sapiens NLR family, pyrin domain containing 3 (NLRP3), trai	NM_001079821	0.34214	2.19858	0.00184
2328606	A_19_P00320570		lincRNA:chr5:158848221-158984695 forward strand		0.34113	2.19344	8.23E-07
879351	A_23_P398566	NR4A3	Homo sapiens nuclear receptor subfamily 4, group A, member 3 (N	NM_006981	0.3411	2.1933	7.47E-11
2313742	A_33_P3347152		suppressor of Ty 3 homolog (S. cerevisiae) [Source:HGNC Symbol;Acc:11466] [EN:		0.34025	2.18902	0.00099
2325487	A_19_P00800754		lincRNA:chr17:67543555-67550405 reverse strand		0.33938	2.18462	4.23E-06
2323989	A_19_P00320309		lincRNA:chr21:43099535-43117496 reverse strand		0.33901	2.18277	1.29E-06
1152893	A_32_P212095	C17orf105	Homo sapiens chromosome 17 open reading frame 105 (C17orf10!	NM_001136483	0.339	2.18271	0.0001
2320870	A_19_P00323081		lincRNA:chr3:156477781-156539131 forward strand		0.33886	2.18205	0.00382
2318436	A_33_P3278813	LOC100127885	PREDICTED: Homo sapiens hypothetical protein LOC100127885 (	XM_001721771	0.33814	2.17843	0.00054
1154483	A_23_P47709	FOLR2	Homo sapiens folate receptor 2 (fetal) (FOLR2), transcript variant	NM_000803	0.33719	2.17365	0.00139
2317260	A_19_P00802550		lincRNA:chr12:105804881-105805536 forward strand		0.33681	2.17176	0.00001
1139580	A_32_P129950	NHLRC3	Homo sapiens NHL repeat containing 3 (NHLRC3), transcript varia	NM_001012754	0.3317	2.14633	1.50E-06
2320343	A_33_P3219146	ODF3	Homo sapiens outer dense fiber of sperm tails 3 (ODF3), mRNA [I	NM_053280	0.32999	2.13789	0.00052
2314360	A_33_P3514487	VSTM1	Homo sapiens V-set and transmembrane domain containing 1 (VS	NM_198481	0.32927	2.13438	0.00917
2318119	A_33_P3882624	POT1	Homo sapiens protection of telomeres 1 homolog (S. pombe) (PO1	NM_015450	0.32774	2.12688	1.48E-11
1152815	A_24_P361643	C19orf55	Homo sapiens chromosome 19 open reading frame 55 (C19orf55),	NM_001039887	0.32565	2.11664	0.00037
2329764	A_19_P00330855		lincRNA:chr6:168670126-168683226 forward strand		0.32317	2.10461	0.00387

Supplementary Table 1. Continued.

2314125	A_19_P00318029		lincRNA:chr9:137444583-137445743 forward strand		0.32313	2.10442	0.00145
1162804	A_23_P17663	MX1	Homo sapiens myxovirus (influenza virus) resistance 1, interferon-	NM_002462	0.32132	2.09568	2.20E-10
2332754	A_19_P00802049		lincRNA:chr14:28091410-28141510 reverse strand		0.31877	2.0834	0.00756
2332592	A_33_P3252134		Unknown		0.31868	2.08297	2.85E-06
2321752	A_19_P00809022		lincRNA:chr16:88737774-88742899 reverse strand		0.31804	2.07991	0.00679
2332655	A_33_P3218960	CACNA1H	Homo sapiens calcium channel, voltage-dependent, T type, alpha	NM_021098	0.31718	2.07576	0.0038
2326689	A_33_P3331326		Unknown		0.31495	2.06512	3.46E-06
2316027	A_33_P3309429		Unknown	BC039333	0.31399	2.06058	3.73E-06
2333524	A_33_P3248602	DUX4	Homo sapiens double homeobox 4 (DUX4), mRNA [NM_033178]	NM_033178	0.31287	2.05529	6.37E-06
2315323	A_19_P00808893		lincRNA:chr5:94005444-94012744 forward strand		0.31149	2.04877	0.00346
2317836	A_19_P00326099		lincRNA:chr14:96344047-96390722 forward strand		0.3101	2.04223	0.00003
888672	A_23_P156697	ABHD16A	Homo sapiens abhydrolase domain containing 16A (ABHD16A), tra	NM_021160	0.30987	2.04114	7.47E-06
2329097	A_33_P3254320	SH2D3A	Homo sapiens SH2 domain containing 3A (SH2D3A), mRNA [NM_0	NM_005490	0.30961	2.03989	0.00493
1158900	A_24_P232790	C14orf162	Homo sapiens chromosome 14 open reading frame 162 (C14orf16:	NR_024630	0.30915	2.03775	4.84E-06
2318877	A_19_P00323658		lincRNA:chr1:204566252-204573127 forward strand		0.30886	2.0364	0.00537
2336146	A_19_P00331743		lincRNA:chr6:41411797-41426472 reverse strand		0.30777	2.03129	0.00013
882169	A_24_P606538	GGNBP1	Homo sapiens gametogenetin binding protein 1 (GGNBP1), non-co	NR_028361	0.30746	2.02983	0.00015
2324990	A_19_P00325872		lincRNA:chr11:9781624-9791949 reverse strand		0.30745	2.02979	0.00325
2327409	A_19_P00800013		lincRNA:chr5:88237556-88240804 forward strand		0.30468	2.0169	0.00638
2333402	A_19_P00317337		lincRNA:chr2:43325013-43329583 reverse strand		0.30458	2.01642	0.00944
2320721	A_19_P00808808		lincRNA:chr5:127385751-127405576 reverse strand		0.30413	2.01433	0.00136
2327489	A_33_P3246193		Uncharacterized protein C10orf92 [Source:UniProtKB/Swiss-Pro	XM_003119477	0.30395	2.01348	0.00855
2335735	A_33_P3287731	MYST4	Homo sapiens MYST histone acetyltransferase (monocytic leukem	NM_012330	0.30358	2.01176	0.00483
2332117	A_33_P3219121		Homo sapiens cDNA FLJ16443 fis, clone BRAMY3004800, highly	AK131379	0.30343	2.01108	0.00374
1145392	A_23_P355776		non-protein coding RNA 266B [Source:HGNC Symbol;Acc:38529]	XM_002342011	0.30248	2.00669	0.00699
2319524	A_19_P00321826		lincRNA:chr20:37049238-37049736 forward strand		0.30206	2.00474	0.00373
2315891	A_33_P3387429		PREDICTED: Homo sapiens similar to translocase of inner mitochi	XR_019644	0.30155	2.00238	0.00407
2320504	A_19_P00317913		lincRNA:chr6:29706528-29716826 reverse strand		0.30121	2.00084	0.00805
2333066	A_33_P3400309		Homo sapiens cDNA FLJ40251 fis, clone TESTI2024283. [AK0975	AK097570	-0.3011	-2.00034	0.00137
2323230	A_19_P00806601		lincRNA:chr8:67304071-67319246 forward strand		-0.30233	-2.006	0.00436
2316746	A_33_P3229809	FLJ25363	Homo sapiens similar to hypothetical protein FLJ25976 (FLJ2536:	NM_001145553	-0.3024	-2.00632	0.00267
2320006	A_33_P3323939	LIPN	Homo sapiens lipase, family member N (LIPN), mRNA [NM_001102	NM_001102469	-0.30316	-2.00985	0.00002
2319827	A_33_P3236416	GPR179	Homo sapiens G protein-coupled receptor 179 (GPR179), mRNA [	NM_001004334	-0.30334	-2.01068	0.00222
2324373	A_19_P00321710		lincRNA:chr7:32957013-32961211 forward strand		-0.30345	-2.01116	0.00797
2324141	A_33_P3418511	HTA	Homo sapiens hypothetical LOC283902 (HTA), non-coding RNA [l	NR_027756	-0.30442	-2.0157	0.00209
1166480	A_24_P48627	PIAS4	protein inhibitor of activated STAT, 4 [Source:HGNC Symbol;Acc:	AK022481	-0.30447	-2.01592	2.05E-10
2332902	A_19_P00812871		lincRNA:chr14:74163472-74168555 reverse strand		-0.30526	-2.01958	0.00289
2331007	A_19_P00327864		lincRNA:chr12:48153483-48166083 reverse strand		-0.30568	-2.02151	0.0056
1159679	A_23_P71328	MATN2	Homo sapiens matrilin 2 (MATN2), transcript variant 2, mRNA [NM	NM_030583	-0.30572	-2.0217	0.00448
886189	A_23_P127697		olfactory receptor, family 5, subfamily AQ, member 1 pseudogene	AB529247	-0.30588	-2.02247	0.00849
1163553	A_23_P386320	MFI2	Homo sapiens antigen p97 (melanoma associated) identified by mc	NM_033316	-0.30595	-2.02281	0.00476
2326104	A_33_P3268234	KRT39	Homo sapiens keratin 39 (KRT39), mRNA [NM_213656]	NM_213656	-0.30805	-2.0326	0.00569
2312858	A_19_P00802546		lincRNA:chrX:103436994-103445194 forward strand		-0.3084	-2.03422	5.64E-06
2315997	A_19_P00806685		lincRNA:chr5:85742844-85867069 forward strand		-0.30912	-2.03762	0.00015
2321196	A_33_P3324655	C8orf41	Homo sapiens chromosome 8 open reading frame 41 (C8orf41), tr	NM_025115	-0.30936	-2.03872	8.06E-06
880762	A_23_P8913	CA2	Homo sapiens carbonic anhydrase II (CA2), mRNA [NM_000067]	NM_000067	-0.31018	-2.04259	0.00003
2317667	A_19_P00330453		lincRNA:chr8:69903546-69930946 forward strand		-0.31064	-2.04474	0.00037
1158004	A_24_P417586	FLYWCH2	Homo sapiens FLYWCH family member 2 (FLYWCH2), transcript v	NM_138439	-0.31304	-2.05609	9.37E-06
1150076	A_32_P144342	PARP4	Homo sapiens poly (ADP-ribose) polymerase family, member 4 (P/	NM_006437	-0.31308	-2.05629	7.73E-11
2336190	A_19_P00320927		lincRNA:chr12:1612439-1613590 forward strand		-0.31309	-2.05632	0.00003
2321673	A_19_P00320725		lincRNA:chr2:224569462-224588757 reverse strand		-0.31318	-2.05677	0.0011
880364	A_24_P150791	JPH3	Homo sapiens junctophilin 3 (JPH3), mRNA [NM_020655]	NM_020655	-0.31472	-2.06404	0.0051
2328505	A_33_P3219454		Unknown		-0.31489	-2.06488	3.46E-06
2328114	A_33_P3251640	LOC284440	Homo sapiens hypothetical LOC284440 (LOC284440), non-coding	NR_026956	-0.31516	-2.06614	0.0014
1137083	A_23_P28042	ZNF737	Homo sapiens zinc finger protein 737 (ZNF737), mRNA [NM_00115	NM_001159293	-0.31569	-2.06866	3.53E-06
1156743	A_24_P353619	ALPL	Homo sapiens alkaline phosphatase, liver/bone/kidney (ALPL), tra	NM_000478	-0.31614	-2.07083	1.27E-06
1142797	A_32_P187663	ZNF596	Homo sapiens zinc finger protein 596 (ZNF596), transcript variant	NM_001042416	-0.3171	-2.07541	6.29E-06
2321381	A_19_P00316050		lincRNA:chr7:130597213-130598373 forward strand		-0.3172	-2.07588	0.0078
885220	A_23_P79360	NOSTRIN	Homo sapiens nitric oxide synthase trafficker (NOSTRIN), transcri	NM_052946	-0.31759	-2.07774	0.00333
2312589	A_33_P3254751	LOC100131355	Homo sapiens cDNA FLJ42223 fis, clone THYMU2039989. [AK124	AK124217	-0.31775	-2.07848	6.25E-06
2324016	A_33_P3324228		similar to hCG1645603 (LOC732275), non-coding RNA [Source:Re	AL137562	-0.31809	-2.08012	0.00476
1139911	A_24_P136866	SLC8A1	Homo sapiens solute carrier family 8 (sodium/calcium exchanger),	NM_021097	-0.31844	-2.08179	0.00161
1147915	A_23_P419764		Homo sapiens mRNA; cDNA DKFZp761B107 (from clone DKFZp7t	AL834257	-0.31915	-2.0852	9.43E-06
1146751	A_23_P251881	NCR3	Homo sapiens natural cytotoxicity triggering receptor 3 (NCR3), tr	NM_147130	-0.31926	-2.08573	0.00003
2325344	A_33_P3345623	RREB1	Homo sapiens ras responsive element binding protein 1 (RREB1), t	NM_001003700	-0.31938	-2.08632	0.00049
1160389	A_23_P340019	NLRC3	Homo sapiens NLR family, CARD domain containing 3 (NLRC3), m	NM_178844	-0.3198	-2.08832	0.00004
2313683	A_33_P3276758		Unknown		-0.32027	-2.09059	0.00277
2329943	A_19_P00806723		lincRNA:chr16:72454899-72460874 forward strand		-0.32097	-2.09397	0.00157
2326697	A_19_P00812923		lincRNA:chr11:3402374-3408174 reverse strand		-0.32205	-2.09919	0.00129
2330783	A_19_P00803630		lincRNA:chr8:2522118-2527693 forward strand		-0.32471	-2.11206	2.86E-06
1154776	A_23_P377245		Homo sapiens full length insert cDNA clone YR69B11. [AF085941]	BC042172	-0.32542	-2.11551	8.99E-06
2319965	A_19_P00330834		lincRNA:chr10:65236069-65272719 forward strand		-0.3256	-2.11643	4.92E-06

Supplementary Table 1. Continued.

884591	A_23_P14892	IGFALS	Homo sapiens insulin-like growth factor binding protein, acid labile	NM_004970	-0.32607	-2.11872	0.00959
2312644	A_33_P3333033	SGSM2	Homo sapiens cDNA FLJ42893 fis, clone BRHIP3008598. [AK124§	AK124883	-0.32609	-2.11882	1.79E-06
1152414	A_24_P122636	BPNT1	Homo sapiens 3'(2'), 5'-bisphosphate nucleotidase 1 (BPNT1), mR	NM_006085	-0.32649	-2.12075	2.32E-06
2319047	A_33_P3345309	LOC646960	Homo sapiens trypsin serine protease-like (LOC646960), mRNA [I	NM_001195129	-0.32649	-2.12077	0.00358
2326452	A_19_P00812589		lincRNA:chr13:36789625-36798125 forward strand		-0.32725	-2.12446	0.0007
2313913	A_33_P3356811	LCE1E	Homo sapiens late cornified envelope 1E (LCE1E), mRNA [NM_17§	NM_178353	-0.32778	-2.12708	1.64E-06
1139320	A_23_P13683	ZBTB39	Homo sapiens zinc finger and BTB domain containing 39 (ZBTB39	NM_014830	-0.32802	-2.12824	5.17E-06
2316464	A_33_P3235706		zinc finger, CCHC domain containing 11 [Source:HGNC Symbol;Ac	BC048301	-0.32833	-2.12976	0.00001
2318598	A_33_P3343605		Unknown		-0.32915	-2.13378	1.51E-06
2335379	A_33_P3351536	PTK2B	Homo sapiens PTK2B protein tyrosine kinase 2 beta (PTK2B), trai	NM_173174	-0.32966	-2.13631	0.00865
2334377	A_19_P00806946		lincRNA:chr1:173758702-173765627 reverse strand		-0.33055	-2.14066	0.00032
2320523	A_19_P00810827		lincRNA:chr1:192885227-192945802 forward strand		-0.3312	-2.14386	0.00252
2329440	A_19_P00327017		lincRNA:chr8:28543756-28558056 reverse strand		-0.33165	-2.14611	0.00876
2332298	A_19_P00329303		lincRNA:chr4:127694975-127709825 reverse strand		-0.33198	-2.14775	0.0014
2320732	A_33_P3396089	SLC38A6	Homo sapiens solute carrier family 38, member 6 (SLC38A6), tran:	NM_153811	-0.33215	-2.14857	2.03E-06
1141449	A_23_P132889	CRMP1	Homo sapiens collapsin response mediator protein 1 (CRMP1), tra	NM_001014809	-0.33355	-2.15553	4.59E-07
2329340	A_19_P00322975		lincRNA:chr20:37049312-37059741 forward strand		-0.33455	-2.16047	7.13E-06
2318030	A_19_P00802866		lincRNA:chr1:42389413-42495113 forward strand		-0.33457	-2.16056	0.00009
878851	A_23_P131208	NR4A2	Homo sapiens nuclear receptor subfamily 4, group A, member 2 (N	NM_006186	-0.33585	-2.16693	0.00335
878842	A_23_P124108	ITGAM	Homo sapiens integrin, alpha M (complement component 3 recept	NM_000632	-0.33597	-2.16757	1.26E-32
887147	A_23_P124252	CAMK1D	Homo sapiens calcium/calmodulin-dependent protein kinase ID (C	NM_020397	-0.33601	-2.16777	3.03E-06
2332182	A_19_P00808719		lincRNA:chr1:9226160-9226803 forward strand		-0.33623	-2.16886	0.00103
2325542	A_19_P00330262		lincRNA:chr5:171274195-171288320 reverse strand		-0.33626	-2.16902	0.00009
2329734	A_19_P00319175		lincRNA:chr5:126187125-126189758 forward strand		-0.33656	-2.17049	0.00072
2327567	A_19_P00316097		lincRNA:chr18:5245991-5246806 reverse strand		-0.33659	-2.17064	2.71E-06
2326802	A_33_P3262022		Unknown		-0.33674	-2.17139	1.91E-06
1164201	A_23_P56288	LENG8	Homo sapiens leukocyte receptor cluster (LRC) member 8 (LENG	NM_052925	-0.33675	-2.17146	1.10E-06
1157338	A_32_P142440	PCSK9	Homo sapiens proprotein convertase subtilisin/kexin type 9 (PCSI	NM_174936	-0.33681	-2.17177	0.00004
2322964	A_33_P3231262	LOC257396	Homo sapiens hypothetical LOC257396 (LOC257396), non-coding	NR_034107	-0.33751	-2.17526	0.0067
2324351	A_19_P00811084		lincRNA:chr2:200984605-201020245 forward strand		-0.33784	-2.17692	9.95E-07
1166471	A_23_P407206	CLN8	Homo sapiens ceroid-lipofuscinosis, neuronal 8 (epilepsy, progres	NM_018941	-0.33835	-2.17944	1.46E-11
1139034	A_32_P159334	RECQL	Homo sapiens RecQ protein-like (DNA helicase Q1-like) (RECQL)	NM_002907	-0.33842	-2.17983	4.25E-11
2315600	A_19_P00801307		lincRNA:chr13:87525274-87925249 reverse strand		-0.3393	-2.18423	0.00001
880833	A_24_P274814	TBXAS1	Homo sapiens thromboxane A synthase 1 (platelet) (TBXAS1), tra	NM_030984	-0.33976	-2.18656	3.92E-12
1146624	A_23_P145681	ACTL6B	Homo sapiens actin-like 6B (ACTL6B), mRNA [NM_016188]	NM_016188	-0.3407	-2.19127	7.28E-06
2332150	A_19_P00315571		lincRNA:chr18:2517269-2519259 reverse strand		-0.34092	-2.19238	0.00849
887527	A_24_P89891	TRAF1	Homo sapiens TNF receptor-associated factor 1 (TRAF1), transc	NM_005658	-0.34128	-2.19423	4.77E-08
2314425	A_33_P3360758		potassium voltage-gated channel, KQT-like subfamily, member 2 [Source:HGNC S		-0.34171	-2.19638	2.35E-06
1150043	A_24_P205036	ZNF676	Homo sapiens zinc finger protein 676 (ZNF676), mRNA [NM_0010C	NM_001001411	-0.34204	-2.19804	1.33E-06
1161151	A_23_P159893	CHRDL1	Homo sapiens chordin-like 1 (CHRDL1), transcript variant 3, mRN	NM_145234	-0.34208	-2.19824	0.00318
2325362	A_33_P3371318		Homo sapiens cDNA clone IMAGE:4358137, with apparent retaine	BC066548	-0.34228	-2.19927	0.00141
2321597	A_33_P3366412		Unknown		-0.34383	-2.20713	0.00078
2329251	A_33_P3319791	NR4A1	Homo sapiens nuclear receptor subfamily 4, group A, member 1 (N	NM_002135	-0.34413	-2.20865	4.20E-09
2331481	A_19_P00329574		lincRNA:chr5:1544500-1567142 reverse strand		-0.34516	-2.2139	6.17E-07
2336090	A_19_P00328880		lincRNA:chr11:117668490-117680359 reverse strand		-0.34518	-2.214	7.25E-06
2320078	A_33_P3380217		tubulin tyrosine ligase-like family, member 9 [Source:HGNC Symb	BC104025	-0.34587	-2.21755	0.00029
1159863	A_24_P323967	ZNF880	Homo sapiens zinc finger protein 880 (ZNF880), mRNA [NM_00114	NM_001145434	-0.34594	-2.2179	7.56E-07
2327886	A_19_P00325682		lincRNA:chr14:61529972-61553247 forward strand		-0.34638	-2.22012	5.49E-07
882336	A_23_P157404	AP1S1	Homo sapiens adaptor-related protein complex 1, sigma 1 subunit	NM_001283	-0.34648	-2.22064	1.41E-12
2315161	A_33_P3327270	TMED7-TICAM2	Homo sapiens TMED7-TICAM2 readthrough (TMED7-TICAM2), tr	NM_001164469	-0.34712	-2.22391	5.19E-07
1146523	A_23_P127013	TCF7L2	Homo sapiens transcription factor 7-like 2 (T-cell specific, HMG-I	NM_030756	-0.34756	-2.22616	6.77E-12
885298	A_24_P215804	CKLF	Homo sapiens chemokine-like factor (CKLF), transcript variant 1,	NM_016951	-0.34763	-2.22652	1.23E-12
2315334	A_19_P00800270		lincRNA:chr2:152627254-152653279 reverse strand		-0.34872	-2.23212	0.00662
2326969	A_33_P3329187	DNMT1	Homo sapiens DNA (cytosine-5-)-methyltransferase 1 (DNMT1), I	NM_001130823	-0.34882	-2.23264	1.04E-12
869932	A_23_P214080	EGR1	Homo sapiens early growth response 1 (EGR1), mRNA [NM_00196	NM_001964	-0.35136	-2.24573	8.70E-13
1162429	A_23_P34233	QPRT	Homo sapiens quinolinate phosphoribosyltransferase (QPRT), mRI	NM_014298	-0.35258	-2.25206	0.00004
2329830	A_19_P00802433		lincRNA:chr6:53480866-53497141 forward strand		-0.35472	-2.26317	0.0004
1161430	A_23_P370479	POFUT1	Homo sapiens protein O-fucosyltransferase 1 (POFUT1), transcri	NM_015352	-0.35475	-2.26332	5.61E-07
1163490	A_24_P161813	LOC441666	Homo sapiens zinc finger protein 91 pseudogene (LOC441666), no	NR_024380	-0.3554	-2.26672	3.34E-07
2334317	A_19_P00807833		lincRNA:chr7:45983575-46089425 forward strand		-0.35691	-2.27462	0.00002
2314616	A_19_P00809136		lincRNA:chr21:36977805-37010580 reverse strand		-0.35753	-2.27789	5.50E-06
2335485	A_19_P00318845		lincRNA:chr10:4284540-4284771 reverse strand		-0.3578	-2.27928	0.00204
1143195	A_23_P353514	MSL3	Homo sapiens male-specific lethal 3 homolog (Drosophila) (MSL3)	NM_078628	-0.35848	-2.28287	1.37E-06
2327419	A_19_P00319269		lincRNA:chr18:37321093-37380224 reverse strand		-0.35986	-2.29014	0.0042
2330431	A_33_P3305250		proteasome (prosome, macropain) 26S subunit, non-ATPase, 5 [Source:HGNC Syn		-0.3601	-2.29137	0.00183
2335789	A_33_P3713035	LOC221814	Homo sapiens mRNA; cDNA DKFZp564C0371 (from clone DKFZp	AL122087	-0.36029	-2.29242	0.00025
2317540	A_19_P00325785		lincRNA:chr3:162922581-162942606 reverse strand		-0.36116	-2.29698	0.00072
2331396	A_19_P00332459		lincRNA:chr4:24517527-24528051 reverse strand		-0.36121	-2.29727	0.00001
2326344	A_33_P3357431		Novel protein (FLJ40547) [Source:UniProtKB/TrEMBL;Acc:Q5VZ	XR_112173	-0.36479	-2.3163	0.00092
887603	A_23_P44849	KCTD13	Homo sapiens potassium channel tetramerisation domain containi	NM_178863	-0.36513	-2.31809	0.00014
2320500	A_33_P3305438		Unknown		-0.36599	-2.32267	2.19E-07
1168278	A_23_P23966	ZNF488	Homo sapiens zinc finger protein 488 (ZNF488), mRNA [NM_15303	NM_153034	-0.36704	-2.32829	0.00149

Supplementary Table 1. Continued.

2318904	A_33_P3417459	SCARNA9L	Homo sapiens small Cajal body-specific RNA 9-like (retrotranspos	NR_023358	-0.3678	-2.3324	1.55E-07
2322027	A_33_P3265415		Unknown		-0.369	-2.33882	1.41E-07
2327883	A_19_P00813006		lincRNA:chr4:53578624-53580302 reverse strand		-0.36914	-2.33956	6.42E-07
1163109	A_32_P125832	LOC100128893	PREDICTED: Homo sapiens hypothetical LOC100128893 (LOC10C	XR_109470	-0.36919	-2.33984	0.00383
1160021	A_23_P309950		family with sequence similarity 178, member A [Source:HGNC Syn	AL833126	-0.36927	-2.34027	0.00318
1143819	A_23_P41304	GYPE	Homo sapiens glycophorin E (MNS blood group) (GYPE), transcript	NM_002102	-0.36932	-2.34054	0.00089
1145693	A_24_P6428	C20orf177	Homo sapiens chromosome 20 open reading frame 177 (C20orf17;	NM_022106	-0.37038	-2.34629	0.00048
2316705	A_19_P00331004		lincRNA:chr2:8001249-8036724 reverse strand		-0.37089	-2.34904	0.00003
883591	A_23_P50039	MC5R	Homo sapiens melanocortin 5 receptor (MC5R), mRNA [NM_00591	NM_005913	-0.37217	-2.35597	0.00725
2320503	A_19_P00325227		lincRNA:chr1:234908002-234918952 forward strand		-0.37238	-2.3571	1.16E-07
885303	A_23_P82651	NPTX2	Homo sapiens neuronal pentraxin II (NPTX2), mRNA [NM_002523]	NM_002523	-0.37444	-2.36829	0.00636
1153129	A_24_P357169	EPPK1	Homo sapiens epiplakin 1 (EPPK1), mRNA [NM_031308]	NM_031308	-0.37482	-2.37042	0.00121
2314514	A_33_P3323059		Homo sapiens cDNA, FLJ97323. [AK307375]	AK307375	-0.37539	-2.3735	0.0059
2336336	A_33_P3224730		hemicentin 2 [Source:HGNC Symbol;Acc:21293] [ENST000003024	AK128680	-0.37901	-2.39335	6.38E-06
2323387	A_19_P00319756		lincRNA:chr16:54953016-54959126 forward strand		-0.3795	-2.39606	0.00921
2330857	A_33_P3274754	LOC100131434	Homo sapiens hypothetical LOC100131434 (LOC100131434), non-	NR_027455	-0.38084	-2.40238	0.00037
1140655	A_24_P402415	SYT14	Homo sapiens synaptotagmin XIV (SYT14), transcript variant 4, ml	NM_153262	-0.38123	-2.40564	0.00492
2318701	A_33_P3213832		PREDICTED: Homo sapiens hypothetical LOC100130882 (LOC10C	XM_001715346	-0.38163	-2.40785	0.0077
2312755	A_33_P3359183	AWAT2	Homo sapiens acyl-CoA wax alcohol acyltransferase 2 (AWAT2), r	NM_001002254	-0.38704	-2.43805	0.0073
2329693	A_19_P00329395		lincRNA:chr6:56922016-56934541 reverse strand		-0.38762	-2.44131	0.00016
1145990	A_32_P180741	TNK2	Homo sapiens tyrosine kinase, non-receptor, 2 (TNK2), transcript	NM_001010938	-0.38787	-2.44269	0.00059
2319028	A_19_P00806762		lincRNA:chrX:73043635-73044133 forward strand		-0.38787	-2.44271	0.00194
2331530	A_19_P00324780		lincRNA:chr6:151713007-151720482 forward strand		-0.38899	-2.44901	5.07E-08
2335618	A_33_P3285945	CCL21	Homo sapiens chemokine (C-C motif) ligand 21 (CCL21), mRNA [I	NM_002989	-0.3892	-2.45019	0.00006
2326442	A_33_P3383816	LOC400657	Homo sapiens hypothetical LOC400657 (LOC400657), non-coding	NR_024484	-0.38987	-2.45395	0.0009
2326907	A_33_P3267482	KIAA1804	Homo sapiens mixed lineage kinase 4 (KIAA1804), mRNA [NM_032	NM_032435	-0.39131	-2.46212	3.76E-08
2329628	A_33_P3813818	LOC100505510	PREDICTED: Homo sapiens hypothetical protein LOC100505510 (	XM_003118976	-0.39253	-2.46904	0.00325
2328715	A_33_P3369731		Unknown		-0.39403	-2.4776	1.34E-06
2335508	A_19_P00322909		lincRNA:chr18:53709435-53763540 reverse strand		-0.39492	-2.48269	0.00001
1156882	A_23_P307502	C9orf53	Homo sapiens chromosome 9 open reading frame 53 (C9orf53), no	NR_024274	-0.3952	-2.48426	0.0029
2328855	A_33_P3210228		zinc finger, CCHC domain containing 6 [Source:HGNC Symbol;Acc:25817] [ENSTO(		-0.39536	-2.4852	0.00542
2335507	A_19_P00810151		lincRNA:chr2:134879255-134978205 forward strand		-0.39538	-2.4853	0.00342
1146316	A_24_P522631	TMEM201	Homo sapiens transmembrane protein 201 (TMEM201), transcript	NM_001130924	-0.39548	-2.48587	2.93E-08
2329326	A_33_P3210349		cDNA FLJ44904 fis, clone BRAMY3005656 [Source:UniProtKB/T	XR_111612	-0.39561	-2.48664	0.00039
2316242	A_33_P3349384		Unknown		-0.39657	-2.49213	6.46E-07
2327596	A_33_P3220857		UPF0553 protein C9orf64 [Source:UniProtKB/Swiss-Prot;Acc:Q5T6V5] [ENSTO(		-0.39774	-2.49886	0.0007
1159779	A_32_P85042	LOC100132815	HCG2000535cDNA FLJ32177 fis, clone PLACE6001294 ; [Source:	AK056856	-0.39779	-2.49916	0.00001
2316880	A_19_P00807856		lincRNA:chr15:79676505-79677063 forward strand		-0.39942	-2.50851	0.00258
1144810	A_32_P232035	LOC100270746	Homo sapiens hypothetical LOC100270746 (LOC100270746), non-	NR_026776	-0.39966	-2.50993	5.54E-08
2330341	A_19_P00329166		lincRNA:chrX:107264119-107280819 forward strand		-0.39971	-2.51018	4.12E-08
2315468	A_33_P3418576		serine active site containing 1 [Source:HGNC Symbol;Acc:21061]	BC048335	-0.4016	-2.52114	0.00294
2331809	A_33_P3214052		Homo sapiens cDNA clone IMAGE:5266307. [BC035182]	BC035182	-0.40167	-2.52155	0.00001
1148676	A_24_P163237	STOX2	Homo sapiens storkhead box 2 (STOX2), mRNA [NM_020225]	NM_020225	-0.40211	-2.52413	0.0005
2322749	A_33_P3383626	ZSCAN5D	PREDICTED: Homo sapiens zinc finger and SCAN domain containi	XM_001725568	-0.40265	-2.52724	0.00079
2334192	A_33_P3213712		Unknown		-0.40387	-2.53438	0.00006
2327189	A_33_P3297651	LOC100130540	Homo sapiens cDNA FLJ43078 fis, clone BRTHA3016845. [AK125	AK125068	-0.40389	-2.5345	0.00146
2318457	A_33_P3273000	PTK2B	Homo sapiens cDNA FLJ46514 fis, clone THYMU3032798, highly	AK128371	-0.4039	-2.53456	1.78E-08
2327360	A_33_P3270295	FRS3	Homo sapiens fibroblast growth factor receptor substrate 3 (FRS:	NM_006653	-0.40517	-2.54196	1.61E-11
886880	A_24_P50245	HLA-DMA	Homo sapiens major histocompatibility complex, class II, DM alpha	NM_006120	-0.40764	-2.55644	0.00028
2328042	A_33_P3299872	HINT3	Homo sapiens histidine triad nucleotide binding protein 3 (HINT3),	NM_138571	-0.4083	-2.56034	1.38E-08
2328930	A_19_P00808319		lincRNA:chr12:68383224-68415107 forward strand		-0.40849	-2.56146	0.00356
1150263	A_24_P67585	VGLL3	Homo sapiens vestigial like 3 (Drosophila) (VGLL3), mRNA [NM_01	NM_016206	-0.41185	-2.58136	1.21E-08
2335532	A_19_P00323968		lincRNA:chr19:22019985-22034034 forward strand		-0.41309	-2.58874	0.00445
2329029	A_33_P3300635	PFKFB2	Homo sapiens 6-phosphofructo-2-kinase/fructose-2,6-biphospha	NM_006212	-0.41322	-2.58954	5.99E-16
880525	A_23_P366376	TDGF1	Homo sapiens teratocarcinoma-derived growth factor 1 (TDGF1),	NM_003212	-0.41399	-2.59412	0.00065
1142265	A_24_P704878		Unknown		-0.41571	-2.60439	0.00662
2330210	A_33_P3229472	KRTAP5-10	Homo sapiens keratin associated protein 5-10 (KRTAP5-10), mRI	NM_001012710	-0.41593	-2.60571	0.00763
2332458	A_33_P3221960	IL18RAP	Homo sapiens interleukin 18 receptor accessory protein (IL18RAF	NM_003853	-0.41636	-2.60833	8.97E-09
2318939	A_19_P00812662		lincRNA:chr12:5377839-5428564 reverse strand		-0.41894	-2.62386	2.51E-08
2327171	A_33_P3251871		Unknown		-0.41912	-2.62492	1.19E-08
2323389	A_33_P3320533		full-length cDNA clone CS0DJ015YB09 of T cells (Jurkat cell line	CR594845	-0.41983	-2.62925	0.00405
2322314	A_33_P3797403	LOC283387	Homo sapiens hypothetical protein LOC283387, mRNA (cDNA clo	BC032840	-0.42037	-2.63248	0.00308
1140270	A_23_P13294	OR1S2	Homo sapiens olfactory receptor, family 1, subfamily S, member 2	NM_001004459	-0.42161	-2.64002	0.001
1139721	A_24_P187614	LOC100129648	Homo sapiens cDNA FLJ40318 fis, clone TESTI2030556. [AK0976	AK097637	-0.42292	-2.64801	0.00366
2319651	A_33_P3277883	LOC100129931	Homo sapiens hypothetical LOC100129931 (LOC100129931), non-	NR_033828	-0.42348	-2.65141	0.00053
1154881	A_23_P403284	OTX1	Homo sapiens orthodenticle homeobox 1 (OTX1), transcript varian	NM_014562	-0.42478	-2.6594	0.00036
2316725	A_33_P3329712		Unknown		-0.43016	-2.6925	0.00108
2313209	A_19_P00804779		lincRNA:chr4:47840518-47846712 forward strand		-0.43104	-2.69801	0.0002
1156906	A_23_P69970	SOX30	Homo sapiens SRY (sex determining region Y)-box 30 (SOX30), tr	NM_178424	-0.4316	-2.70148	0.00017
1161697	A_24_P358868	ZNF728	PREDICTED: Homo sapiens zinc finger protein 728 (ZNF728), mRI	XM_001720936	-0.43329	-2.71201	3.60E-09
2331653	A_19_P00328318		lincRNA:chr2:42562846-42574346 forward strand		-0.43405	-2.71673	0.00001
1140043	A_23_P416314	HRASLS5	Homo sapiens HRAS-like suppressor family, member 5 (HRASLS5	NM_054108	-0.43644	-2.73177	2.63E-09

Supplementary Table 1. Continued.

2319478	A_19_P00802496		lincRNA:chr8:16347304-16355399 reverse strand		-0.43718	-2.73639	4.25E-09
2324838	A_19_P00806973		lincRNA:chr2:37325696-37331035 forward strand		-0.43895	-2.74757	0.00403
1159879	A_23_P357966	PCMTD1	Homo sapiens protein-L-isoaspartate (D-aspartate) O-methyltrar	NM_052937	-0.43899	-2.74782	3.35E-06
2321374	A_33_P3267562	VAX2	Homo sapiens ventral anterior homeobox 2 (VAX2), mRNA [NM_01	NM_012476	-0.44038	-2.75663	2.15E-09
884452	A_23_P348028	IL1F6	Homo sapiens interleukin 1 family, member 6 (epsilon) (IL1F6), mR	NM_014440	-0.44068	-2.75854	0.00943
2336353	A_33_P3629247	ANKMY1	Homo sapiens ankyrin repeat and MYND domain containing 1 (ANI	NM_017844	-0.44345	-2.77623	2.14E-09
2335098	A_19_P00812874		lincRNA:chr14:91315547-91326047 reverse strand		-0.44668	-2.79689	5.05E-06
2316535	A_33_P3264444		Prefoldin subunit 6 (Protein Ke2) [Source:UniProtKB/Swiss-Prot;,	CR609151	-0.44962	-2.8159	1.25E-09
1167425	A_24_P717262	LOC100131479	PREDICTED: Homo sapiens zinc finger protein 430-like (LOC1001	XM_001715121	-0.45234	-2.83361	0.00001
2325843	A_19_P00319889		lincRNA:chr2:224569352-224588757 reverse strand		-0.45366	-2.84227	0.00546
2333082	A_19_P00802597		lincRNA:chr11:18610699-18619599 reverse strand		-0.45442	-2.84719	0.00484
2330488	A_33_P3418790	GNRH2	gonadotropin-releasing hormone 2 [Source:HGNC Symbol;Acc:4420] [ENST00003		-0.45637	-2.86004	0.00444
2321044	A_19_P00325965		lincRNA:chr16:54944999-54959642 reverse strand		-0.45769	-2.8687	8.85E-08
2315882	A_33_P3295096	NAG20	PREDICTED: Homo sapiens NAG20 (NAG20), miscRNA [XR_10882	XR_108823	-0.46094	-2.89025	0.00486
2315879	A_33_P3286923	TMPRSS5	Homo sapiens transmembrane protease, serine 5 (TMPRSS5), mR	NM_030770	-0.46127	-2.89246	0.00643
881987	A_24_P365679		full-length cDNA clone CS0DJ012YO23 of T cells (Jurkat cell line	CR622757	-0.46226	-2.89907	0.00518
2319423	A_33_P3249529	PCNX	Homo sapiens pecanex homolog (Drosophila) (PCNX), mRNA [NM_	NM_014982	-0.46236	-2.89974	5.89E-10
1150618	A_23_P252276	ARHGEF38	Homo sapiens Rho guanine nucleotide exchange factor (GEF) 38 (	NM_017700	-0.46256	-2.9011	0.00038
882554	A_23_P149613	FMO1	Homo sapiens flavin containing monooxygenase 1 (FMO1), mRNA	NM_002021	-0.46387	-2.90985	4.81E-10
1146573	A_23_P390443	SHISA7	Homo sapiens shisa homolog 7 (Xenopus laevis) (SHISA7), mRNA	NM_001145176	-0.46983	-2.95006	0.00245
882483	A_24_P160380	PDLIM2	Homo sapiens PDZ and LIM domain 2 (mystique) (PDLIM2), transc	NM_176871	-0.47024	-2.95283	0.00004
2314346	A_33_P3351735	CCDC87	Homo sapiens coiled-coil domain containing 87 (CCDC87), mRNA	NM_018219	-0.47198	-2.9647	0.00195
2334462	A_33_P3231086		Unknown		-0.47307	-2.97217	2.90E-08
2327176	A_33_P3232173	PSPC1	paraspeckle component 1 [Source:HGNC Symbol;Acc:20320] [ENS	BE147842	-0.4746	-2.9826	1.23E-09
2313969	A_19_P00324769		lincRNA:chrX:113008710-113015135 reverse strand		-0.47627	-2.99411	0.00914
2315946	A_19_P00800214		lincRNA:chr14:71148522-71171347 reverse strand		-0.47658	-2.99628	0.0003
882976	A_32_P130630	SLC16A7	Homo sapiens solute carrier family 16, member 7 (monocarboxylic	NM_004731	-0.47792	-3.00556	1.87E-13
884239	A_24_P47681	CAND1	Homo sapiens cullin-associated and neddylation-dissociated 1 (C/	NM_018448	-0.48062	-3.02427	2.58E-19
2328177	A_33_P3336720	HAMP	Homo sapiens hepcidin antimicrobial peptide (HAMP), mRNA [NM_	NM_021175	-0.48171	-3.03187	3.03E-19
1159870	A_23_P83579	ARNT2	Homo sapiens aryl-hydrocarbon receptor nuclear translocator 2 (/	NM_014862	-0.48216	-3.03503	8.03E-15
2335200	A_19_P00812263		lincRNA:chr7:112747264-112798839 reverse strand		-0.48264	-3.03833	0.00374
1159504	A_32_P216734	SPDYE3	Homo sapiens speedy homolog E3 (Xenopus laevis) (SPDYE3), mF	NM_001004351	-0.48489	-3.05417	0.00394
1141478	A_24_P91701	C22orf43	Homo sapiens chromosome 22 open reading frame 43 (C22orf43),	NM_016449	-0.48563	-3.05939	1.60E-10
1145439	A_23_P50232	AURKC	Homo sapiens aurora kinase C (AURKC), transcript variant 1, mRI	NM_001015878	-0.48697	-3.06881	4.25E-09
2312852	A_33_P3410232	LY6G6E	Homo sapiens lymphocyte antigen 6 complex, locus G6E (LY6G6E	NR_024541	-0.48702	-3.06918	0.00769
1143171	A_24_P175187	SAMD9	Homo sapiens sterile alpha motif domain containing 9 (SAMD9), tr.	NM_017654	-0.48717	-3.07019	3.71E-06
2330830	A_19_P00812238		lincRNA:chr7:106416539-106423571 reverse strand		-0.48724	-3.07074	0.00749
1148028	A_23_P155463	LRRC2	Homo sapiens leucine rich repeat containing 2 (LRRC2), mRNA [N	NM_024512	-0.48728	-3.071	8.04E-10
1161777	A_32_P831725	C3orf62	Homo sapiens chromosome 3 open reading frame 62 (C3orf62), ml	NM_198562	-0.48868	-3.08091	0.00019
2328147	A_19_P00317628		lincRNA:chr7:96279734-96293576 reverse strand		-0.48945	-3.08639	9.90E-06
1161571	A_23_P132760	TRH	Homo sapiens thyrotropin-releasing hormone (TRH), mRNA [NM_0	NM_007117	-0.49005	-3.09064	0.00002
2323335	A_19_P00322448		lincRNA:chr6:167317185-167318297 reverse strand		-0.49058	-3.09444	0.0002
2314330	A_19_P00330523		lincRNA:chr8:133071643-133092468 reverse strand		-0.49405	-3.11926	0.00013
1161094	A_23_P325887	TBC1D8B	Homo sapiens TBC1 domain family, member 8B (with GRAM doma	NM_017752	-0.49467	-3.12369	0.00002
2336271	A_33_P3357516	SLC10A7	Homo sapiens solute carrier family 10 (sodium/bile acid cotranspc	NM_032128	-0.49563	-3.13063	0.00061
1154628	A_23_P387537	PRRG3	Homo sapiens proline rich Gla (G-carboxyglutamic acid) 3 (transm	NM_024082	-0.4987	-3.15281	0.004
2318529	A_33_P3385516	CBLN4	Homo sapiens cerebellin 4 precursor (CBLN4), mRNA [NM_080617	NM_080617	-0.49995	-3.16194	5.22E-06
2332950	A_33_P3379046	LOC729770	PREDICTED: Homo sapiens hypothetical LOC729770 (LOC72977C	XR_112442	-0.50106	-3.17	6.79E-11
2322541	A_33_P3244224	LOC100129048	Homo sapiens cDNA FLJ46195 fis, clone TESTI4006539. [AK1280	AK128074	-0.50344	-3.18744	0.00017
1151113	A_32_P70203	FAM41C	Homo sapiens family with sequence similarity 41, member C (FAM	NR_027055	-0.50364	-3.18886	0.00953
2329498	A_33_P3273267	LOC729863	PREDICTED: Homo sapiens putative zinc finger protein ENSP000(	XM_003120379	-0.50625	-3.20811	1.93E-07
2330683	A_19_P00329806		lincRNA:chr12:54356095-54362515 reverse strand		-0.50667	-3.2112	4.89E-11
1165888	A_32_P315395	MGC16275	Homo sapiens hypothetical protein MGC16275 (MGC16275), non-i	NR_026914	-0.50747	-3.21712	0.00356
2333356	A_33_P3268686	FBXW12	Homo sapiens F-box and WD repeat domain containing 12 (FBXW	NM_207102	-0.50957	-3.23276	0.00022
2322857	A_19_P00802151		lincRNA:chr3:156894106-156971481 reverse strand		-0.5098	-3.23442	6.22E-08
1154119	A_24_P102650	MUC5B	Homo sapiens mucin 5B, oligomeric mucus/gel-forming (MUC5B),	NM_002458	-0.5128	-3.25685	0.00393
2328568	A_33_P3250895	PSMB11	Homo sapiens proteasome (prosome, macropain) subunit, beta typ	NM_001099780	-0.51906	-3.30418	0.00344
2329619	A_33_P3323989		Unknown		-0.52059	-3.31582	0.0005
2317251	A_33_P3771741		Homo sapiens cDNA FLJ34909 fis, clone NT2RI2009301, moderat	AK092228	-0.52177	-3.32481	0.0028
2322528	A_19_P00319043		lincRNA:chr8:52808516-52808934 forward strand		-0.52301	-3.33434	0.00775
2333677	A_33_P3221009		Homo sapiens cDNA FLJ45966 fis, clone PLACE7015647. [AK127	AK127863	-0.52374	-3.33994	1.48E-07
2335603	A_19_P00806053		lincRNA:chr8:6516692-6533242 reverse strand		-0.52923	-3.38247	0.00754
2333636	A_33_P3388067	ZNF735	Homo sapiens zinc finger protein 735 (ZNF735), mRNA [NM_00115	NM_001159524	-0.52984	-3.38564	1.08E-06
2325543	A_33_P3387320	RPL31	full-length cDNA clone CS0DI015YG06 of Placenta Cot 25-norma	CR595074	-0.53259	-3.40869	0.00282
2330608	A_33_P3231252	NHLH2	Homo sapiens nescient helix loop helix 2 (NHLH2), transcript varia	NM_005599	-0.53442	-3.42313	1.26E-11
1146199	A_32_P225659	UTS2D	Homo sapiens urotensin 2 domain containing (UTS2D), mRNA [NM	NM_198152	-0.53724	-3.44542	2.18E-06
869406	A_23_P133408	CSF2	Homo sapiens colony stimulating factor 2 (granulocyte-macrophag	NM_000758	-0.5396	-3.46419	0.00713
2332554	A_19_P00324198		lincRNA:chr2:74610317-74618792 forward strand		-0.54157	-3.47995	0.00019
881873	A_23_P319583	RIMS3	Homo sapiens regulating synaptic membrane exocytosis 3 (RIMS3	NM_014747	-0.54251	-3.48747	0.00144
2313679	A_19_P00321221		lincRNA:chr5:25413547-25446034 reverse strand		-0.54542	-3.51088	0.00812
882121	A_23_P22761	SHOX	Homo sapiens short stature homeobox (SHOX), transcript variant	NM_006883	-0.54826	-3.53398	1.18E-09
1145663	A_23_P209700	NMUR1	Homo sapiens neuromedin U receptor 1 (NMUR1), mRNA [NM_006	NM_006056	-0.54919	-3.54155	2.70E-09

Supplementary Table 1. Continued.

2327340	A_33_P3281728	LOC388692	PREDICTED: Homo sapiens hypothetical LOC100133075 (LOC10C	XR_039086	-0.55051	-3.55233	0.00961
2319854	A_19_P00807924		lincRNA:chr4:103337452-103350952 forward strand		-0.55237	-3.56755	0.00006
2335869	A_33_P3403666	NDOR1	Homo sapiens NADPH dependent diflavin oxidoreductase 1 (NDOI	NM_001144026	-0.55822	-3.61591	0.00006
2320991	A_19_P00807986		lincRNA:chr9:123606154-123611279 reverse strand		-0.55843	-3.61767	0.0053
2327178	A_19_P00809587		lincRNA:chr8:96297524-96413824 reverse strand		-0.56038	-3.63392	5.74E-08
2335229	A_33_P3411384		Unknown		-0.56193	-3.64698	2.58E-12
2325276	A_19_P00325176		lincRNA:chr2:43102921-43110596 reverse strand		-0.5637	-3.66189	3.62E-12
2325528	A_33_P3382281		Unknown		-0.56478	-3.67093	0.00385
2331355	A_33_P3216694	HIVEP3	Homo sapiens human immunodeficiency virus type I enhancer binc	NM_024503	-0.56903	-3.70704	0.0027
1152611	A_23_P165239	ZNF208	Homo sapiens zinc finger protein 208 (ZNF208), mRNA [NM_00715	NM_007153	-0.57273	-3.73878	3.36E-11
2323294	A_33_P3420704		Unknown		-0.58178	-3.81752	0.00071
2320939	A_19_P00322282		lincRNA:chr3:185003349-185009534 reverse strand		-0.58273	-3.82586	0.00697
2319257	A_19_P00331179		lincRNA:chr15:42773808-42780958 forward strand		-0.5837	-3.83445	0.00512
2325170	A_33_P3278220		Rab9 effector protein with kelch motifs [Source:HGNC Symbol;Ac	AL832249	-0.58573	-3.85241	7.68E-13
1148030	A_23_P349398	ZAR1	Homo sapiens zygote arrest 1 (ZAR1), mRNA [NM_175619]	NM_175619	-0.58606	-3.85535	0.00857
2322787	A_33_P3213311		Unknown		-0.58715	-3.86498	3.51E-12
2320949	A_33_P3289701	OR2T12	Homo sapiens olfactory receptor, family 2, subfamily T, member 1;	NM_001004692	-0.58788	-3.87152	0.00107
2335604	A_33_P3230558	EML5	Homo sapiens echinoderm microtubule associated protein like 5 (E	NM_183387	-0.58843	-3.87639	8.77E-08
1142166	A_32_P206735		Unknown		-0.59521	-3.93739	0.0013
2334404	A_33_P3372094	LLGL1	Homo sapiens lethal giant larvae homolog 1 (Drosophila) (LLGL1),	NM_004140	-0.59566	-3.94152	0.0066
1162299	A_23_P79108	ATP8B3	Homo sapiens ATPase, aminophospholipid transporter, class I, typ	NM_138813	-0.59704	-3.95407	0.0009
2328169	A_19_P00804666		lincRNA:chr19:57769688-57777413 reverse strand		-0.60622	-4.03854	2.81E-13
2333954	A_33_P3226761	SOX18	Homo sapiens SRY (sex determining region Y)-box 18 (SOX18), m	NM_018419	-0.6081	-4.05604	6.74E-21
2322919	A_19_P00805860		lincRNA:chr1:9229531-9229931 reverse strand		-0.61013	-4.07504	6.71E-13
1159657	A_24_P626932	LOC100510579	PREDICTED: Homo sapiens hypothetical protein LOC100510579 (	XM_003120527	-0.61293	-4.10141	0.0029
2320579	A_33_P3235721	C11orf87	Homo sapiens chromosome 11 open reading frame 87 (C11orf87),	NM_207645	-0.62185	-4.18653	0.00164
2331566	A_33_P3570974		Homo sapiens chromosome 8 open reading frame 50, mRNA (cDN	BC043205	-0.62369	-4.20423	1.00E-06
1157213	A_23_P402331	WFDC5	Homo sapiens WAP four-disulfide core domain 5 (WFDC5), mRNA	NM_145652	-0.62622	-4.22881	0.00004
2329264	A_19_P00316615		lincRNA:chr1:234794725-234795953 reverse strand		-0.63139	-4.27948	0.00128
2321677	A_33_P3319276	FAM178B	Homo sapiens family with sequence similarity 178, member B (FAI	NM_001122646	-0.63217	-4.28718	0.00221
2323682	A_33_P3484806	FLJ21408	Homo sapiens cDNA: FLJ21408 fis, clone COL03917. [AK025061]	AK025061	-0.63232	-4.28864	3.13E-06
2330028	A_33_P3285799		adenylate kinase domain containing 1 [Source:HGNC Symbol;Acc:;	AK131244	-0.63272	-4.29257	0.00831
2329247	A_33_P3345126	ZNF568	Homo sapiens zinc finger protein 568 (ZNF568), mRNA [NM_19853	NM_198539	-0.63524	-4.31753	0.00954
2331609	A_19_P00326527		lincRNA:chr15:75201097-75208047 forward strand		-0.64889	-4.45548	4.06E-14
2316268	A_19_P00322295		lincRNA:chr8:90737074-90737855 forward strand		-0.64959	-4.46259	9.69E-12
2331703	A_19_P00809021		lincRNA:chr5:127385751-127405576 forward strand		-0.66278	-4.6002	0.00726
1138723	A_24_P211842	FADS6	Homo sapiens fatty acid desaturase domain family, member 6 (FAI	NM_178128	-0.66304	-4.60298	0.0018
2324909	A_19_P00326849		lincRNA:chr3:182651981-182660175 forward strand		-0.66375	-4.61055	0.00097
2327787	A_33_P3357097	LOC100132741	Homo sapiens hypothetical LOC100132741 (LOC100132741), non-	NR_034004	-0.66506	-4.62448	0.00018
886537	A_24_P151	KCNAB2	Homo sapiens potassium voltage-gated channel, shaker-related si	NM_003636	-0.67317	-4.71161	0.00009
2318600	A_33_P3209130	LOC100128496	CR738909 Soares_testis_NHT Homo sapiens cDNA clone IMAGp9	CR738909	-0.67401	-4.72075	0.00907
880654	A_24_P156769	MPL	Homo sapiens myeloproliferative leukemia virus oncogene (MPL), i	NM_005373	-0.67443	-4.72534	0.00065
2316633	A_33_P3694746		Homo sapiens cDNA FLJ32224 fis, clone PLACE6004336. [AK056	AK056786	-0.68716	-4.86583	0.00028
2332331	A_19_P00318912		lincRNA:chr2:8029554-8037881 forward strand		-0.69311	-4.93303	0.00104
2318513	A_33_P3544856	LOC100292680	Homo sapiens cDNA FLJ33297 fis, clone BNGH42001406. [AK090	AK090616	-0.69358	-4.93837	0.00207
2322152	A_19_P00801407		lincRNA:chr5:169624950-169625424 reverse strand		-0.69375	-4.94024	0.00082
2322825	A_33_P3394252	C2orf67	Homo sapiens chromosome 2 open reading frame 67 (C2orf67), ml	NM_152519	-0.69879	-4.99793	0.00885
2335021	A_19_P00809030		lincRNA:chr2:156877046-157111432 reverse strand		-0.69946	-5.0057	0.00109
1149764	A_23_P127663	PRRG4	Homo sapiens proline rich Gla (G-carboxyglutamic acid) 4 (transm	NM_024081	-0.70201	-5.03509	0.00823
2316577	A_33_P3297630	LOC729966	Homo sapiens hypothetical LOC729966 (LOC729966), non-coding	NR_036575	-0.708	-5.10502	0.00794
2334561	A_33_P3301097		Unknown		-0.71103	-5.14085	7.37E-15
2324976	A_19_P00808521		lincRNA:chr1:247365199-247365322 forward strand		-0.71235	-5.15646	0.00802
2319510	A_33_P3655646	LOC100130354	Homo sapiens hypothetical protein LOC100130354, mRNA (cDNA	BC069735	-0.71256	-5.15895	0.00865
2314874	A_33_P3408514	SCNN1D	Homo sapiens sodium channel, nonvoltage-gated 1, delta (SCNN1I	NM_001130413	-0.71703	-5.21236	0.00004
2327353	A_33_P3326667		Unknown		-0.71994	-5.2474	0.00397
2332442	A_33_P3212072	GORAB	Homo sapiens golgin, RAB6-interacting (GORAB), transcript varial	NM_001146039	-0.72089	-5.2589	0.00577
2319706	A_19_P00319435		lincRNA:chr5:42985502-43010278 forward strand		-0.72783	-5.34355	0.00003
2325467	A_33_P3355937	LOC284276	Homo sapiens hypothetical LOC284276 (LOC284276), non-coding	NR_015417	-0.73337	-5.41215	0.00414
2318966	A_33_P3358815	DEFB103A	Homo sapiens defensin, beta 103A (DEFB103A), mRNA [NM_0010I	NM_001081551	-0.73746	-5.46339	0.00396
1158286	A_23_P144746	ZNF454	Homo sapiens zinc finger protein 454 (ZNF454), transcript variant	NM_182594	-0.73768	-5.46615	0.00402
2327859	A_19_P00800915		lincRNA:chr2:156244304-156300229 reverse strand		-0.74204	-5.52131	0.00336
2333532	A_33_P3416644		Unknown		-0.74391	-5.54506	0.00361
2327991	A_19_P00806227		lincRNA:chr5:151189307-151195107 reverse strand		-0.75106	-5.63717	0.0027
1168250	A_24_P940086	NXPH3	Homo sapiens neurexophilin 3 (NXPH3), mRNA [NM_007225]	NM_007225	-0.75369	-5.67145	0.00281
2319996	A_33_P3413808	PABPC1L	Homo sapiens poly(A) binding protein, cytoplasmic 1-like (PABPC	NM_001124756	-0.77001	-5.88861	0.00221
2325738	A_33_P3414494		Unknown		-0.7768	-5.98134	2.15E-06
2324715	A_33_P3402211	LOC100506220	PREDICTED: Homo sapiens hypothetical LOC100506220 (LOC10C	XR_110895	-0.77875	-6.00823	0.00142
2320789	A_19_P00318614		lincRNA:chr3:63110000-63110663 forward strand		-0.78144	-6.04561	0.00173
2324039	A_19_P00805400		lincRNA:chr1:211564402-211589752 reverse strand		-0.78789	-6.136	1.91E-06
1144183	A_23_P131588	BMP10	Homo sapiens bone morphogenetic protein 10 (BMP10), mRNA [N	NM_014482	-0.78819	-6.14027	3.30E-08
2335486	A_33_P3280521	MFAP3L	Homo sapiens microfibrillar-associated protein 3-like (MFAP3L), t	NM_021647	-0.80384	-6.36556	0.00076
2334108	A_19_P00812045		lincRNA:chr4:129215150-129232550 forward strand		-0.81184	-6.48401	0.00061

Supplementary Table 1. Continued.

2327609	A_33_P3408137	LOC100130248	Homo sapiens cDNA FLJ42222 fis, clone THYMU2039411. [AK124	AK124216	−0.81343	−6.50775	0.0005
881671	A_23_P325606	ESX1	Homo sapiens ESX homeobox 1 (ESX1), mRNA [NM_153448]	NM_153448	−0.82135	−6.62752	1.41E−13
2326429	A_19_P00328380		lincRNA:chr3:175987356−176039381 reverse strand		−0.82722	−6.71762	4.67E−17
2320639	A_19_P00325058		lincRNA:chr14:101389472−101539847 reverse strand		−0.83251	−6.80009	0.00034
2327579	A_33_P3320212		Unknown		−0.84105	−6.93511	0.00021
2320045	A_33_P3379081		601191189F1 NIH_MGC_7 Homo sapiens cDNA clone IMAGE:3535(	BE265920	−0.8414	−6.94072	0.00022
869436	A_23_P106194	FOS	Homo sapiens FBJ murine osteosarcoma viral oncogene homolog	NM_005252	−0.84277	−6.96253	2.36E−32
2324247	A_33_P3264524		Unknown		−0.85296	−7.12795	9.90E−09
1152443	A_24_P15877		Homo sapiens FKSG41 (FKSG41) mRNA, complete cds. [AF33376	AF333762	−0.86157	−7.27055	0.00014
1158569	A_23_P152330	DOC2A	Homo sapiens double C2-like domains, alpha (DOC2A), mRNA [NN	NM_003586	−0.86457	−7.321	1.10E−07
1167892	A_23_P136571	C1orf14	Homo sapiens chromosome 1 open reading frame 14 (C1orf14), ml	NM_030933	−0.86562	−7.33865	0.00011
2314250	A_33_P3389168		Unknown		−0.92293	−8.37387	0.00001
878987	A_23_P64661	ARHGAP9	Homo sapiens Rho GTPase activating protein 9 (ARHGAP9), trans	NM_032496	−0.92986	−8.50869	8.11E−09
1155346	A_24_P59236	RESP18	Homo sapiens regulated endocrine-specific protein 18 homolog (ra	NM_001007089	−0.9385	−8.67957	2.10E−06
2333913	A_33_P3400302	TTTY18	Homo sapiens testis-specific transcript, Y-linked 18 (non-protein	NR_001550	−0.94197	−8.74916	0.0016
2322698	A_33_P3311994	KIAA1239	Homo sapiens KIAA1239 (KIAA1239), mRNA [NM_001144990]	NM_001144990	−0.94575	−8.82563	4.13E−06
2334705	A_33_P3230439	LOC100130148	Homo sapiens hypothetical LOC100130148 (LOC100130148), non-	NR_024560	−0.96351	−9.19422	1.07E−06
2334680	A_19_P00806038		lincRNA:chr8:125471969−125486269 reverse strand		−1.01827	−10.42959	2.45E−19
2313467	A_33_P3280346	LOC100130857	Homo sapiens cDNA FLJ39731 fis, clone SMINT2015745. [AK097(	AK097050	−1.05551	−11.36357	2.45E−08
1142788	A_32_P135517	LOC100506105	PREDICTED: Homo sapiens hypothetical LOC100506105 (LOC10C	XR_109318	−1.0714	−11.78697	1.21E−15
2334587	A_33_P3350744		Unknown		−1.13207	−13.55419	3.10E−12
2330300	A_33_P3809497	FLJ37505	Homo sapiens hypothetical LOC400087 (FLJ37505), non-coding F	NR_033987	−1.17876	−15.0925	7.23E−19
2314863	A_33_P3337569	C4orf40	Homo sapiens chromosome 4 open reading frame 40 (C4orf40), ml	NM_214711	−1.18137	−15.18356	1.74E−11
2326840	A_19_P00801412		lincRNA:chr21:10701104−10873604 forward strand		−1.19385	−15.62617	5.73E−12
2334781	A_33_P3358277		potassium channel, subfamily K, member 1 [Source:HGNC Symbol:	AK090902	−1.21173	−16.28278	2.36E−12
2327788	A_33_P3300747	ADHFE1	Homo sapiens alcohol dehydrogenase, iron containing, 1 (ADHFE1)	NM_144650	−1.28046	−19.07485	3.76E−19
2316576	A_19_P00806426		lincRNA:chr14:64116297−64131817 forward strand		−1.2808	−19.08983	5.06E−14
2333644	A_33_P3283974		PREDICTED: Homo sapiens hypothetical LOC399829 (LOC39982S	XR_109033	−1.37034	−23.46043	3.37E−16
2334403	A_33_P3322348	LOC100128942	cDNA FLJ46600 fis, clone THYMU3047144 [Source:UniProtKB/T	AK128457	−1.5982	−39.64566	5.24E−20
2334194	A_19_P00802785		lincRNA:chr3:72644685−72734460 reverse strand		−1.64633	−44.2923	1.49E−20
2334273	A_33_P3601802		AGENCOURT_13978263 NIH_MGC_172 Homo sapiens cDNA 5', mf	CD049182	−1.71019	−51.30877	3.73E−21
1150505	A_23_P155539	POU1F1	Homo sapiens POU class 1 homeobox 1 (POU1F1), transcript vari	NM_000306	−1.85572	−71.73266	8.83E−23
2334402	A_19_P00805097		lincRNA:chr5:88390860−88394207 forward strand		−1.86434	−73.17128	3.73E−22
1140924	A_23_P259003	PEX5L	Homo sapiens peroxisomal biogenesis factor 5-like (PEX5L), mRN.	NM_016559	−2	−100	2.22E−23
1153025	A_32_P26401	C21orf54	Homo sapiens chromosome 21 open reading frame 54 (C21orf54),	NR_024102	−2	−100	1.12E−22

Supplementary Table 2. Changes in gene expression of no infection with respect to TW295.

Sequence ID	Sequence Code	Primary Sequen	Sequence Description	Accession #	Log(Ratio)	Fold Change	P-value
2314508	A_19_P00806374		lincRNA:chr11:123940940-123956165 reverse strand		1.88621	76.95079	2.69E-22
2328902	A_33_P3492042		Homo sapiens D21S2091E mRNA sequence	AY063455	1.73744	54.63136	1.83E-21
1161927	A_32_P12202	FAM9C	family with sequence similarity 9, member (	BX648919	1.67738	47.57545	1.20E-18
882839	A_23_P503059	CNTN5	Homo sapiens contactin 5 (CNTN5), transc	NM_014361	1.27888	19.0056	2.29E-14
2329077	A_33_P3417840	C16orf47	Homo sapiens cDNA FLJ26184 fis, clone A	AK129695	1.23972	17.36683	5.07E-09
2313252	A_19_P00807378		lincRNA:chr8:102201249-102207349 forward strand		1.1023	12.65601	6.38E-19
1162083	A_32_P88240	KBTBD12	Homo sapiens kelch repeat and BTB (POZ	NM_207335	1.09276	12.3812	1.46E-11
2313939	A_33_P3374971	TRIM53	Homo sapiens tripartite motif-containing 5	NR_028346	1.03313	10.79269	2.85E-08
2314004	A_33_P3255051	LOC100509927	PREDICTED: Homo sapiens histone H2A t	XM_003119551	1.01002	10.23342	9.57E-08
2333629	A_19_P00327304		lincRNA:chr15:49013058-49023258 forward strand		0.93141	8.53902	1.52E-06
2313107	A_33_P3344633		Homo sapiens mRNA; cDNA DKFZp686I04	BX648590	0.92824	8.47697	5.08E-06
2317313	A_19_P00801752		lincRNA:chr21:44751672-44819297 reverse strand		0.90495	8.03435	0.00001
879364	A_23_P155057	CYTH4	Homo sapiens cytohesin 4 (CYTH4), mRNA	NM_013385	0.88451	7.66504	0.00003
2329571	A_33_P3352887	LOC388692	Homo sapiens hypothetical LOC388692 (L(	NR_027002	0.83947	6.90989	0.00014
2325759	A_33_P3310588		Unknown		0.83064	6.77072	0.00008
887362	A_24_P205994	EPGN	Homo sapiens epithelial mitogen homolog (	NM_001013442	0.82579	6.69555	0.00022
2322494	A_19_P00318045		lincRNA:chr2:20069253-20072338 reverse strand		0.80532	6.38738	0.00039
2320265	A_33_P3283535		Unknown		0.78895	6.15105	0.00069
2331081	A_19_P00322249		lincRNA:chr2:47558964-47559494 forward strand		0.78712	6.12516	1.26E-10
2314565	A_19_P00323009		lincRNA:chr13:114598075-114624290 forward strand		0.78092	6.03842	0.00089
1137521	A_24_P550924	BTBD19	Homo sapiens BTB (POZ) domain containir	NM_001136537	0.77171	5.91168	0.00121
2331548	A_19_P00810108		lincRNA:chrX:38716431-38792681 reverse strand		0.76067	5.76323	0.00029
2332022	A_33_P3314073		Unknown		0.75978	5.75154	0.00165
2314005	A_33_P3411165	MAGEA2B	Homo sapiens melanoma antigen family A,	NM_153488	0.74781	5.59509	0.00234
1158762	A_24_P214858	TREML2	Homo sapiens triggering receptor expresse	NM_024807	0.73936	5.48729	0.00289
2322838	A_33_P3244249		SH3 domain and tetratricopeptide repeats	AK127248	0.73281	5.40516	0.00331
2320008	A_33_P3420500	ZDHHC20	Homo sapiens zinc finger, DHHC-type cont	BC034944	0.73194	5.39436	0.00308
2325731	A_33_P3277688	LOC100132344	Homo sapiens cDNA FLJ43495 fis, clone P	AK125484	0.73123	5.38551	9.02E-14
1160709	A_23_P90980	NEU2	Homo sapiens sialidase 2 (cytosolic sialidas	NM_005383	0.72957	5.36502	0.00004
2317613	A_19_P00811555		lincRNA:chr14:96505728-96560235 reverse strand		0.72933	5.36206	0.00171
1142822	A_24_P6449	SAMD7	Homo sapiens sterile alpha motif domain co	NM_182610	0.72864	5.35349	0.0036
2319999	A_33_P3396562	LOC642414	PREDICTED: Homo sapiens putative tripar	XM_003119181	0.72714	5.33505	7.74E-12
1138717	A_24_P15898		Uncharacterized protein C1orf145 [Source	BC027909	0.72383	5.29454	0.00373
2322483	A_19_P00327099		lincRNA:chr8:138387843-138421643 reverse strand		0.71839	5.22871	0.00435
2330307	A_33_P3384492	LOC100127946	PREDICTED: Homo sapiens hypothetical p	XM_001717040	0.70844	5.11017	0.00265
2313950	A_33_P3279276	GRIP2	Homo sapiens glutamate receptor interacti	NM_001080423	0.70789	5.10382	0.00559
2331563	A_33_P3284611		Unknown		0.70451	5.06423	0.00576
2330387	A_19_P00320251		lincRNA:chr4:79567153-79602239 forward strand		0.69449	4.94867	0.00818
2319844	A_33_P3231858	LOC100133991	Homo sapiens similar to hCG1995169 (LOC	NR_024435	0.68964	4.89369	0.00485
1146028	A_23_P341527	DCST1	Homo sapiens DC-STAMP domain containi	NM_152494	0.68693	4.86331	0.00265
2314563	A_33_P3224181		DA918960 SMINT2 Homo sapiens cDNA cl	DA918960	0.68652	4.85871	0.00013
2327901	A_19_P00800621		lincRNA:chr21:29918229-30215104 reverse strand		0.68482	4.83967	0.00952
2318719	A_33_P3410274		Unknown		0.68478	4.83929	0.00874
2334309	A_33_P3258772	LOC100506816	PREDICTED: Homo sapiens hypothetical L	XR_108400	0.68433	4.83431	3.30E-07
1158774	A_32_P154223	LOC100505683	PREDICTED: Homo sapiens hypothetical L	XR_109654	0.68292	4.81855	0.00947
2314589	A_19_P00321664		lincRNA:chr4:6672454-6675524 forward strand		0.68289	4.81828	0.00024
2317444	A_33_P3354574		Unknown		0.67313	4.71121	0.00007
2312990	A_33_P3246774	LOC649201	PREDICTED: Homo sapiens paraneoplastic	XM_001127211	0.67091	4.68714	0.00627
2312477	A_33_P3295029	KBTBD12	Homo sapiens kelch repeat and BTB (POZ	NM_207335	0.66604	4.63495	0.00099
2313938	A_33_P3236628		branched chain keto acid dehydrogenase E	AL833143	0.65987	4.56957	0.0011
1155912	A_23_P114210	POU3F4	Homo sapiens POU class 3 homeobox 4 (P	NM_000307	0.65701	4.53953	0.00062
1159480	A_24_P56330	C13orf16	Homo sapiens chromosome 13 open readin	NM_152324	0.65596	4.52859	0.00165
1157226	A_23_P421811	C20orf132	Homo sapiens chromosome 20 open readin	NM_152503	0.65342	4.5022	0.00065
1143909	A_23_P55632	SERPINB3	Homo sapiens serpin peptidase inhibitor, cl	NM_006919	0.64229	4.3882	0.00007
1167454	A_23_P143512	HSF2BP	Homo sapiens heat shock transcription fac	NM_007031	0.63119	4.27747	0.00544
1142763	A_23_P2317	DDN	Homo sapiens dendrin (DDN), mRNA [NM_(	NM_015086	0.61958	4.16467	9.35E-07
2322364	A_33_P3353630		Unknown		0.6071	4.04673	3.68E-07
2316961	A_33_P3224190	SLC29A4	Homo sapiens solute carrier family 29 (nuc	NM_001040661	0.60657	4.04178	0.00132
2314124	A_19_P00315627		lincRNA:chr9:2493073-2521995 reverse strand		0.60254	4.00446	2.77E-08
885025	A_23_P421379	IGF2	Homo sapiens insulin-like growth factor 2 (	NM_000612	0.60111	3.9913	3.14E-10
2314046	A_33_P3244433	FAM188B	Homo sapiens cDNA FLJ33507 fis, clone B	AK090826	0.5865	3.85926	0.00213

Supplementary Table 2. Continued.

2331256	A_19_P00807573		lincRNA:chr11:64259424-64274774 reverse strand	0.58551	3.85041	0.00599
1155450	A_23_P399797	SMAD5OS	Homo sapiens SMAD family member 5 opp NR_026763	0.57417	3.75121	0.00026
883443	A_23_P74928	MR1	Homo sapiens major histocompatibility con NM_001531	0.55375	3.57891	0.001
2316221	A_19_P00328916		lincRNA:chr12:32555233-32563808 reverse strand	0.55289	3.57179	0.00573
2327311	A_33_P3241184		Unknown	0.54875	3.53793	1.43E-11
2314232	A_33_P3366824		trafficking protein particle complex 2 pseu AK310542	0.54839	3.53502	0.00089
2334855	A_33_P3418838		PREDICTED: Homo sapiens hypothetical p XM_001716008	0.54106	3.4758	0.00069
2318173	A_33_P3334205	TMEM184A	Homo sapiens transmembrane protein 184 NM_001097620	0.53748	3.44732	0.00881
2335884	A_33_P3285877		Unknown	0.53557	3.43219	0.004
2325148	A_19_P00802270		lincRNA:chr2:121828880-121902705 forward strand	0.51937	3.30652	0.0069
886458	A_23_P300100	PLA2G2D	Homo sapiens phospholipase A2, group IID NM_012400	0.5179	3.29536	0.00138
2320762	A_19_P00324706		lincRNA:chr11:72446627-72462602 forward strand	0.5153	3.27566	0.00121
2318395	A_33_P3382849	ACR	Homo sapiens acrosin (ACR), mRNA [NM_C NM_001097	0.51073	3.24139	3.29E-07
1146757	A_23_P170574	SNAI3	Homo sapiens snail homolog 3 (Drosophila) NM_178310	0.50549	3.20253	0.00944
2324523	A_19_P00801392		lincRNA:chr5:93988844-94003469 forward strand	0.50358	3.18846	0.00258
2332901	A_19_P00317818		lincRNA:chr9:102564752-102567347 reverse strand	0.50088	3.16866	0.00638
2315445	A_33_P3276112		Homo sapiens cDNA FLJ44789 fis, clone B AK126743	0.49152	3.10112	0.00021
1152070	A_23_P97096	OPTC	Homo sapiens opticin (OPTC), mRNA [NM_ NM_014359	0.48939	3.08594	1.21E-08
2312785	A_33_P3314559	RAB3C	Homo sapiens RAB3C, member RAS oncog NM_138453	0.48797	3.0759	5.84E-06
2323865	A_19_P00802508		lincRNA:chr2:12453499-12466124 forward strand	0.48247	3.03721	0.00042
1165450	A_23_P151975	RHCG	Homo sapiens Rh family, C glycoprotein (R NM_016321	0.47328	2.97358	0.00037
2318111	A_19_P00801580		lincRNA:chr22:25588075-25594350 reverse strand	0.47237	2.96737	1.04E-06
2317956	A_33_P3267198		cDNA FLJ43696 fis, clone TBAES2007964 XR_110423	0.47106	2.95841	0.00853
2318218	A_19_P00811414		lincRNA:chr1:84106887-84316837 forward strand	0.46861	2.94181	0.00076
2323910	A_19_P00805265		lincRNA:chr6:29701971-29740296 forward strand	0.4615	2.89403	0.00002
1137600	A_23_P20578	FAM166B	Homo sapiens family with sequence similar NM_001164310	0.45625	2.85926	0.0012
2330888	A_33_P3232066		Unknown	0.45584	2.85651	2.66E-08
1164435	A_23_P24311	ALDH3B2	Homo sapiens aldehyde dehydrogenase 3 f NM_000695	0.45438	2.84693	0.00075
2322491	A_33_P3390236	LOC338963	Homo sapiens epididymal protein pseudoge NR_034139	0.454	2.84445	0.00971
2313311	A_33_P3226557	PRR23B	Homo sapiens proline rich 23B (PRR23B), NM_001013650	0.45102	2.82503	0.00612
880992	A_23_P69497	CLEC3B	Homo sapiens C-type lectin domain family NM_003278	0.45085	2.82392	0.00024
2313054	A_19_P00807342		lincRNA:chr1:55833137-55834447 forward strand	0.43458	2.72005	0.00433
2317358	A_19_P00813235		lincRNA:chr2:75201792-75213492 reverse strand	0.43176	2.70247	0.0027
2332068	A_19_P00809429		lincRNA:chr19:37454235-37517910 reverse strand	0.4297	2.68969	0.00556
2321441	A_19_P00317854		lincRNA:chr19:56784137-56813412 forward strand	0.42942	2.68794	0.00042
1158076	A_24_P395814	CGB	Homo sapiens chorionic gonadotropin, beta NM_000737	0.42675	2.67145	0.00084
2317276	A_33_P3216200		Unknown	0.41982	2.62918	0.00507
2321338	A_19_P00325628		lincRNA:chr14:23764510-23770183 forward strand	0.41832	2.62011	0.00316
2323946	A_19_P00316437		lincRNA:chr4:15686304-15690595 forward strand	0.41812	2.61888	0.00524
2329623	A_33_P3255531	LOC100129216	Putative uncharacterized protein ENSP000 XM_001721605	0.41554	2.60341	0.00106
1155891	A_23_P100660	SERPINF1	Homo sapiens serpin peptidase inhibitor, cl NM_002615	0.41494	2.59983	0.00123
2326983	A_33_P3271470	GREB1L	Homo sapiens growth regulation by estrog NM_001142966	0.41355	2.59147	0.00154
2322406	A_19_P00811931		lincRNA:chr5:88215119-88544444 forward strand	0.41302	2.58833	0.00573
2329768	A_19_P00318328		lincRNA:chr16:11466321-11477551 forward strand	0.408	2.55857	0.00112
2314569	A_33_P3315819	LOC728208	PREDICTED: Homo sapiens hypothetical L XR_108453	0.40605	2.54715	0.00052
2318919	A_33_P3424367	TRIM14	Homo sapiens tripartite motif-containing 1 NM_033219	0.39892	2.50566	0.00153
2329085	A_19_P00330004		lincRNA:chr12:28168533-28183008 reverse strand	0.39822	2.5016	0.0073
1154635	A_23_P50276	ANGPTL6	Homo sapiens angiopoietin-like 6 (ANGPTI NM_031917	0.39708	2.49507	0.00083
2334687	A_33_P3262167		Unknown	0.39477	2.4818	0.00617
2322332	A_19_P00322412		lincRNA:chr6:6794382-6801404 reverse strand	0.39447	2.48012	0.00317
2328638	A_33_P3281010		Homo sapiens cDNA FLJ43862 fis, clone T AK125850	0.39289	2.47108	0.00001
2320515	A_19_P00323268		lincRNA:chr6:48044816-48070216 forward strand	0.39212	2.46671	0.00566
2316950	A_19_P00331084		lincRNA:chr2:203179905-203194480 forward strand	0.39014	2.45551	0.00187
2329312	A_33_P3224362	PCBP3	Homo sapiens poly(rC) binding protein 3 (P NM_020528	0.38934	2.45098	0.00662
2317335	A_33_P3234540		Unknown	0.38927	2.45058	4.24E-08
1146452	A_23_P122134	NMUR2	Homo sapiens neuromedin U receptor 2 (N NM_020167	0.38619	2.43326	0.00055
2331899	A_19_P00329800		lincRNA:chr1:204525202-204541352 forward strand	0.38559	2.42989	5.41E-08
2329972	A_19_P00805336		lincRNA:chr15:56580983-56592133 forward strand	0.38387	2.42033	0.00002
1150116	A_23_P303101	PCDHGC4	Homo sapiens protocadherin gamma subfa NM_032406	0.38328	2.41705	0.0003
2322385	A_19_P00800632		lincRNA:chr2:152619954-152626029 reverse strand	0.38306	2.41581	8.31E-07
2315276	A_33_P3265866		Unknown	0.38201	2.40996	1.42E-06
2319580	A_19_P00326743		lincRNA:chr2:32237771-32245946 forward strand	0.3771	2.38287	0.00373
2325776	A_19_P00316039		lincRNA:chr3:106531384-106556814 reverse strand	0.37642	2.37913	1.73E-07
887395	A_23_P1452	NPFFR1	Homo sapiens neuropeptide FF receptor 1 NM_022146	0.37249	2.35771	0.00493

Supplementary Table 2. Continued.

2322405	A_19_P00322979		lincRNA:chr20:37049674-37050891 forward strand	0.37173	2.35361	5.76E-06
1154483	A_23_P47709	FOLR2	Homo sapiens folate receptor 2 (fetal) (FO NM_000803	0.37074	2.34821	0.00043
2314133	A_33_P3233731		PREDICTED: Homo sapiens hypothetical L XM_001716016	0.36944	2.34122	0.00113
884962	A_23_P157593	CHRNA6	Homo sapiens cholinergic receptor, nicotini NM_004198	0.36826	2.33487	0.00331
2318660	A_19_P00322102		lincRNA:chr20:37075627-37079447 reverse strand	0.36787	2.33277	0.00611
2324370	A_33_P3367396	FAM177B	Homo sapiens family with sequence similar NM_207468	0.36758	2.33121	3.73E-07
2325163	A_33_P3332166	PRSS38	Homo sapiens protease, serine, 38 (PRSS3 NM_183062	0.36444	2.31441	0.00016
1158752	A_24_P791040	FBXW2	Homo sapiens F-box and WD repeat domai NM_012164	0.36406	2.31237	0.00532
2330340	A_33_P3410659	CLEC12B	Homo sapiens C-type lectin domain family NM_001129998	0.36088	2.29551	0.00345
1156982	A_23_P131683	MAPRE3	Homo sapiens microtubule-associated prot NM_012326	0.35318	2.2552	0.00332
2315477	A_33_P3245952	LOC648570	PREDICTED: Homo sapiens hypothetical L XR_114251	0.3528	2.25319	0.00641
2318589	A_19_P00316576		lincRNA:chrX:15693589-15706981 forward strand	0.3498	2.23767	5.29E-07
2332526	A_33_P3229577		Unknown	0.34964	2.23685	0.00021
2329608	A_33_P3345051	LOC645971	Homo sapiens hypothetical protein LOC64! NM_001195256	0.34675	2.22203	0.00031
2325487	A_19_P00800754		lincRNA:chr17:67543555-67550405 reverse strand	0.34306	2.20325	3.25E-06
881436	A_23_P349025	SAMD15	Homo sapiens sterile alpha motif domain cc NM_001010860	0.3405	2.19026	0.00702
1163020	A_23_P28598	DLX2	Homo sapiens distal-less homeobox 2 (DL NM_004405	0.34041	2.18984	8.48E-06
2332264	A_33_P3305655	BCL8	Homo sapiens B-cell CLL/lymphoma 8 (BC NR_027992	0.33954	2.18544	0.00503
2327154	A_33_P3299921	LOC100128869	PREDICTED: Homo sapiens hypothetical pi XM_001719518	0.33802	2.17782	0.00151
2317623	A_33_P3223106	ZNF890P	Homo sapiens zinc finger protein 890, pseu NR_034163	0.33742	2.17479	0.00085
2333960	A_19_P00808290		lincRNA:chr1:173968527-173991054 reverse strand	0.33539	2.16468	1.04E-06
883113	A_23_P26325	CCL17	Homo sapiens chemokine (C-C motif) ligan NM_002987	0.33438	2.15965	0.00546
2335320	A_33_P3375541	CD3D	Homo sapiens CD3d molecule, delta (CD3- NM_000732	0.33356	2.15554	0.00003
2326691	A_19_P00813402		lincRNA:chr6:107214007-107223757 reverse strand	0.33326	2.15408	0.00761
2334397	A_33_P3389599		Unknown BX457161	0.33103	2.14305	0.00543
2331879	A_33_P3274134	TMEM151B	Homo sapiens transmembrane protein 151E NM_001137560	0.33039	2.13986	1.40E-06
2333296	A_33_P3313528		Synthetic construct Homo sapiens gatewa CU693037	0.32982	2.13708	0.00738
2335629	A_33_P3296975	LOC390251	Homo sapiens cDNA FLJ36034 fis, clone T AK093353	0.32922	2.13413	0.00615
879351	A_23_P398566	NR4A3	Homo sapiens nuclear receptor subfamily 4 NM_006981	0.327	2.12324	1.65E-10
882169	A_24_P606538	GGNBP1	Homo sapiens gametogenetin binding prote NR_028361	0.32685	2.12249	0.00006
2321184	A_19_P00321910		lincRNA:chr15:69373187-69383734 forward strand	0.32619	2.11929	0.00013
2314868	A_19_P00321645		lincRNA:chr6:147185594-147232742 forward strand	0.32408	2.10901	0.00515
2316407	A_33_P3370875		Unknown	0.32325	2.10498	0.00348
2315860	A_33_P3260974		DA734158 NT2RP2 Homo sapiens cDNA cl DA734158	0.32109	2.09455	5.20E-06
2328112	A_33_P3291160		Unknown	0.31965	2.0876	0.00027
1165393	A_24_P100190	C21orf93	PREDICTED: Homo sapiens chromosome 2 XR_109683	0.31937	2.08626	4.96E-06
2326919	A_33_P3315704		Unknown	0.31749	2.07728	0.00065
2320663	A_33_P3365988		full-length cDNA clone CS0DB007YO17 of CR621509	0.317	2.07491	0.00013
2324288	A_19_P00805392		lincRNA:chr11:34718449-34894824 reverse strand	0.31167	2.04959	0.00451
2333360	A_19_P00331325		lincRNA:chr14:101389472-101539847 forward strand	0.31037	2.04347	0.00628
1151529	A_23_P83234	ZBTB6	Homo sapiens zinc finger and BTB domain NM_006626	0.30982	2.04088	0.00005
2332117	A_33_P3219121		Homo sapiens cDNA FLJ16443 fis, clone B AK131379	0.3091	2.0375	0.00305
1154014	A_24_P917819	ANKRD30BP2	Homo sapiens ankyrin repeat domain 30B NR_026916	0.30836	2.02472	7.21E-06
2312945	A_19_P00809185		lincRNA:chr19:15400850-15442450 forward strand	0.306	2.02303	0.00461
2329759	A_19_P00316778		lincRNA:chr2:114727021-114764885 reverse strand	0.30509	2.01878	0.00791
2327242	A_33_P3357283		gamma-glutamyltransferase 2 [Source:HGI XM_001715205	0.30406	2.01401	0.00717
2322510	A_33_P3383084		Unknown	0.30349	2.01134	0.00187
2327319	A_19_P00316467		lincRNA:chr3:98700043-98701392 forward strand	0.30349	2.01134	0.00063
1152101	A_23_P144071	COL7A1	Homo sapiens collagen, type VII, alpha 1 (C NM_000094	0.30264	2.00743	0.00022
2329178	A_19_P00315726		lincRNA:chrX:16498858-16499989 reverse strand	0.30218	2.00528	0.00157
2324678	A_33_P3303956		PREDICTED: Homo sapiens hypothetical L XR_110658	0.30144	2.0019	0.00422
2322404	A_33_P3414113		PREDICTED: Homo sapiens fatty-acid ami XM_001129139	0.30125	2.001	0.00174
2315944	A_33_P3225630	LOC400706	PREDICTED: Homo sapiens hypothetical L XR_111876	-0.30193	-2.00413	0.004
2326005	A_19_P00803131		lincRNA:chr1:247136652-247145277 forward strand	-0.30215	-2.00519	0.00068
2313209	A_19_P00804779		lincRNA:chr4:47840518-47846712 forward strand	-0.30216	-2.0052	0.00389
1155991	A_24_P723735		zinc finger protein 720 pseudogene 1 [Source:HGNC Symbc	-0.30283	-2.0083	0.00021
2313469	A_33_P3647427	ZNF29P	Human Kox26 mRNA for zinc finger proteir X52357	-0.3031	-2.00955	0.00001
2331829	A_19_P00327983		lincRNA:chr3:13807699-13818124 forward strand	-0.30364	-2.01208	0.00001
1158388	A_24_P933011	TMEM200C	Homo sapiens transmembrane protein 200(NM_001080209	-0.30376	-2.01263	0.00004
1148946	A_24_P230282	VCX2	Homo sapiens variable charge, X-linked 2 (NM_016378	-0.30385	-2.01305	0.00672
2318281	A_19_P00801549		lincRNA:chr9:2756150-2773400 forward strand	-0.30386	-2.01308	0.00003
2319144	A_19_P00327578		lincRNA:chr8:90726603-90741628 forward strand	-0.30397	-2.01358	0.00012
1154471	A_24_P330633	TAT	Homo sapiens tyrosine aminotransferase (1 NM_000353	-0.30409	-2.01413	0.0012
886284	A_23_P301846	CALCA	Homo sapiens calcitonin-related polypeptic NM_001033952	-0.30464	-2.01672	0.002

Supplementary Table 2. Continued.

1150043	A_24_P205036	ZNF676	Homo sapiens zinc finger protein 676 (ZNF NM_001001411	-0.30729	-2.02902	9.08E-06
2313404	A_19_P00327216		lincRNA:chr2:238021286-238040461 forward strand	-0.30927	-2.03829	5.33E-06
2329399	A_19_P00327193		lincRNA:chr5:131798076-131807951 reverse strand	-0.30966	-2.04015	5.40E-06
2328505	A_33_P3219454		Unknown	-0.31027	-2.04299	4.53E-06
2326359	A_19_P00319351		lincRNA:chr1:177994047-177994786 reverse strand	-0.31097	-2.04632	0.00092
1151888	A_24_P119131	TMEM120B	Homo sapiens transmembrane protein 120E NM_001080825	-0.31147	-2.04864	4.59E-06
2316705	A_19_P00331004		lincRNA:chr2:8001249-8036724 reverse strand	-0.31208	-2.05154	0.00022
879966	A_24_P131589	CD86	Homo sapiens CD86 molecule (CD86), tran NM_006889	-0.3127	-2.05449	8.16E-11
2335390	A_19_P00322365		lincRNA:chr11:65195730-65196514 forward strand	-0.31426	-2.06189	6.49E-06
2330431	A_33_P3305250		proteasome (prosome, macropain) 26S subunit, non-ATPas	-0.3144	-2.06253	0.00765
1157213	A_23_P402331	WFDC5	Homo sapiens WAP four-disulfide core don NM_145652	-0.31518	-2.06622	0.00606
1141309	A_24_P366122	ACBD4	Homo sapiens acyl-CoA binding domain co NM_024722	-0.3154	-2.06728	3.53E-06
2333356	A_33_P3268686	FBXW12	Homo sapiens F-box and WD repeat domai NM_207102	-0.31559	-2.06818	0.00698
2322123	A_19_P00331381		lincRNA:chr4:1036950-1047200 reverse strand	-0.31645	-2.07229	5.90E-06
2312881	A_33_P3262575	BAIAP2L1	Homo sapiens BAI1-associated protein 2-l NM_018842	-0.31677	-2.07379	0.00003
2319594	A_33_P3350549		Unknown	-0.3171	-2.0754	3.20E-06
2332603	A_19_P00320413		lincRNA:chr6:158658627-158666616 forward strand	-0.31712	-2.0755	0.00004
2336321	A_33_P3377346	LOC100132526	Homo sapiens FYVE, RhoGEF and PH dom NR_036481	-0.3178	-2.07873	0.00074
2316068	A_33_P3403254	ZNF878	Homo sapiens zinc finger protein 878 (ZNF NM_001080404	-0.31918	-2.08538	0.00098
2327568	A_33_P3397613		Unknown	-0.31937	-2.08628	0.00794
2326697	A_19_P00812923		lincRNA:chr11:3402374-3408174 reverse strand	-0.31938	-2.08629	0.00155
2315562	A_33_P3216621		Unknown	-0.31952	-2.08697	0.00013
2320519	A_33_P3519223		Unknown	-0.3197	-2.08786	2.84E-06
2316242	A_33_P3349384		Unknown	-0.31986	-2.08861	0.00002
2312589	A_33_P3254751	LOC100131355	Homo sapiens cDNA FLJ42223 fis, clone T AK124217	-0.32007	-2.08961	5.55E-06
2322341	A_33_P3327762		dedicator of cytokinesis 8 [Source:HGNC Symbol;Acc:1919	-0.3204	-2.0912	0.00033
2331396	A_19_P00332459		lincRNA:chr4:24517527-24528051 reverse strand	-0.32073	-2.09281	0.00005
2331481	A_19_P00329574		lincRNA:chr5:1544500-1567142 reverse strand	-0.32081	-2.09318	2.57E-06
880649	A_24_P183150	CXCL3	Homo sapiens chemokine (C-X-C motif) lig NM_002090	-0.32102	-2.0942	0.00011
2329459	A_33_P3309526	PTPRC	Homo sapiens protein tyrosine phosphatas NM_002838	-0.32232	-2.10051	5.96E-11
2328114	A_33_P3251640	LOC284440	Homo sapiens hypothetical LOC284440 (LC NR_026956	-0.32318	-2.10465	0.00108
1138925	A_23_P397543	NCRNA00174	Homo sapiens non-protein coding RNA 174 NR_026873	-0.32328	-2.10513	9.08E-06
2319869	A_33_P3209326		Homo sapiens cDNA FLJ39676 fis, clone S AK096995	-0.32448	-2.11095	0.00585
1161430	A_23_P370479	POFUT1	Homo sapiens protein O-fucosyltransferas NM_015352	-0.32596	-2.11816	2.83E-06
2335951	A_19_P00324030		lincRNA:chr1:79897287-79954287 reverse strand	-0.32609	-2.11879	0.00556
2326964	A_33_P3728979	FAM151B	Homo sapiens family with sequence similar NM_205548	-0.32806	-2.12843	0.0001
1159779	A_32_P85042	LOC100132815	HCG2000535cDNA FLJ32177 fis, clone PL AK056856	-0.32956	-2.13578	0.00012
2312680	A_33_P3391105	LRP1	Homo sapiens low density lipoprotein recep NM_002332	-0.3298	-2.13696	0.00016
2330012	A_19_P00331635		lincRNA:chr17:72093980-72101705 reverse strand	-0.3304	-2.13993	1.65E-06
2326661	A_33_P3396010		Advanced glycosylation end product-speci AY755628	-0.33171	-2.14637	0.00056
1163530	A_23_P319859	EYA2	Homo sapiens eyes absent homolog 2 (Dro NM_005244	-0.33191	-2.14737	0.00392
2321196	A_33_P3324655	C8orf41	Homo sapiens chromosome 8 open reading NM_025115	-0.3325	-2.15032	2.23E-06
2313913	A_33_P3356811	LCE1E	Homo sapiens late cornified envelope 1E (L NM_178353	-0.33318	-2.15367	1.19E-06
882236	A_23_P252236	KLKB1	Homo sapiens kallikrein B, plasma (Fletche NM_000892	-0.33366	-2.15605	0.00693
1159863	A_24_P323967	ZNF880	Homo sapiens zinc finger protein 880 (ZNF NM_001145434	-0.33376	-2.15656	1.52E-06
1157638	A_23_P106906	PPL	Homo sapiens periplakin (PPL), mRNA [NM NM_002705	-0.33904	-2.18292	0.00789
1137083	A_23_P28042	ZNF737	Homo sapiens zinc finger protein 737 (ZNF NM_001159293	-0.34014	-2.18849	8.43E-07
2319028	A_19_P00806762		lincRNA:chrX:73043635-73044133 forward strand	-0.34216	-2.19866	0.00433
2329744	A_19_P00327350		lincRNA:chr9:114435054-114447929 forward strand	-0.34442	-2.21013	0.00002
2332554	A_19_P00324198		lincRNA:chr2:74610317-74618792 forward strand	-0.34445	-2.21028	0.00536
2336326	A_19_P00800183		lincRNA:chr2:81861889-82101789 forward strand	-0.34474	-2.2118	4.12E-06
2329846	A_19_P00319795		lincRNA:chr19:56795502-56798051 reverse strand	-0.34488	-2.21248	0.0038
2320559	A_19_P00319186		lincRNA:chr2:39197219-39200415 forward strand	-0.34525	-2.21439	0.00562
2325344	A_33_P3345623	RREB1	Homo sapiens ras responsive element bind NM_001003700	-0.34588	-2.21758	0.00021
2319965	A_19_P00330834		lincRNA:chr10:65236069-65272719 forward strand	-0.34598	-2.21809	1.58E-06
2335098	A_19_P00812874		lincRNA:chr14:91315547-91326047 reverse strand	-0.34656	-2.22105	0.00015
2329987	A_19_P00803183		lincRNA:chr15:55466383-55471758 forward strand	-0.34774	-2.22711	0.00373
2314049	A_19_P00329233		lincRNA:chr22:25408700-25464025 reverse strand	-0.34812	-2.22907	4.90E-07
2327116	A_19_P00324431		lincRNA:chr7:55030831-55040930 forward strand	-0.34881	-2.2326	2.12E-06
2334740	A_33_P3243405	GPR182	Homo sapiens G protein-coupled receptor NM_007264	-0.3493	-2.23514	0.00012
887556	A_24_P322229	RASL10B	Homo sapiens RAS-like, family 10, member NM_033315	-0.34938	-2.23551	0.00095
1165847	A_32_P211248	LOC100131138	Homo sapiens hypothetical LOC100131138 NR_036513	-0.34981	-2.23773	4.45E-07
2319651	A_33_P3277883	LOC100129931	Homo sapiens hypothetical LOC100129931 NR_033828	-0.3507	-2.24232	0.00213
2315796	A_33_P3408034		Unknown	-0.35151	-2.24651	0.00448

Supplementary Table 2. Continued.

2328255	A_19_P00806091		lincRNA:chr18:14053400-14102850 reverse strand	-0.35236	-2.25093	0.00684
1167425	A_24_P717262	LOC100131479	PREDICTED: Homo sapiens zinc finger pro XM_001715121	-0.35405	-2.25971	0.00024
2320801	A_19_P00810084		lincRNA:chr2:37980796-38016431 reverse strand	-0.35411	-2.25999	1.23E-06
2316022	A_19_P00321087		lincRNA:chrX:149129110-149131024 reverse strand	-0.35482	-2.26371	0.00002
1154604	A_23_P161968	SLC22A10	Homo sapiens solute carrier family 22, men NM_001039752	-0.35498	-2.26451	0.0026
1159879	A_23_P357966	PCMTD1	Homo sapiens protein-L-isoaspartate (D-a NM_052937	-0.35735	-2.27695	0.00005
2319480	A_33_P3305203	LOC283588	PREDICTED: Homo sapiens hypothetical L XR_110237	-0.35912	-2.28621	3.26E-06
1160021	A_23_P309950		family with sequence similarity 178, membe AL833126	-0.35927	-2.28704	0.00425
882121	A_23_P22761	SHOX	Homo sapiens short stature homeobox (SH NM_006883	-0.35962	-2.28886	3.23E-06
2320503	A_19_P00325227		lincRNA:chr1:234908002-234918952 forward strand	-0.36062	-2.29416	2.33E-07
2328687	A_19_P00321979		lincRNA:chr15:58538381-58623829 reverse strand	-0.36069	-2.29449	0.00106
2326907	A_33_P3267482	KIAA1804	Homo sapiens mixed lineage kinase 4 (KIA/ NM_032435	-0.36099	-2.2961	2.28E-07
2330783	A_19_P00803630		lincRNA:chr8:2522118-2527693 forward strand	-0.36121	-2.29727	3.53E-07
2336271	A_33_P3357516	SLC10A7	Homo sapiens solute carrier family 10 (sod NM_032128	-0.36125	-2.29749	0.00594
2336353	A_33_P3629247	ANKMY1	Homo sapiens ankyrin repeat and MYND do NM_017844	-0.36177	-2.30023	2.50E-07
1152258	A_23_P379789	ST8SIA5	Homo sapiens ST8 alpha-N-acetyl-neuram NM_013305	-0.36192	-2.30103	0.00051
885197	A_32_P79434	PTPRN2	Homo sapiens protein tyrosine phosphatas NM_002847	-0.36504	-2.31761	6.12E-06
2319271	A_19_P00331620		lincRNA:chr12:96953169-96992769 forward strand	-0.36531	-2.31902	1.92E-07
886537	A_24_P151	KCNAB2	Homo sapiens potassium voltage-gated ch NM_003636	-0.36732	-2.32979	0.00482
2313467	A_33_P3280346	LOC100130857	Homo sapiens cDNA FLJ39731 fis, clone S AK097050	-0.36783	-2.33257	0.00067
2333726	A_33_P3437283	LOC643551	Homo sapiens cDNA FLJ46123 fis, clone T AK128004	-0.36814	-2.33421	0.00012
2327234	A_33_P3272808	PTCD3	Homo sapiens Pentatricopeptide repeat do NM_017952	-0.36833	-2.33525	0.00787
2335759	A_19_P00317364		lincRNA:chr17:75084917-75089529 reverse strand	-0.36887	-2.33814	0.00387
2326572	A_33_P3311473	ATP10D	Homo sapiens ATPase, class V, type 10D (NM_020453	-0.36959	-2.34204	0.00136
2321740	A_33_P3344504		amyloid beta (A4) precursor protein-bindin AK124794	-0.36976	-2.34294	8.74E-06
2320227	A_19_P00330815		lincRNA:chr11:2016408-2017024 forward strand	-0.36992	-2.34382	0.00072
2315161	A_33_P3327270	TMED7-TICAM2	Homo sapiens TMED7-TICAM2 readthroug NM_001164469	-0.37207	-2.35544	1.18E-07
2316746	A_33_P3229809	FLJ25363	Homo sapiens similar to hypothetical prote NM_001145553	-0.3724	-2.35722	0.00047
2332458	A_33_P3221960	IL18RAP	Homo sapiens interleukin 18 receptor acce NM_003853	-0.37264	-2.35853	1.18E-07
2327178	A_19_P00809587		lincRNA:chr8:96297524-96413824 reverse strand	-0.3728	-2.35941	0.00003
2335651	A_33_P3286011		Unknown	-0.37368	-2.36417	0.00685
2321044	A_19_P00325965		lincRNA:chr16:54944999-54959642 reverse strand	-0.37497	-2.37118	3.15E-06
2321597	A_33_P3366412		Unknown	-0.37499	-2.3713	0.00033
2315363	A_19_P00813490		lincRNA:chr19:42419485-42425035 reverse strand	-0.37566	-2.37496	4.55E-07
2313344	A_19_P00804439		lincRNA:chr21:44751672-44819297 forward strand	-0.37651	-2.37965	0.00934
2327912	A_33_P3259740		PREDICTED: Homo sapiens FLJ44881 (FL XR_110159	-0.37662	-2.38025	0.00156
2318457	A_33_P3273000	PTK2B	Homo sapiens cDNA FLJ46514 fis, clone T AK128371	-0.38015	-2.39964	7.29E-08
1157164	A_23_P346982	DTWD2	Homo sapiens DTW domain containing 2 (D NM_173666	-0.38107	-2.40477	0.00004
2320500	A_33_P3305438		Unknown	-0.38195	-2.40961	8.54E-08
2327886	A_19_P00325682		lincRNA:chr14:61529972-61553247 forward strand	-0.38453	-2.42397	5.70E-08
2329943	A_19_P00806723		lincRNA:chr16:72454899-72460874 forward strand	-0.38526	-2.42807	0.00032
2333681	A_19_P00812013		lincRNA:chr17:54898751-54910251 forward strand	-0.3862	-2.43332	0.00662
1148028	A_23_P155463	LRRC2	Homo sapiens leucine rich repeat containir NM_024512	-0.38679	-2.43663	1.59E-07
887603	A_23_P44849	KCTD13	Homo sapiens potassium channel tetrameri NM_178863	-0.38946	-2.45165	0.0001
2334377	A_19_P00806946		lincRNA:chr1:173758702-173765627 reverse strand	-0.38992	-2.45427	0.00005
2321374	A_33_P3267562	VAX2	Homo sapiens ventral anterior homeobox 2 NM_012476	-0.39112	-2.46104	3.87E-08
1154114	A_23_P79572	MGC16025	Homo sapiens hypothetical LOC85009 (MC NR_026664	-0.39164	-2.46401	1.68E-06
2324351	A_19_P00811084		lincRNA:chr2:200984605-201020245 forward strand	-0.39271	-2.47009	3.94E-08
1136711	A_24_P174775	NNT	nicotinamide nucleotide transhydrogenase [Source:HGNC S	-0.39282	-2.4707	0.00001
2324601	A_19_P00327469		lincRNA:chr3:160980656-160994006 forward strand	-0.39557	-2.48638	0.00021
2334779	A_33_P3403913		H.sapiens mRNA sequence (16p11.2). [X69 X69637	-0.39574	-2.48736	0.00166
2331302	A_19_P00330505		lincRNA:chr5:1544500-1567142 reverse strand	-0.39604	-2.48907	3.20E-08
2335532	A_19_P00323968		lincRNA:chr19:22019985-22034034 forward strand	-0.3973	-2.4963	0.0066
1162429	A_23_P34233	QPRT	Homo sapiens quinolinate phosphoribosyltr NM_014298	-0.39844	-2.50288	7.50E-06
1146316	A_24_P522631	TMEM201	Homo sapiens transmembrane protein 201 NM_001130924	-0.39847	-2.50303	2.46E-08
2315981	A_33_P3267463		regulatory factor X, 3 (influences HLA class II expression) [	-0.39976	-2.5105	0.00038
2331565	A_19_P00330145		lincRNA:chr2:192556480-192568755 forward strand	-0.3999	-2.51133	0.00313
2315941	A_33_P3212919		BX092453 Soares_testis_NHT Homo sapien BX092453	-0.40131	-2.51946	0.00003
2327803	A_33_P3279019	SERPINB13	Homo sapiens serpin peptidase inhibitor, cl NM_012397	-0.4015	-2.52059	6.99E-06
2325356	A_33_P3254375		PREDICTED: Homo sapiens similar to hCG XR_078855	-0.40239	-2.52573	0.00917
2327883	A_19_P00813006		lincRNA:chr4:53578624-53580302 reverse strand	-0.40469	-2.53916	9.59E-08
1142797	A_32_P187663	ZNF596	Homo sapiens zinc finger protein 596 (ZNF NM_001042416	-0.4066	-2.55038	4.52E-08
2316073	A_33_P3244568	LOC100128675	Homo sapiens hypothetical LOC100128675 NR_024561	-0.40676	-2.55129	0.00372
2319478	A_19_P00802496		lincRNA:chr8:16347304-16355399 reverse strand	-0.40816	-2.55955	2.16E-08

Supplementary Table 2. Continued.

1163490	A_24_P161813	LOC441666	Homo sapiens zinc finger protein 91 pseud NR_024380	-0.40862	-2.56225	1.43E-08
2313118	A_19_P00326936		lincRNA:chr13:54673999-54701589 forward strand	-0.40888	-2.5638	0.00169
1141478	A_24_P91701	C22orf43	Homo sapiens chromosome 22 open readin NM_016449	-0.40897	-2.56431	1.34E-08
2334462	A_33_P3231086		Unknown	-0.40964	-2.56827	4.72E-07
2312458	A_19_P00808500		lincRNA:chr9:4869500-4896050 forward strand	-0.4098	-2.5692	0.00265
2333677	A_33_P3221009		Homo sapiens cDNA FLJ45966 fis, clone P AK127863	-0.41	-2.57042	6.64E-06
1141449	A_23_P132889	CRMP1	Homo sapiens collapsin response mediator NM_001014809	-0.41333	-2.59017	6.07E-09
1140043	A_23_P416314	HRASLS5	Homo sapiens HRAS-like suppressor famil NM_054108	-0.41342	-2.59072	1.02E-08
1150263	A_24_P67585	VGLL3	Homo sapiens vestigial like 3 (Drosophila) (NM_016206	-0.41424	-2.59561	1.05E-08
2331415	A_19_P00802019		lincRNA:chr6:3897476-3911792 forward strand	-0.41629	-2.60792	0.00417
2320097	A_33_P3383009		Unknown	-0.41704	-2.6124	0.00544
2316820	A_33_P3216438	SPATA21	Homo sapiens spermatogenesis associated NM_198546	-0.41932	-2.62616	7.25E-09
1145439	A_23_P50232	AURKC	Homo sapiens aurora kinase C (AURKC), tr NM_001015878	-0.41941	-2.6267	7.18E-08
2327171	A_33_P3251871		Unknown	-0.41963	-2.62803	1.17E-08
2331566	A_33_P3570974		Homo sapiens chromosome 8 open reading BC043205	-0.42094	-2.63597	0.00009
1144810	A_32_P232035	LOC100270746	Homo sapiens hypothetical LOC100270746 NR_026776	-0.42131	-2.63822	1.66E-08
2326802	A_33_P3262022		Unknown	-0.42443	-2.65723	1.47E-08
2322698	A_33_P3311994	KIAA1239	Homo sapiens KIAA1239 (KIAA1239), mRN NM_001144990	-0.42523	-2.66213	0.00133
1143154	A_32_P790284	KATNAL2	Homo sapiens katanin p60 subunit A-like 2 NM_031303	-0.42541	-2.66327	0.00309
2318562	A_33_P3229672		PREDICTED: Homo sapiens similar to tubu XR_016188	-0.42913	-2.68613	0.00985
2322027	A_33_P3265415		Unknown	-0.42934	-2.68744	3.99E-09
2315136	A_33_P3381613		Unknown	-0.43038	-2.69389	0.0028
2334265	A_33_P3369920	LOC100128402	Homo sapiens cDNA FLJ42583 fis, clone B AK124574	-0.43239	-2.70637	0.00023
2328345	A_19_P00806932		lincRNA:chr18:4424357-4425507 reverse strand	-0.43284	-2.70922	0.00516
2316153	A_19_P00328923		lincRNA:chr5:127407926-127417781 reverse strand	-0.43297	-2.71001	5.49E-07
2335106	A_19_P00319939		lincRNA:chr2:114726900-114764885 reverse strand	-0.43313	-2.71102	0.00554
1167364	A_23_P92650	SLC25A2	Homo sapiens solute carrier family 25 (mit NM_031947	-0.43457	-2.72	0.00628
2327915	A_19_P00319155		lincRNA:chrX:115944898-115951828 reverse strand	-0.43596	-2.72874	0.00003
2335485	A_19_P00318845		lincRNA:chr10:4284540-4284771 reverse strand	-0.43612	-2.72973	0.00052
2325738	A_33_P3414494		Unknown	-0.438	-2.74156	0.00038
2329029	A_33_P3300635	PFKFB2	Homo sapiens 6-phosphofructo-2-kinase/ NM_006212	-0.43825	-2.74314	3.59E-17
2325276	A_19_P00325176		lincRNA:chr2:43102921-43110596 reverse strand	-0.44044	-2.75701	2.79E-09
2333678	A_33_P3423420	ZNF750	Homo sapiens zinc finger protein 750 (ZNF NM_024702	-0.44085	-2.75962	0.00021
2328177	A_33_P3336720	HAMP	Homo sapiens hepcidin antimicrobial peptic NM_021175	-0.44241	-2.76956	2.51E-17
2328696	A_19_P00811331		lincRNA:chr5:135519476-135525376 reverse strand	-0.44347	-2.77635	0.00115
2322846	A_33_P3380582		PREDICTED: Homo sapiens hypothetical L XR_108331	-0.44375	-2.77811	0.00019
1147621	A_24_P910060		Unknown	-0.44873	-2.81013	0.00016
2329734	A_19_P00319175		lincRNA:chr5:126187125-126189758 forward strand	-0.44883	-2.81082	0.00005
2318939	A_19_P00812662		lincRNA:chr12:5377839-5428564 reverse strand	-0.45419	-2.84568	3.70E-09
2319423	A_33_P3249529	PCNX	Homo sapiens pecanex homolog (Drosophil NM_014982	-0.45932	-2.87952	7.01E-10
2320680	A_19_P00327912		lincRNA:chr12:48037808-48056882 reverse strand	-0.46179	-2.89594	0.00019
2316033	A_33_P3423017		Unknown	-0.46196	-2.89706	0.00139
2314425	A_33_P3360758		potassium voltage-gated channel, KQT-like subfamily, mem	-0.46376	-2.90913	3.82E-09
880525	A_23_P366376	TDGF1	Homo sapiens teratocarcinoma-derived gr NM_003212	-0.46795	-2.93729	0.00036
2326972	A_19_P00806886		lincRNA:chr7:26681650-26696300 reverse strand	-0.46827	-2.93945	0.00084
2332902	A_19_P00812871		lincRNA:chr14:74163472-74168555 reverse strand	-0.47017	-2.95236	0.0001
1159870	A_23_P83579	ARNT2	Homo sapiens aryl-hydrocarbon receptor r NM_014862	-0.47109	-2.95863	1.91E-14
2330341	A_19_P00329166		lincRNA:chrX:107264119-107280819 forward strand	-0.4716	-2.96208	7.50E-10
1152611	A_23_P165239	ZNF208	Homo sapiens zinc finger protein 208 (ZNF NM_007153	-0.47211	-2.9656	3.44E-09
2335381	A_33_P3402943	LOC100128591	Homo sapiens cDNA FLJ46872 fis, clone U AK128705	-0.47718	-3.00038	0.00129
2332298	A_19_P00329303		lincRNA:chr4:127694975-127709825 reverse strand	-0.47764	-3.00355	0.00005
880639	A_23_P424900	C1orf88	Homo sapiens chromosome 1 open reading NM_181643	-0.47944	-3.01607	0.00004
2326061	A_33_P3349466	ANKRD12	Homo sapiens ankyrin repeat domain 12 (A NM_015208	-0.48053	-3.02363	4.75E-08
2319462	A_33_P3343972	RSPH1	Homo sapiens radial spoke head 1 homolog NM_080860	-0.48068	-3.02466	0.00985
2334959	A_19_P00323076		lincRNA:chr2:3172193-3183118 reverse strand	-0.48138	-3.02957	0.00012
2324247	A_33_P3264524		Unknown	-0.484	-3.04791	9.97E-06
2313588	A_19_P00325431		lincRNA:chr13:50657699-50697649 forward strand	-0.48438	-3.05054	0.00552
1156906	A_23_P69970	SOX30	Homo sapiens SRY (sex determining region NM_178424	-0.48507	-3.05542	0.00006
2319706	A_19_P00319435		lincRNA:chr5:42985502-43010278 forward strand	-0.48525	-3.0567	0.0006
882976	A_32_P130630	SLC16A7	Homo sapiens solute carrier family 16, men NM_004731	-0.48885	-3.0821	1.17E-13
2331303	A_19_P00329868		lincRNA:chr12:97876744-97905794 reverse strand	-0.48917	-3.0844	0.00686
884239	A_24_P47681	CAND1	Homo sapiens cullin-associated and neddy NM_018448	-0.49041	-3.0932	9.29E-20
2322919	A_19_P00805860		lincRNA:chr1:9229531-9229931 reverse strand	-0.49184	-3.10341	2.37E-10
1147079	A_23_P337849	CELF3	Homo sapiens CUGBP, Elav-like family me NM_007185	-0.49186	-3.10358	0.00032

Supplementary Table 2. Continued.

2330683	A_19_P00329806		lincRNA:chr12:54356095-54362515 reverse strand	-0.49253	-3.10837	1.07E-10
2327579	A_33_P3320212		Unknown	-0.49435	-3.12144	0.00331
1152443	A_24_P15877		Homo sapiens FKSG41 (FKSG41) mRNA, c AF333762	-0.49692	-3.13996	0.0033
2335604	A_33_P3230558	EML5	Homo sapiens echinoderm microtubule ass NM_183387	-0.5075	-3.21736	1.00E-06
2316268	A_19_P00322295		lincRNA:chr8:90737074-90737855 forward strand	-0.51111	-3.24425	2.23E-09
869406	A_23_P133408	CSF2	Homo sapiens colony stimulating factor 2 (NM_000758	-0.51236	-3.25358	0.00871
2313109	A_33_P3229017		Unknown	-0.51301	-3.25843	0.00065
2319903	A_33_P3412463	C10orf81	Homo sapiens chromosome 10 open readin NM_001193434	-0.51526	-3.27535	0.0028
2318904	A_33_P3417459	SCARNA9L	Homo sapiens small Cajal body-specific RI NR_023358	-0.51602	-3.28109	3.02E-11
2328020	A_19_P00319946		lincRNA:chr4:123621596-123626001 forward strand	-0.51765	-3.29344	0.00315
2333872	A_19_P00321375		lincRNA:chr15:25245397-25265969 reverse strand	-0.52211	-3.32742	0.00461
878987	A_23_P64661	ARHGAP9	Homo sapiens Rho GTPase activating prot NM_032496	-0.52456	-3.34624	6.86E-06
2319854	A_19_P00807924		lincRNA:chr4:103337452-103350952 forward strand	-0.52505	-3.35002	0.0001
2325170	A_33_P3278220		Rab9 effector protein with kelch motifs [Sc AL832249	-0.52621	-3.35902	1.69E-11
2332950	A_33_P3379046	LOC729770	PREDICTED: Homo sapiens hypothetical L XR_112442	-0.52955	-3.38495	1.44E-11
2324069	A_33_P3342720	ATP2A1	Homo sapiens ATPase, Ca++ transporting, NM_173201	-0.53156	-3.40062	0.00253
2322084	A_33_P3286552	NOX1	Homo sapiens NADPH oxidase 1 (NOX1), t NM_013955	-0.53255	-3.40837	0.00966
2333954	A_33_P3226761	SOX18	Homo sapiens SRY (sex determining region NM_018419	-0.53357	-3.41638	2.45E-18
2335229	A_33_P3411384		Unknown	-0.53376	-3.41787	1.13E-11
2319827	A_33_P3236416	GPR179	Homo sapiens G protein-coupled receptor NM_001004334	-0.53695	-3.44314	8.91E-06
1150618	A_23_P252276	ARHGEF38	Homo sapiens Rho guanine nucleotide excl NM_017700	-0.54082	-3.47389	0.0001
2328169	A_19_P00804666		lincRNA:chr19:57769688-57777413 reverse strand	-0.54653	-3.51987	5.72E-12
2314874	A_33_P3408514	SCNN1D	Homo sapiens sodium channel, nonvoltage- NM_001130413	-0.54892	-3.53931	0.00032
2318529	A_33_P3385516	CBLN4	Homo sapiens cerebellin 4 precursor (CBLI NM_080617	-0.55017	-3.54954	1.40E-06
1167892	A_23_P136571	C1orf14	Homo sapiens chromosome 1 open reading NM_030933	-0.55178	-3.56273	0.00146
2335869	A_33_P3403666	NDOR1	Homo sapiens NADPH dependent diflavin c NM_001144026	-0.55493	-3.58866	0.00008
2314085	A_19_P00321339		lincRNA:chr3:158458205-158461305 forward strand	-0.56196	-3.64716	0.0001
1162299	A_23_P79108	ATP8B3	Homo sapiens ATPase, aminophospholipid NM_138813	-0.56509	-3.67355	0.00155
2314250	A_33_P3389168		Unknown	-0.56811	-3.69919	0.00035
1156659	A_23_P350005	TRIML2	Homo sapiens tripartite motif family-like 2 NM_173553	-0.58515	-3.84728	0.0003
2334404	A_33_P3372094	LLGL1	Homo sapiens lethal giant larvae homolog 1 NM_004140	-0.58558	-3.85104	0.00764
1161697	A_24_P358868	ZNF728	PREDICTED: Homo sapiens zinc finger pro XM_001720936	-0.58892	-3.88081	8.00E-13
1156823	A_23_P426835	LOC729652	Homo sapiens mRNA; cDNA DKFZp434L05 AL834536	-0.59516	-3.93691	0.00503
2330608	A_33_P3231252	NHLH2	Homo sapiens nescient helix loop helix 2 (N NM_005599	-0.59724	-3.95586	5.17E-13
2312755	A_33_P3359183	AWAT2	Homo sapiens acyl-CoA wax alcohol acyltr NM_001002254	-0.59744	-3.95768	0.00078
2330830	A_19_P00812238		lincRNA:chr7:106416539-106423571 reverse strand	-0.60631	-4.03938	0.00295
879661	A_23_P321846	KCNS1	Homo sapiens potassium voltage-gated ch: NM_002251	-0.61442	-4.11546	0.00341
1142128	A_23_P335388	C17orf78	Homo sapiens chromosome 17 open readin NM_173625	-0.61938	-4.16274	0.00458
2328283	A_33_P3218491		Unknown	-0.62034	-4.17199	0.00062
2334258	A_33_P3558740	LOC254100	Homo sapiens cDNA FLJ33054 fis, clone T AK057616	-0.623	-4.19759	0.00557
886248	A_24_P380349	HTR1E	Homo sapiens 5-hydroxytryptamine (serot NM_000865	-0.62516	-4.21848	0.0026
2322787	A_33_P3213311		Unknown	-0.62975	-4.26333	5.47E-13
2325467	A_33_P3355937	LOC284276	Homo sapiens hypothetical LOC284276 (L(NR_015417	-0.63559	-4.32102	0.00743
889063	A_23_P1682	TMEM45B	Homo sapiens transmembrane protein 45B NM_138788	-0.63674	-4.3325	0.00366
2321140	A_33_P3346618	LOC441179	PREDICTED: Homo sapiens hypothetical L XR_112948	-0.63788	-4.3439	0.00004
1161571	A_23_P132760	TRH	Homo sapiens thyrotropin-releasing hormo NM_007117	-0.63889	-4.354	1.18E-06
2328115	A_19_P00328772		lincRNA:chr14:71971647-71983097 reverse strand	-0.64132	-4.37845	0.00714
2333743	A_33_P3547141	LOC100131298	Homo sapiens clone FLB4228 PRO1095 ml AF130053	-0.64866	-4.45307	0.00273
880654	A_24_P156769	MPL	Homo sapiens myeloproliferative leukemia NM_005373	-0.66797	-4.6555	0.00076
1145663	A_23_P209700	NMUR1	Homo sapiens neuromedin U receptor 1 (N NM_006056	-0.67388	-4.71928	3.67E-11
2328855	A_33_P3210228		zinc finger, CCHC domain containing 6 [Source:HGNC Syml	-0.67879	-4.77302	0.00021
2331609	A_19_P00326527		lincRNA:chr15:75201097-75208047 forward strand	-0.68055	-4.79234	1.01E-14
886083	A_23_P501722	TSPAN32	Homo sapiens tetraspanin 32 (TSPAN32), r NM_139022	-0.69226	-4.9233	0.00954
2312658	A_19_P00330893		lincRNA:chr11:129133890-129145940 reverse strand	-0.69638	-4.97032	0.00931
2328761	A_33_P3400278		Unknown	-0.69736	-4.98149	0.00768
2318600	A_33_P3209130	LOC100128496	CR738909 Soares_testis_NHT Homo sapier CR738909	-0.69904	-5.00076	0.00911
2333066	A_33_P3400309		Homo sapiens cDNA FLJ40251 fis, clone T AK097570	-0.7004	-5.01653	1.87E-07
2326278	A_33_P3350268		Unknown	-0.70327	-5.04971	0.00848
2335976	A_33_P3302577	GPR133	Homo sapiens G protein-coupled receptor NM_198827	-0.71011	-5.1299	0.00708
2321118	A_33_P3266769		Unknown	-0.71668	-5.20806	0.00678
2322484	A_33_P3421053	SELE	Homo sapiens selectin E (SELE), mRNA [N NM_000450	-0.73631	-5.44897	0.00421
1142788	A_32_P135517	LOC100506105	PREDICTED: Homo sapiens hypothetical L XR_109318	-0.7395	-5.48914	7.55E-13
2313679	A_19_P00321221		lincRNA:chr5:25413547-25446034 reverse strand	-0.74314	-5.53523	0.00331
2327353	A_33_P3326667		Unknown	-0.74382	-5.54397	0.0034

Supplementary Table 2. Continued.

2327859	A_19_P00800915		lincRNA:chr2:156244304-156300229 reverse strand	-0.74771	-5.59387	0.00316
2318966	A_33_P3358815	DEFB103A	Homo sapiens defensin, beta 103A (DEFB1 NM_001081551	-0.74796	-5.59709	0.00345
2327609	A_33_P3408137	LOC100130248	Homo sapiens cDNA FLJ42222 fis, clone T AK124216	-0.74815	-5.59947	0.00072
869436	A_23_P106194	FOS	Homo sapiens FBJ murine osteosarcoma v NM_005252	-0.75	-5.62342	1.13E-29
2315643	A_33_P3305288	PAQR9	Homo sapiens progestin and adipoQ recept NM_198504	-0.7526	-5.65719	0.00299
1159504	A_32_P216734	SPDYE3	Homo sapiens speedy homolog E3 (Xenopu NM_001004351	-0.7555	-5.69508	0.00037
2320939	A_19_P00322282		lincRNA:chr3:185003349-185009534 reverse strand	-0.75667	-5.71047	0.00254
2319943	A_19_P00331241		lincRNA:chr13:24717800-24728575 forward strand	-0.76386	-5.80575	0.00163
2322749	A_33_P3383626	ZSCAN5D	PREDICTED: Homo sapiens zinc finger and XM_001725568	-0.77343	-5.93519	2.98E-06
2319020	A_33_P3237393	ZNF140	Homo sapiens zinc finger protein 140 (ZNF NM_003440	-0.77577	-5.96716	0.00058
2335021	A_19_P00809030		lincRNA:chr2:156877046-157111432 reverse strand	-0.77756	-5.99185	0.00078
881348	A_23_P157007	TMEM176B	Homo sapiens transmembrane protein 176E NM_014020	-0.77776	-5.99461	0.00149
2312852	A_33_P3410232	LY6G6E	Homo sapiens lymphocyte antigen 6 compl NR_024541	-0.78134	-6.04419	0.00123
2320045	A_33_P3379081		601191189F1 NIH_MGC_7 Homo sapiens cE BE265920	-0.78569	-6.1051	0.00043
2314907	A_33_P3395848	VSIG10L	Homo sapiens V-set and immunoglobulin d NM_001163922	-0.78681	-6.12085	0.00094
2335486	A_33_P3280521	MFAP3L	Homo sapiens microfibrillar-associated pro NM_021647	-0.78994	-6.16504	0.00098
2318772	A_33_P3350858		Unknown	-0.79366	-6.21811	0.00044
2326429	A_19_P00328380		lincRNA:chr3:175987356-176039381 reverse strand	-0.79594	-6.25082	1.32E-16
2327988	A_33_P3724157	DERL3	Homo sapiens Der1-like domain family, mei NM_198440	-0.80575	-6.39371	0.00069
1158569	A_23_P152330	DOC2A	Homo sapiens double C2-like domains, alpl NM_003586	-0.80614	-6.39941	2.90E-07
2334561	A_33_P3301097		Unknown	-0.80975	-6.45289	2.18E-16
2321677	A_33_P3319276	FAM178B	Homo sapiens family with sequence similar NM_001122646	-0.81043	-6.46291	0.00065
2324909	A_19_P00326849		lincRNA:chr3:182651981-182660175 forward strand	-0.86309	-7.29609	0.00026
881671	A_23_P325606	ESX1	Homo sapiens ESX homeobox 1 (ESX1), ml NM_153448	-0.86696	-7.36147	5.84E-14
2317540	A_19_P00325785		lincRNA:chr3:162922581-162942606 reverse strand	-0.88275	-7.63401	1.94E-07
2334680	A_19_P00806038		lincRNA:chr8:125471969-125486269 reverse strand	-0.90779	-8.08711	3.66E-18
2333913	A_33_P3400302	TTTY18	Homo sapiens testis-specific transcript, Y- NR_001550	-0.9151	-8.22426	0.00196
1155346	A_24_P59236	RESP18	Homo sapiens regulated endocrine-specific NM_001007089	-0.93375	-8.58523	2.39E-06
2334705	A_33_P3230439	LOC100130148	Homo sapiens hypothetical LOC100130148 NR_024560	-0.95808	-9.07981	1.20E-06
2333644	A_33_P3283974		PREDICTED: Homo sapiens hypothetical L XR_109033	-0.96951	-9.32198	3.16E-14
2330300	A_33_P3809497	FLJ37505	Homo sapiens hypothetical LOC400087 (Fl NR_033987	-1.03264	-10.78065	7.50E-18
2327788	A_33_P3300747	ADHFE1	Homo sapiens alcohol dehydrogenase, iron NM_144650	-1.04097	-10.98942	1.06E-17
2323682	A_33_P3484806	FLJ21408	Homo sapiens cDNA: FLJ21408 fis, clone (AK025061	-1.05854	-11.44295	2.01E-08
2316576	A_19_P00806426		lincRNA:chr14:64116297-64131817 forward strand	-1.06559	-11.63031	3.42E-13
882483	A_24_P160380	PDLIM2	Homo sapiens PDZ and LIM domain 2 (mys NM_176871	-1.0757	-11.90407	8.28E-09
1144183	A_23_P131588	BMP10	Homo sapiens bone morphogenetic protein NM_014482	-1.10293	-12.67441	1.51E-09
2334587	A_33_P3350744		Unknown	-1.13005	-13.49115	3.49E-12
2314863	A_33_P3337569	C4orf40	Homo sapiens chromosome 4 open reading NM_214711	-1.18329	-15.25076	1.64E-11
2326840	A_19_P00801412		lincRNA:chr21:10701104-10873604 forward strand	-1.20468	-16.02052	4.16E-12
2334781	A_33_P3358277		potassium channel, subfamily K, member 1 AK090902	-1.20969	-16.20643	2.52E-12
2334273	A_33_P3601802		AGENCOURT_13978263 NIH_MGC_172 Hor CD049182	-1.33241	-21.49884	4.65E-20
2334403	A_33_P3322348	LOC100128942	cDNA FLJ46600 fis, clone THYMU3047144 AK128457	-1.59547	-39.3979	[illegible]49E-20
2334194	A_19_P00802785		lincRNA:chr3:72644685-72734460 reverse strand	-1.64462	-44.11792	1.53E-20
1150505	A_23_P155539	POU1F1	Homo sapiens POU class 1 homeobox 1 (P NM_000306	-1.85697	-71.93966	8.77E-23
2334402	A_19_P00805097		lincRNA:chr5:88390860-88394207 forward strand	-1.86544	-73.35722	3.70E-22
1153025	A_32_P26401	C21orf54	Homo sapiens chromosome 21 open readin NR_024102	-2	-100	1.10E-22
1140924	A_23_P259003	PEX5L	Homo sapiens peroxisomal biogenesis facto NM_016559	-2	-100	2.21E-23

Supplementary Table 3. Changes in gene expression of no infection with respect to TW295CND.

Sequence ID	Sequence Code	Primary Sequence	Sequence Description	Accession #	Log(Ratio)	Fold Change	P-value
2314508	A_19_P00806374		lincRNA:chr11:123940940-123956165 reverse strand		1.90317	80.01509	2.43E-22
1161927	A_32_P12202	FAM9C	family with sequence similarity 9, member C [Source:HGNC	BX648919	1.68436	48.34593	1.12E-18
2328902	A_33_P3492042		Homo sapiens D21S2091E mRNA sequence. [AY063455]	AY063455	1.62944	42.60312	2.84E-21
882839	A_23_P503059	CNTN5	Homo sapiens contactin 5 (CNTN5), transcript variant 1, mI	NM_014361	1.31443	20.62667	7.64E-15
2331081	A_19_P00322249		lincRNA:chr2:47558964-47559494 forward strand		1.26467	18.39372	9.49E-14
2312785	A_33_P3314559	RAB3C	Homo sapiens RAB3C, member RAS oncogene family (RAB:	NM_138453	1.15607	14.32425	5.11E-11
1162083	A_32_P88240	KBTBD12	Homo sapiens kelch repeat and BTB (POZ) domain containi	NM_207335	1.12643	13.37922	3.05E-12
2313939	A_33_P3374971	TRIM53	Homo sapiens tripartite motif-containing 53 (TRIM53), non-	NR_028346	1.0753	11.89314	7.93E-09
2334309	A_33_P3258772	LOC100506816	PREDICTED: Homo sapiens hypothetical LOC100506816 (L	XR_108400	1.04514	11.09533	1.08E-09
2313252	A_19_P00807378		lincRNA:chr8:102201249-102207349 forward strand		0.99436	9.87086	3.86E-18
2313107	A_33_P3344633		Homo sapiens mRNA; cDNA DKFZp686I04191 (from clone I	BX648590	0.96599	9.24674	1.98E-06
2317313	A_19_P00801752		lincRNA:chr21:44751672-44819297 reverse strand		0.93959	8.70132	4.62E-06
2314563	A_33_P3224181		DA918960 SMINT2 Homo sapiens cDNA clone SMINT20039	DA918960	0.92048	8.32677	0.00001
2329077	A_33_P3417840	C16orf47	Homo sapiens cDNA FLJ26184 fis, clone ADG04431. [AK12	AK129695	0.90927	8.11464	4.94E-08
1137600	A_23_P20578	FAM166B	Homo sapiens family with sequence similarity 166, member	NM_001164310	0.89564	7.86401	6.53E-06
2325731	A_33_P3277688	LOC100132344	Homo sapiens cDNA FLJ43495 fis, clone PEBLM2000338. [	AK125484	0.86967	7.40742	9.11E-16
887362	A_24_P205994	EPGN	Homo sapiens epithelial mitogen homolog (mouse) (EPGN), i	NM_001013442	0.8549	7.15977	0.00012
2312477	A_33_P3295029	KBTBD12	Homo sapiens kelch repeat and BTB (POZ) domain containi	NM_207335	0.84136	6.93998	0.00021
2314565	A_19_P00323009		lincRNA:chr13:114598075-114624290 forward strand		0.8108	6.46842	0.00053
2314005	A_33_P3411165	MAGEA2B	Homo sapiens melanoma antigen family A, 2B (MAGEA2B), i	NM_153488	0.78697	6.12308	0.00128
1143909	A_23_P55632	SERPINB3	Homo sapiens serpin peptidase inhibitor, clade B (ovalbumin	NM_006919	0.78693	6.12255	9.99E-06
1152070	A_23_P97096	OPTC	Homo sapiens opticin (OPTC), mRNA [NM_014359]	NM_014359	0.78241	6.05918	3.02E-13
1158762	A_24_P214858	TREML2	Homo sapiens triggering receptor expressed on myeloid cell	NM_024807	0.7777	5.99373	0.00165
1138717	A_24_P15898		Uncharacterized protein C1orf145 [Source:UniProtKB/Swi:	BC027909	0.73879	5.48018	0.00299
2322483	A_19_P00327099		lincRNA:chr8:138387843-138421643 reverse strand		0.73821	5.47285	0.00323
2314004	A_33_P3255051	LOC100509927	PREDICTED: Homo sapiens histone H2A type 1-like (LOC1	XM_003119551	0.73676	5.4546	6.53E-07
2327998	A_19_P00322181		lincRNA:chr9:132265766-132275963 reverse strand		0.72252	5.27865	0.00544
1147952	A_23_P92120	SNTN	Homo sapiens sentan, cilia apical structure protein (SNTN),	NM_001080537	0.71986	5.24643	0.00671
2323167	A_33_P3223488	FRY	Homo sapiens furry homolog (Drosophila) (FRY), mRNA [NM	NM_023037	0.71923	5.23881	0.00181
2317358	A_19_P00813235		lincRNA:chr2:75201792-75213492 reverse strand		0.71402	5.17626	0.00007
885025	A_23_P421379	IGF2	Homo sapiens insulin-like growth factor 2 (somatomedin A)	NM_000612	0.71329	5.16758	2.60E-12
1168250	A_24_P940086	NXPH3	Homo sapiens neurexophilin 3 (NXPH3), mRNA [NM_007225	NM_007225	0.71218	5.15437	0.00651
2316633	A_33_P3694746		Homo sapiens cDNA FLJ32224 fis, clone PLACE6004336. [	AK056786	0.70889	5.11556	0.00009
2318719	A_33_P3410274		Unknown		0.70841	5.10987	0.00846
2313467	A_33_P3280346	LOC100130857	Homo sapiens cDNA FLJ39731 fis, clone SMINT2015745. [/	AK097050	0.70703	5.09364	0.00726
2316180	A_19_P00802762		lincRNA:chr11:68040524-68046124 forward strand		0.70684	5.09143	0.00647
2327743	A_33_P3236322		Homo sapiens cDNA FLJ46124 fis, clone TESTI2040372. [A	AK128005	0.70521	5.07237	0.00666
2314124	A_19_P00315627		lincRNA:chr9:2493073-2521995 reverse strand		0.69971	5.00853	1.01E-09
1158774	A_32_P154223	LOC100505683	PREDICTED: Homo sapiens hypothetical LOC100505683 (L	XR_109654	0.69863	4.99613	0.0077
1142822	A_24_P6449	SAMD7	Homo sapiens sterile alpha motif domain containing 7 (SAM	NM_182610	0.68887	4.88504	0.00331
2331574	A_33_P3248405	NRK	Homo sapiens Nik related kinase, mRNA (cDNA clone IMAG	BC108702	0.68723	4.86662	0.00584
2329623	A_33_P3255531	LOC100129216	Putative uncharacterized protein ENSP00000406391 [Sour	XM_001721605	0.67661	4.74906	0.00001
2313786	A_33_P3226395	LOC389634	Homo sapiens hypothetical LOC389634 (LOC389634), non-	NR_024420	0.67279	4.70746	0.00504
2322295	A_19_P00322426		lincRNA:chr7:26438479-26533639 forward strand		0.66829	4.65897	0.00061
1136696	A_32_P79591	ZNF665	Homo sapiens zinc finger protein 665 (ZNF665), mRNA [NM	NM_024733	0.64473	4.41295	0.00918
2319999	A_33_P3396562	LOC642414	PREDICTED: Homo sapiens putative tripartite motif-contai	XM_003119181	0.63358	4.30109	8.20E-11
2327911	A_33_P3331831	DKFZp451A211	PREDICTED: Homo sapiens DKFZp451A211 protein (DKFZ	XR_114721	0.6292	4.25796	0.0034
2316429	A_33_P3222228	FUT6	Homo sapiens fucosyltransferase 6 (alpha (1,3) fucosyltrans	NM_000150	0.62622	4.22879	0.00713
2320579	A_33_P3235721	C11orf87	Homo sapiens chromosome 11 open reading frame 87 (C11	NM_207645	0.6155	4.12569	0.00169
2334591	A_19_P00806184		lincRNA:chr5:81650244-81658219 reverse strand		0.61245	4.09683	0.00915
2313712	A_19_P00801712		lincRNA:chr17:73586330-73600280 reverse strand		0.61005	4.07431	0.0068
2319626	A_19_P00317553		lincRNA:chr16:83967316-83967785 forward strand		0.60328	4.01123	0.00625
2314544	A_33_P3352677		Novel protein [Source:UniProtKB/TrEMBL;Acc:B1AHE6] [I	BG222044	0.60216	4.00095	1.66E-06
2318895	A_33_P3228510	EPB41L1	Homo sapiens erythrocyte membrane protein band 4.1-like	NM_177996	0.58544	3.84983	0.00502
2331563	A_33_P3284611		Unknown		0.58234	3.8224	0.00945
2322531	A_19_P00810346		lincRNA:chr14:35749974-35756874 reverse strand		0.57197	3.73227	0.0009
2324039	A_19_P00805400		lincRNA:chr1:211564402-211589752 reverse strand		0.56982	3.71382	0.00376
2329272	A_33_P3372647		V1-11 protein Fragment [Source:UniProtKB/TrEMBL;Acc:(	DQ098690	0.56759	3.69477	0.00552
2322838	A_33_P3244249		SH3 domain and tetratricopeptide repeats 2 [Source:HGNC	AK127248	0.56205	3.64797	0.008
2317586	A_19_P00320207		lincRNA:chr2:242832785-242833476 reverse strand		0.5615	3.64337	0.00049
881873	A_23_P319583	RIMS3	Homo sapiens regulating synaptic membrane exocytosis 3 (I	NM_014747	0.54866	3.53718	0.00095
886673	A_23_P126613	AQP10	Homo sapiens aquaporin 10 (AQP10), mRNA [NM_080429]	NM_080429	0.5486	3.53674	0.00874
2328621	A_33_P3317483	CEMP1	Homo sapiens cementum protein 1 (CEMP1), mRNA [NM_00	NM_001048212	0.54136	3.47822	0.0026
2331740	A_19_P00812454		lincRNA:chr7:22666693-22667114 reverse strand		0.5353	3.43008	0.00056
882611	A_32_P14721	DNAH12	Homo sapiens dynein, axonemal, heavy chain 12 (DNAH12),	NM_178504	0.51989	3.31048	0.00241
2313938	A_33_P3236628		branched chain keto acid dehydrogenase E1, alpha polypept	AL833143	0.51407	3.26642	0.00253
2328317	A_19_P00811207		lincRNA:chr6:108346507-108355057 reverse strand		0.51283	3.25712	0.00489
2325078	A_33_P3246523	OR2T1	Homo sapiens olfactory receptor, family 2, subfamily T, men	NM_030904	0.51199	3.25083	0.0014

Supplementary Table 3. Continued.

2321730	A_33_P3503408	LOC221946	Homo sapiens hypothetical protein LOC221946, mRNA (cDI	BC044606	0.51086	3.24237	0.00686
2328383	A_19_P00805092		lincRNA:chr2:172618479-172626629 reverse strand		0.51046	3.2394	0.0087
2322364	A_33_P3353630		Unknown		0.49993	3.16175	3.20E-06
2334182	A_19_P00316874		lincRNA:chr2:129153697-129402710 forward strand		0.49322	3.11329	0.00661
2334835	A_33_P3371341	FAM66C	Homo sapiens family with sequence similarity 66, member C	NR_026788	0.49106	3.09782	0.00286
2315322	A_19_P00319407		lincRNA:chr4:53609750-53611888 reverse strand		0.48737	3.07165	0.00325
1144046	A_24_P291231	PER3	Homo sapiens period homolog 3 (Drosophila) (PER3), mRNA	NM_016831	0.48207	3.03437	0.0002
2325373	A_33_P3398693	MFSD2B	Homo sapiens major facilitator superfamily domain containir	NM_001080473	0.48168	3.03163	0.00164
2329418	A_33_P3550618	LOC400541	Homo sapiens cDNA FLJ38747 fis, clone KIDNE2012361. [A	AK096066	0.48073	3.025	0.00553
1164008	A_24_P203315	CNIH2	Homo sapiens cornichon homolog 2 (Drosophila) (CNIH2), m	NM_182553	0.47543	2.98833	0.00292
1159204	A_32_P146113	C6orf81	Homo sapiens chromosome 6 open reading frame 81 (C6orf	NM_145028	0.47373	2.97666	0.00718
1142175	A_23_P33558	LOC729164	PREDICTED: Homo sapiens hCG1732469 (LOC729164), mis	XR_109936	0.4714	2.96072	0.00715
2319581	A_33_P3257252	ZNF551	Homo sapiens zinc finger protein 551 (ZNF551), mRNA [NM	NM_138347	0.46794	2.93726	0.00642
2325148	A_19_P00802270		lincRNA:chr2:121828880-121902705 forward strand		0.46572	2.92229	0.0095
2325475	A_19_P00800274		lincRNA:chr8:91113099-91397399 forward strand		0.46411	2.91148	0.00274
885032	A_23_P253221	ARHGEF4	Homo sapiens Rho guanine nucleotide exchange factor (GEI	NM_032995	0.4612	2.89201	0.00821
1139721	A_24_P187614	LOC100129648	Homo sapiens cDNA FLJ40318 fis, clone TESTI2030556. [A	AK097637	0.45723	2.86572	0.00097
2319130	A_33_P3384078	AKD1	Homo sapiens cDNA FLJ16163 fis, clone BRCAN2014229. [	AK131244	0.4544	2.84708	0.00864
1146573	A_23_P390443	SHISA7	Homo sapiens shisa homolog 7 (Xenopus laevis) (SHISA7), r	NM_001145176	0.45424	2.84606	0.00314
2332583	A_33_P3241696		solute carrier family 45, member 2 [Source:HGNC Symbol;A	BC003597	0.45286	2.83697	0.00081
2321613	A_33_P3331125	SLC2A12	Homo sapiens solute carrier family 2 (facilitated glucose tra	NM_145176	0.45142	2.82761	0.0076
2316065	A_33_P3242632	LOC646743	Homo sapiens hypothetical LOC646743 (LOC646743), non-	NR_033930	0.45049	2.82153	0.00354
2331898	A_33_P3408811		Unknown		0.43876	2.74638	0.00004
2314514	A_33_P3323059		Homo sapiens cDNA, FLJ97323. [AK307375]	AK307375	0.43319	2.71136	0.00069
2329571	A_33_P3352887	LOC388692	Homo sapiens hypothetical LOC388692 (LOC388692), non-	NR_027002	0.4295	2.68846	0.00478
1145990	A_32_P180741	TNK2	Homo sapiens tyrosine kinase, non-receptor, 2 (TNK2), tran	NM_001010938	0.42253	2.64563	0.00009
1140655	A_24_P402415	SYT14	Homo sapiens synaptotagmin XIV (SYT14), transcript variar	NM_153262	0.41972	2.62858	0.0011
2325268	A_19_P00811072		lincRNA:chr7:45019225-45026725 forward strand		0.41115	2.5772	0.00007
2335146	A_19_P00324823		lincRNA:chr12:13116233-13122083 reverse strand		0.40683	2.55171	0.00277
2333360	A_19_P00331325		lincRNA:chr14:101389472-101539847 forward strand		0.40178	2.52218	0.00047
881215	A_23_P63158	SPTA1	Homo sapiens spectrin, alpha, erythrocytic 1 (elliptocytosis	NM_003126	0.3988	2.50496	0.0022
2318879	A_19_P00327442		lincRNA:chr4:56803193-56814239 forward strand		0.39721	2.49578	0.00016
2316456	A_33_P3380493	SH3BGRL3	Homo sapiens SH3 domain binding glutamic acid-rich protei	NM_031286	0.39538	2.48533	0.00003
1160422	A_23_P165380	CRYGA	Homo sapiens crystallin, gamma A (CRYGA), mRNA [NM_01	NM_014617	0.39447	2.48013	0.00014
1146142	A_23_P63432	RHBDL2	Homo sapiens rhomboid, veinlet-like 2 (Drosophila) (RHBDL	NM_017821	0.39444	2.47991	0.00232
2334855	A_33_P3418838		PREDICTED: Homo sapiens hypothetical protein LOC10013	XM_001716008	0.38759	2.44112	0.00401
1160709	A_23_P90980	NEU2	Homo sapiens sialidase 2 (cytosolic sialidase) (NEU2), mRN	NM_005383	0.38578	2.43097	0.0025
2322406	A_19_P00811931		lincRNA:chr5:88215119-88544444 forward strand		0.38417	2.42195	0.00664
2322510	A_33_P3383084		Unknown		0.38039	2.40099	0.00018
2332526	A_33_P3229577		Unknown		0.37881	2.39225	0.00006
2321048	A_33_P3314500	MUC6	Homo sapiens mucin 6, oligomeric mucus/gel-forming (MUC	NM_005961	0.37765	2.38591	0.00809
2320006	A_33_P3323939	LIPN	Homo sapiens lipase, family member N (LIPN), mRNA [NM_0	NM_001102469	0.36945	2.34129	3.80E-07
1143171	A_24_P175187	SAMD9	Homo sapiens sterile alpha motif domain containing 9 (SAM	NM_017654	0.3688	2.33775	0.00061
1165812	A_23_P106069		Human mRNA for T cell receptor V alpha gene segment V-	X58747	0.36684	2.32722	0.00511
1158752	A_24_P791040	FBXW2	Homo sapiens F-box and WD repeat domain containing 2 (F	NM_012164	0.36671	2.32652	0.0041
1144104	A_23_P128993	GZMH	Homo sapiens granzyme H (cathepsin G-like 2, protein h-C	NM_033423	0.36577	2.32149	0.00158
1164435	A_23_P24311	ALDH3B2	Homo sapiens aldehyde dehydrogenase 3 family, member B	NM_000695	0.36295	2.30647	0.00272
2329028	A_19_P00316047		lincRNA:chr18:37236595-37243620 reverse strand		0.36198	2.30135	0.00136
2333629	A_19_P00327304		lincRNA:chr15:49013058-49023258 forward strand		0.36127	2.29759	0.00215
1165207	A_24_P212234	SLC6A18	Homo sapiens solute carrier family 6, member 18 (SLC6A18	NM_182632	0.35541	2.2668	0.00008
1161094	A_23_P325887	TBC1D8B	Homo sapiens TBC1 domain family, member 8B (with GRAM	NM_017752	0.35285	2.25348	0.0046
2331931	A_19_P00812681		lincRNA:chr2:39721271-39800646 forward strand		0.35208	2.24945	0.00009
1155891	A_23_P100660	SERPINF1	Homo sapiens serpin peptidase inhibitor, clade F (alpha-2 a	NM_002615	0.35171	2.24754	0.00289
1153205	A_23_P26072		HCG37415PRO1914 ; [Source:UniProtKB/TrEMBL;Acc:Q9L	AF118084	0.35166	2.24731	0.0001
1163020	A_23_P28598	DLX2	Homo sapiens distal-less homeobox 2 (DLX2), mRNA [NM_C	NM_004405	0.35137	2.24578	2.74E-06
2333034	A_19_P00810219		lincRNA:chr6:12005039-12011214 forward strand		0.34903	2.23375	6.72E-06
2321189	A_19_P00322525		lincRNA:chr4:6672449-6675450 forward strand		0.34585	2.21745	0.0044
2320879	A_33_P3232742		Unknown		0.33795	2.17746	0.0088
1146199	A_32_P225659	UTS2D	Homo sapiens urotensin 2 domain containing (UTS2D), mRN	NM_198152	0.3368	2.17172	0.00704
2332005	A_33_P3240972	LOC100134285	PREDICTED: Homo sapiens hypothetical protein LOC10013	XM_003119593	0.33523	2.16385	0.00063
2327319	A_19_P00316467		lincRNA:chr3:98700043-98701392 forward strand		0.33481	2.16175	0.00018
2327088	A_33_P3313456	CXorf30	Homo sapiens chromosome X open reading frame 30 (CXorl	NM_001098843	0.33126	2.14418	0.00545
881965	A_23_P399078	TIMP3	Homo sapiens TIMP metallopeptidase inhibitor 3 (TIMP3), m	NM_000362	0.33077	2.14174	0.00058
2318111	A_19_P00801580		lincRNA:chr22:25588075-25594350 reverse strand		0.32967	2.13633	0.0001
2324929	A_33_P3356070	LPIN1	Homo sapiens lipin 1 (LPIN1), mRNA [NM_145693]	NM_145693	0.32938	2.1349	0.0001
2326442	A_33_P3383816	LOC400657	Homo sapiens hypothetical LOC400657 (LOC400657), non-	NR_024484	0.32693	2.12289	0.00608
2324386	A_19_P00802741		lincRNA:chr2:179375079-179386329 reverse strand		0.32542	2.11555	1.89E-06
2329326	A_33_P3210349		cDNA FLJ44904 fis, clone BRAMY3005656 [Source:UniPro	XR_111612	0.32348	2.1061	0.00436
2336081	A_33_P3388822	ZNF233	Homo sapiens zinc finger protein 233 (ZNF233), mRNA [NM	NM_181756	0.31811	2.08022	0.00169
1162207	A_23_P166526	RIBC2	Homo sapiens RIB43A domain with coiled-coils 2 (RIBC2), r	NM_015653	0.3172	2.07585	0.00705
1146871	A_23_P722	SYCP1	Homo sapiens synaptonemal complex protein 1 (SYCP1), m	NM_003176	0.31452	2.0631	0.00383

Supplementary Table 3. Continued.

2317335	A_33_P3234540		Unknown		0.31417	2.06143	3.61E-06
2323001	A_33_P3254708	ARHGAP40	Homo sapiens Rho GTPase activating protein 40 (ARHGAP4	NM_001164431	0.31331	2.05735	0.00387
1136902	A_23_P322116	TFAP2E	Homo sapiens transcription factor AP-2 epsilon (activating	NM_178548	0.31236	2.05288	0.00821
2331879	A_33_P3274134	TMEM151B	Homo sapiens transmembrane protein 151B (TMEM151B), n	NM_001137560	0.3101	2.04222	4.60E-06
2336008	A_19_P00805168		lincRNA:chr16:88805249-88810399 forward strand		0.30948	2.03928	0.00115
882428	A_24_P314451	F8	Homo sapiens coagulation factor VIII, procoagulant compon	NM_000132	0.30946	2.03921	0.00362
1147167	A_23_P211196	C21orf67	Homo sapiens chromosome 21 open reading frame 67 (C21	NR_027129	0.30935	2.03866	0.00022
2313537	A_33_P3220853		PREDICTED: Homo sapiens hCG1645016 (LOC646249), mis	XR_041014	0.30808	2.03273	0.00973
2314068	A_33_P3454679		Unknown		0.30617	2.02379	9.70E-06
2320078	A_33_P3390217		tubulin tyrosine ligase-like family, member 9 [Source:HGNC	BC104025	0.30286	2.00845	0.00131
2321506	A_33_P3347787	LOC652022	PREDICTED: Homo sapiens e3 ubiquitin-protein ligase MIB	XM_941322	0.30266	2.00754	0.00366
2327713	A_33_P3305058		Homo sapiens cDNA FLJ43238 fis, clone HCHON2007881. [	AK125228	-0.3031	-2.00955	0.00253
2316603	A_19_P00324072		lincRNA:chr1:173968527-173991068 forward strand		-0.30476	-2.01724	0.00369
2317602	A_33_P3303309	LOC150622	Homo sapiens hypothetical LOC150622 (LOC150622), non-	NR_026832	-0.30687	-2.02709	0.00677
2334990	A_33_P3212244	KLRAP1	Homo sapiens killer cell lectin-like receptor subfamily A pse	NR_028045	-0.31121	-2.04743	0.00334
2316714	A_19_P00808928		lincRNA:chr2:74212792-74262017 forward strand		-0.3153	-2.06681	0.00007
1165423	A_24_P119201	MBD2	Homo sapiens methyl-CpG binding domain protein 2 (MBD2	NM_015832	-0.31984	-2.08855	0.0024
2325438	A_33_P3299634	OR1L6	olfactory receptor, family 1, subfamily L, member 6 [Source	AB529307	-0.32191	-2.09852	0.00243
2320227	A_19_P00330815		lincRNA:chr11:2016408-2017024 forward strand		-0.32444	-2.11078	0.00276
2333098	A_19_P00802503		lincRNA:chr9:21879775-21938825 reverse strand		-0.32456	-2.11137	0.00004
2315583	A_33_P3257993	RNF125	Homo sapiens ring finger protein 125 (RNF125), mRNA [NM	NM_017831	-0.32501	-2.11352	0.00581
2315617	A_19_P00323876		lincRNA:chr12:130608397-130621372 forward strand		-0.32676	-2.12205	0.00908
1145392	A_23_P355776		non-protein coding RNA 266B [Source:HGNC Symbol;Acc:3	XM_002342011	-0.32723	-2.12435	0.00339
2328665	A_33_P3226129		PREDICTED: Homo sapiens similar to ribosomal protein S7	XR_036913	-0.32759	-2.12612	0.00658
2329097	A_33_P3254320	SH2D3A	Homo sapiens SH2 domain containing 3A (SH2D3A), mRNA	NM_005490	-0.33216	-2.14861	0.00276
1163975	A_23_P57492	SLC5A4	Homo sapiens solute carrier family 5 (low affinity glucose co	NM_014227	-0.33705	-2.17294	0.00487
2312680	A_33_P3391105	LRP1	Homo sapiens low density lipoprotein receptor-related prot	NM_002332	-0.34238	-2.19978	0.00006
2328986	A_19_P00331229		lincRNA:chr8:17355272-17386123 forward strand		-0.34696	-2.22311	0.00002
2322777	A_19_P00812893		lincRNA:chr8:90615284-90628709 forward strand		-0.34798	-2.22834	0.00831
888980	A_23_P18078	RARRES1	Homo sapiens retinoic acid receptor responder (tazarotene	NM_002888	-0.34869	-2.23198	2.90E-07
2328064	A_33_P3338853	LOC401233	Homo sapiens HIV-1 Tat specific factor 1 pseudogene (LO	NR_033884	-0.3525	-2.25167	0.00549
1152502	A_23_P362759	PRDM5	Homo sapiens PR domain containing 5 (PRDM5), mRNA [N	NM_018699	-0.35416	-2.26025	0.00003
2329130	A_19_P00316238		lincRNA:chr1:26210671-26219098 reverse strand		-0.35856	-2.2833	0.00205
2336026	A_33_P3250055	MAPK12	Homo sapiens cDNA, FLJ98809. [AK308768]	AK308768	-0.36282	-2.3058	0.00005
2316073	A_33_P3244568	LOC100128675	Homo sapiens hypothetical LOC100128675 (LOC10012867	NR_024561	-0.36656	-2.32573	0.00885
883692	A_23_P62588	CALML6	Homo sapiens calmodulin-like 6 (CALML6), mRNA [NM_138	NM_138705	-0.37398	-2.36579	0.00213
2335735	A_33_P3287731	MYST4	Homo sapiens MYST histone acetyltransferase (monocytic	NM_012330	-0.37405	-2.36618	0.0008
2313814	A_19_P00321176		lincRNA:chrX:110799569-110812724 reverse strand		-0.37549	-2.37406	0.00339
2313020	A_33_P3410584	LOC645586	Homo sapiens cDNA FLJ25208 fis, clone REC05984. [AK05	AK057937	-0.37997	-2.39865	0.00034
2313824	A_33_P3651282	SNORA20	HHAGE017587 Human liver regeneration after partial hepat	DW416857	-0.38413	-2.42175	8.92E-08
2336113	A_19_P00802450		lincRNA:chr9:132236854-132272279 forward strand		-0.38468	-2.42482	0.00045
2313742	A_33_P3347152		suppressor of Ty 3 homolog (S. cerevisiae) [Source:HGNC Symbol;Acc:11466		-0.38601	-2.43225	0.00031
2335472	A_33_P3260066	BEAN1	Homo sapiens brain expressed, associated with NEDD4, 1 (	NM_001178020	-0.38682	-2.43677	0.00913
2313782	A_33_P3240773		Unknown	CR607250	-0.3884	-2.44567	0.00355
2332215	A_19_P00802011		lincRNA:chr9:4869500-4896050 reverse strand		-0.39417	-2.47841	4.23E-07
1150646	A_23_P41314	F11	Homo sapiens coagulation factor XI (F11), mRNA [NM_0001	NM_000128	-0.39485	-2.48226	0.00259
2319869	A_33_P3209326		Homo sapiens cDNA FLJ39676 fis, clone SMINT2009832. [	AK096995	-0.39715	-2.49544	0.0003
2317707	A_19_P00330956		lincRNA:chr2:43148721-43166396 reverse strand		-0.39988	-2.51118	0.00835
2333265	A_33_P3615334		Homo sapiens cDNA FLJ46134 fis, clone TESTI2051873. [A	AK128015	-0.40792	-2.55811	0.00437
2312886	A_33_P3331711	LOC100130463	Homo sapiens cDNA FLJ42307 fis, clone TRACH2005066. [	AK124300	-0.41029	-2.57214	1.60E-08
1139580	A_32_P129950	NHLRC3	Homo sapiens NHL repeat containing 3 (NHLRC3), transcri	NM_001012754	-0.41161	-2.57992	1.41E-08
2327085	A_33_P3300740		PM0-GN0344-190201-003-d05 GN0344 Homo sapiens cD	BI056255	-0.41819	-2.61931	0.00799
2315065	A_33_P3809328	PRO0471	Homo sapiens PRO0471 mRNA, complete cds. [AF111846]	AF111846	-0.42324	-2.64997	0.00258
2312878	A_19_P00810938		lincRNA:chr6:3176201-3194126 forward strand		-0.42796	-2.67893	0.00024
2324488	A_33_P3299122	HNF1A	H.sapiens HNF1-C mRNA. [X71347]	X71347	-0.4296	-2.68905	0.00654
2318015	A_33_P3323934		PREDICTED: Homo sapiens similar to JTV1 (LOC10012823	XR_038083	-0.45335	-2.84017	0.00308
886923	A_23_P32115	LCN12	Homo sapiens lipocalin 12 (LCN12), mRNA [NM_178536]	NM_178536	-0.45378	-2.84303	0.00223
2329975	A_33_P3277808		Unknown		-0.45898	-2.87726	2.06E-06
2319657	A_33_P3383079		Unknown		-0.46029	-2.88593	0.00668
1160842	A_23_P163697	SYT17	Homo sapiens synaptotagmin XVII (SYT17), mRNA [NM_016	NM_016524	-0.46121	-2.89206	3.20E-07
1145578	A_32_P223173		PREDICTED: Homo sapiens similar to acetyl-Coenzyme A	XR_037043	-0.46312	-2.90483	1.89E-09
887209	A_23_P256561	TLR6	Homo sapiens toll-like receptor 6 (TLR6), mRNA [NM_0060	NM_006068	-0.47231	-2.96692	5.39E-08
2320559	A_19_P00319186		lincRNA:chr2:39197219-39200415 forward strand		-0.47524	-2.98702	0.00003
2313354	A_19_P00328920		lincRNA:chr12:65991133-66071408 reverse strand		-0.48165	-3.03146	0.00002
2320097	A_33_P3383009		Unknown		-0.48357	-3.04487	0.00045
2314202	A_19_P00803322		lincRNA:chr11:88074377-88083852 forward strand		-0.49053	-3.09405	0.00733
2313592	A_19_P00321740		lincRNA:chr18:60251678-60254943 reverse strand		-0.49203	-3.10477	0.00685
2322438	A_33_P3307490		Homo sapiens cDNA FLJ45043 fis, clone BRAWH3021012. [	AK126988	-0.49367	-3.11656	0.00256
1140995	A_23_P254353	NOXA1	Homo sapiens NADPH oxidase activator 1 (NOXA1), mRNA	NM_006647	-0.49836	-3.15035	1.54E-10
2313552	A_33_P3321996		DB053889 TESTI2 Homo sapiens cDNA clone TESTI20445	DB053889	-0.50702	-3.21378	2.94E-07
2315265	A_33_P3260014	TPO	Homo sapiens thyroid peroxidase (TPO), transcript variant	NM_175721	-0.51881	-3.30226	0.00399

Supplementary Table 3. Continued.

2317948	A_19_P00316934		lincRNA:chr8:58055261-58132001 forward strand	-0.51913	-3.30468	0.00117
2333251	A_19_P00326790		lincRNA:chr7:106355964-106363089 reverse strand	-0.52782	-3.37149	1.51E-07
2324883	A_33_P3319860	IFFO1	Homo sapiens intermediate filament family orphan 1 (IFFO1; NR_036467	-0.52955	-3.38494	0.00227
1156659	A_23_P350005	TRIML2	Homo sapiens tripartite motif family-like 2 (TRIML2), mRNA NM_173553	-0.52969	-3.38603	0.00132
880437	A_23_P47728	MAP6	Homo sapiens microtubule-associated protein 6 (MAP6), tr NM_033063	-0.53627	-3.43774	1.43E-11
2333100	A_33_P3227482	FLJ43944	Homo sapiens cDNA FLJ43944 fis, clone TESTI4014392. [A AK125932	-0.54336	-3.49427	6.62E-06
2332940	A_33_P3339990	LOC100128429	Homo sapiens cDNA FLJ41329 fis, clone BRAMY2047676. [AK123323	-0.54752	-3.52796	3.45E-07
2328822	A_19_P00322673		lincRNA:chr7:26438220-26519642 forward strand	-0.54851	-3.53599	0.00589
1145139	A_24_P332816	RIMS2	Homo sapiens regulating synaptic membrane exocytosis 2 (I NM_014677	-0.55269	-3.57015	0.00712
1140073	A_23_P431505	XKR6	Homo sapiens primary neuroblastoma cDNA, clone:Nbla004; AB073660	-0.55294	-3.5722	1.18E-10
2325684	A_33_P3213792	KCNK4	Homo sapiens potassium channel, subfamily K, member 4 (K NM_033310	-0.55522	-3.59102	0.00045
1147079	A_23_P337849	CELF3	Homo sapiens CUGBP, Elav-like family member 3 (CELF3), NM_007185	-0.5579	-3.61324	9.54E-06
2322193	A_19_P00321950		lincRNA:chr2:148790300-148791401 reverse strand	-0.5714	-3.72736	0.00042
2319089	A_19_P00800085		lincRNA:chrX:73422428-73423502 forward strand	-0.57241	-3.73599	0.00842
1143034	A_23_P16694	CPS1-IT	Homo sapiens CPS1 intronic transcript (non-protein coding NR_002763	-0.5732	-3.74282	2.02E-06
2319233	A_33_P3249958	LOC642852	Homo sapiens hypothetical LOC642852 (LOC642852), non- NR_026943	-0.57607	-3.76764	0.00244
2335923	A_19_P00324364		lincRNA:chr9:33011575-33024766 reverse strand	-0.57631	-3.76975	0.00176
2318439	A_33_P3403595		Putative uncharacterized protein FLJ46792 [Source:UniPr AK128633	-0.57792	-3.78375	0.00374
2313741	A_19_P00327103		lincRNA:chr3:37886971-37901989 forward strand	-0.58031	-3.8046	1.33E-11
2325561	A_19_P00803876		lincRNA:chr2:235342486-235379011 forward strand	-0.58109	-3.81149	1.15E-12
2312797	A_19_P00804825		lincRNA:chr3:112308735-112319860 reverse strand	-0.58239	-3.82285	2.67E-10
2312796	A_19_P00320255		lincRNA:chr15:21165302-21166134 forward strand	-0.58298	-3.82806	0.00017
2312799	A_33_P3370265	LOC100508645	PREDICTED: Homo sapiens hypothetical protein LOC10050 XM_003120021	-0.58309	-3.82902	0.00014
2322529	A_33_P3368144	LOC652614	PREDICTED: Homo sapiens BOLA class I histocompatibility XM_942149	-0.58731	-3.8664	0.0064
882700	A_24_P181254	OLFM4	Homo sapiens olfactomedin 4 (OLFM4), mRNA [NM_006418; NM_006418	-0.58914	-3.88276	0.00587
1164351	A_24_P379353	CCDC24	Homo sapiens coiled-coil domain containing 24 (CCDC24), NM_152499	-0.60156	-3.99542	1.06E-11
1149190	A_24_P324712	NPC1L1	Homo sapiens NPC1 (Niemann-Pick disease, type C1, gene; NM_013389	-0.60347	-4.01305	0.00268
2334624	A_19_P00322995		lincRNA:chr3:156503913-156504373 reverse strand	-0.60767	-4.05198	0.00021
2317899	A_33_P3273767		Unknown	-0.61281	-4.10027	0.00809
2314399	A_33_P3275702	FMO2	Homo sapiens flavin containing monooxygenase 2 (non-func NM_001460	-0.61666	-4.1368	0.00049
1140861	A_23_P385126	DEPDC7	Homo sapiens DEP domain containing 7 (DEPDC7), transcri NM_139160	-0.6204	-4.17258	0.00163
2330315	A_19_P00321078		lincRNA:chr19:52588083-52597524 forward strand	-0.63852	-4.35029	8.36E-14
1138097	A_24_P928969	PTPN3	Homo sapiens protein tyrosine phosphatase, non-receptor t NM_001145369	-0.64097	-4.37493	0.00151
1160116	A_23_P164068	DNAH9	Homo sapiens dynein, axonemal, heavy chain 9 (DNAH9), tr NM_001372	-0.6442	-4.40762	0.00143
2317351	A_33_P3808516		Homo sapiens clone Z'3-1 placenta expressed mRNA from U66047	-0.64429	-4.40848	0.00243
1144094	A_23_P31798	NAT2	Homo sapiens N-acetyltransferase 2 (arylamine N-acetyltr NM_000015	-0.64784	-4.44462	9.20E-16
2334258	A_33_P3558740	LOC254100	Homo sapiens cDNA FLJ33054 fis, clone TRACH1000106. [AK057616	-0.65371	-4.50516	0.00203
2314489	A_19_P00800302		lincRNA:chr14:69118322-69174922 reverse strand	-0.65852	-4.55536	0.00875
1161185	A_23_P203267	TRIM29	Homo sapiens tripartite motif-containing 29 (TRIM29), mRN NM_012101	-0.66856	-4.66188	0.00042
1145452	A_23_P23646	USF1	Homo sapiens upstream transcription factor 1 (USF1), tran NM_007122	-0.6751	-4.73263	3.08E-17
2325366	A_33_P3395650	PCDHGA5	Homo sapiens protocadherin gamma subfamily A, 5 (PCDHC NM_032054	-0.67669	-4.75	0.00738
2314698	A_19_P00323109		lincRNA:chr12:97010269-97032694 forward strand	-0.67888	-4.77398	0.00239
1151276	A_24_P870620	PTN	Homo sapiens pleiotrophin (PTN), mRNA [NM_002825] NM_002825	-0.68525	-4.84449	0.00024
2314084	A_19_P00809175		lincRNA:chrX:11053954-11091179 reverse strand	-0.6854	-4.8462	0.00979
2316717	A_33_P3423984		palladin, cytoskeletal associated protein [Source:HGNC Syn AK096458	-0.6909	-4.90792	0.00965
2313090	A_19_P00320641		lincRNA:chr16:72557245-72661585 reverse strand	-0.69126	-4.91208	1.19E-14
880634	A_23_P254768	HCG4	Homo sapiens HLA complex group 4 (HCG4), non-coding RI NR_002139	-0.69362	-4.93881	0.00876
2335810	A_19_P00805748		lincRNA:chr16:58783541-59142878 forward strand	-0.69364	-4.93904	0.00791
1162739	A_24_P84657	MUC2	Homo sapiens mucin 2, oligomeric mucus/gel-forming (MUC NM_002457	-0.69462	-4.95013	0.00776
2323266	A_33_P3219537	NCRNA00207	Homo sapiens non-protein coding RNA 207 (NCRNA00207), NR_028409	-0.69783	-4.98685	0.00678
2325771	A_33_P3570193	FLJ36000	Homo sapiens hypothetical FLJ36000 (FLJ36000), non-codi NR_027084	-0.69924	-5.00311	0.00523
2327782	A_19_P00329113		lincRNA:chr7:80367-94117 forward strand	-0.70778	-5.10252	4.47E-15
2319943	A_19_P00331241		lincRNA:chr13:24717800-24728575 forward strand	-0.70905	-5.11744	0.00566
2312639	A_19_P00317957		lincRNA:chr19:11759250-11763258 reverse strand	-0.7129	-5.16296	0.00523
2313551	A_33_P3280471	ERBB3	Homo sapiens v-erb-b2 erythroblastic leukemia viral oncog NM_001982	-0.71402	-5.17628	0.00431
2315288	A_19_P00802238		lincRNA:chr10:103459185-103464735 reverse strand	-0.71792	-5.22298	0.00533
2313500	A_33_P3397507		adaptor-related protein complex 1, sigma 2 subunit [Source AK299921	-0.71914	-5.23772	0.00403
2312560	A_33_P3228985	LOC100132396	Homo sapiens zinc finger protein 705D-like (LOC10013239E NM_001193630	-0.7266	-5.32838	0.00372
2325597	A_33_P3314352		Unknown	-0.72743	-5.33869	0.00325
885381	A_23_P76322	PIK3C2G	Homo sapiens phosphoinositide-3-kinase, class 2, gamma p NM_004570	-0.73251	-5.40147	0.00007
2323131	A_33_P3213617		V_segment translation product Fragment [Source:UniProtK A25493	-0.7368	-5.45504	2.53E-07
2336019	A_33_P3465703	SNORA60	601888042F1 NIH_MGC_17 Homo sapiens cDNA clone IMAC BF304636	-0.7399	-5.49411	1.84E-14
2313028	A_33_P3354688		full-length cDNA clone CS0DB006YB20 of Neuroblastoma (CR625008	-0.74303	-5.5339	1.60E-15
2325770	A_33_P3327642	AIM1L	Homo sapiens absent in melanoma 1-like (AIM1L), mRNA [N NM_001039775	-0.74437	-5.551	0.0001
2312771	A_33_P3416757	PRLR	Homo sapiens prolactin receptor (PRLR), mRNA [NM_00094 NM_000949	-0.74835	-5.60213	0.00223
2316883	A_33_P3209772	POU4F1	Homo sapiens POU class 4 homeobox 1 (POU4F1), mRNA [NM_006237	-0.74869	-5.60644	0.00217
2312879	A_19_P00318216		lincRNA:chr8:129022630-129113453 reverse strand	-0.75216	-5.65144	0.00203
2330096	A_33_P3305710		Unknown	-0.75729	-5.71858	0.00378
880995	A_24_P216014	GH2	Homo sapiens growth hormone 2 (GH2), transcript variant 3 NM_022558	-0.7612	-5.77039	0.00002
2333946	A_33_P3251801	KLRG2	Homo sapiens killer cell lectin-like receptor subfamily G, me NM_198508	-0.76298	-5.79407	0.00869
1138131	A_32_P88415	MYOZ3	Homo sapiens myozenin 3 (MYOZ3), transcript variant 2, ml NM_133371	-0.76392	-5.80655	5.28E-16

Supplementary Table 3. Continued.

2325567	A_19_P00322088		lincRNA:chr16:11477618-11478868 reverse strand	-0.76513	-5.82277	1.77E-11
2333164	A_33_P3209135		Unknown	-0.76689	-5.84641	7.85E-06
2332688	A_19_P00321586		lincRNA:chrX:97828872-98120013 forward strand	-0.77403	-5.9433	0.00111
2313011	A_19_P00804574		lincRNA:chr19:56789963-56824563 forward strand	-0.77426	-5.94646	0.00116
2313318	A_33_P3234482	LOC100132919	PREDICTED: Homo sapiens hypothetical protein LOC10013 XM_001715268	-0.77534	-5.96124	0.00091
889093	A_23_P167585	GDF9	Homo sapiens growth differentiation factor 9 (GDF9), mRNA NM_005260	-0.77938	-6.01704	3.89E-07
2329974	A_33_P3346691		Unknown	-0.7879	-6.13625	0.0006
2316558	A_19_P00319331		lincRNA:chrX:53707036-53713697 reverse strand	-0.78942	-6.15776	0.00087
2326173	A_33_P3362063	LOC440925	Homo sapiens hypothetical LOC440925 (LOC440925), non- NR_027433	-0.79096	-6.17965	0.00071
2315136	A_33_P3381613		Unknown	-0.7941	-6.22444	0.00001
2333453	A_19_P00805888		lincRNA:chr6:28134146-28139947 forward strand	-0.79613	-6.25365	0.00037
886684	A_23_P360924	CLDN17	Homo sapiens claudin 17 (CLDN17), mRNA [NM_012131] NM_012131	-0.80314	-6.3554	0.0003
2326094	A_33_P3383189	SP9	Homo sapiens Sp9 transcription factor homolog (mouse) (SI NM_001145250	-0.8115	-6.4789	7.98E-17
1144506	A_23_P42897	MGAM	Homo sapiens maltase-glucoamylase (alpha-glucosidase) (M NM_004668	-0.82548	-6.69084	0.00019
2312654	A_33_P3211968	LOC100133207	PREDICTED: Homo sapiens hypothetical LOC100133207 (L XR_110242	-0.83235	-6.79749	0.00019
2312824	A_33_P3418571		Unknown	-0.83401	-6.82358	5.20E-07
1141675	A_32_P78101	IGSF21	Homo sapiens immunoglobin superfamily, member 21 (IGSF NM_032880	-0.84127	-6.9386	5.50E-10
2312798	A_33_P3334000		Unknown	-0.84694	-7.02983	0.00012
2333099	A_33_P3872115	LOC727710	Homo sapiens cDNA FLJ25723 fis, clone TST05459. [AK09 AK098589	-0.86366	-7.30571	0.00002
888487	A_23_P3379	RASGRF1	Homo sapiens Ras protein-specific guanine nucleotide-rele NM_002891	-0.86497	-7.32771	0.00006
887149	A_23_P381172	MRAP	Homo sapiens melanocortin 2 receptor accessory protein (N NM_206898	-0.89367	-7.82837	1.99E-08
2332836	A_33_P3369016		PREDICTED: Homo sapiens hypothetical LOC645685 (LOC XM_928694	-0.8938	-7.83071	2.56E-08
2312922	A_33_P3232273		Unknown	-0.91591	-8.23959	8.51E-06
2333317	A_19_P00320499		lincRNA:chr6:132224787-132228642 forward strand	-0.91961	-8.31015	1.22E-13
2333382	A_33_P3875570	LOC100270679	UI-1-BC1p-akz-b-02-0-UI.s1 NCI_CGAP_PI3 Homo sapien BQ013595	-0.92175	-8.35121	3.17E-06
886971	A_23_P97402	CAMK1G	Homo sapiens calcium/calmodulin-dependent protein kinase NM_020439	-0.93375	-8.58523	6.51E-06
2330192	A_19_P00328485		lincRNA:chr5:124817426-124830024 reverse strand	-0.93549	-8.61957	3.74E-12
2332403	A_19_P00804120		lincRNA:chr13:41416375-41460125 forward strand	-0.93602	-8.63013	1.96E-14
1163074	A_32_P84373	FAM153C	Homo sapiens family with sequence similarity 153, member NM_001079527	-0.9466	-8.8431	2.15E-06
2313088	A_33_P3341007		cDNA FLJ40133 fis, clone TESTI2012231 [Source:UniProt XR_114873	-0.96792	-9.28803	1.08E-07
2312663	A_33_P3250899		Unknown	-0.98173	-9.58814	4.04E-07
1143061	A_23_P91317	RAD21L1	Homo sapiens RAD21-like 1 (S. pombe) (RAD21L1), mRNA NM_001136566	-0.98296	-9.6153	1.50E-06
1142161	A_32_P23308	LOC257358	Homo sapiens hypothetical LOC257358 (LOC257358), non- NR_026945	-0.98956	-9.76243	3.34E-07
2312753	A_33_P3264657	CYP27C1	Homo sapiens cytochrome P450, family 27, subfamily C, pol NM_001001665	-0.99717	-9.93507	4.14E-15
1166646	A_23_P38696	DSC1	Homo sapiens desmocollin 1 (DSC1), transcript variant Dsc NM_004948	-0.99822	-9.95912	2.75E-13
2326138	A_33_P3361067	ABCG2	Homo sapiens ATP-binding cassette, sub-family G (WHITE) NM_004827	-0.99899	-9.97685	1.66E-20
2312599	A_19_P00800410		lincRNA:chr18:68000270-68008995 reverse strand	-1.00738	-10.17138	1.18E-07
879364	A_23_P155057	CYTH4	Homo sapiens cytohesin 4 (CYTH4), mRNA [NM_013385] NM_013385	-1.01406	-10.32905	7.73E-19
881712	A_24_P267592	SAMHD1	Homo sapiens SAM domain and HD domain 1 (SAMHD1), ml NM_015474	-1.03336	-10.79836	2.68E-19
888760	A_23_P134139	FABP7	Homo sapiens fatty acid binding protein 7, brain (FABP7), m NM_001446	-1.03401	-10.8147	7.36E-12
2327866	A_33_P3218316	VSTM2B	Homo sapiens V-set and transmembrane domain containing NM_001146339	-1.04503	-11.0926	1.15E-08
2328342	A_19_P00807753		lincRNA:chr5:67086094-67113044 reverse strand	-1.04571	-11.10989	1.18E-17
1140900	A_23_P259049	SLC45A2	Homo sapiens solute carrier family 45, member 2 (SLC45A2 NM_016180	-1.11511	-13.03499	7.12E-20
2330070	A_33_P3409458		Uncharacterized protein C9orf171 [Source:UniProtKB/Swiss-Prot;Acc:Q6ZC	-1.14373	-13.923	1.86E-11
2334800	A_33_P3723448	ART3	Homo sapiens ADP-ribosyltransferase 3 (ART3), transcript NM_001130017	-1.17836	-15.07864	8.45E-16
880981	A_24_P25544	GDNF	Homo sapiens glial cell derived neurotrophic factor (GDNF), NM_000514	-1.20466	-16.01995	4.49E-16
2312865	A_33_P3247589		Unknown	-1.21262	-16.31635	1.20E-12
1139286	A_32_P202182	SPDYE8P	Homo sapiens speedy homolog E8 (Xenopus laevis), pseudo NR_003664	-1.23975	-17.36789	2.10E-20
1160858	A_23_P307570	ABCC13	Homo sapiens ATP-binding cassette, sub-family C (CFTR/I NR_003087	-1.24306	-17.50096	1.34E-13
2327680	A_19_P00321448		lincRNA:chr7:19970681-20008020 forward strand	-1.24691	-17.65662	1.11E-13
2313089	A_33_P3311543		Unknown	-1.26456	-18.38887	5.28E-20
2336005	A_33_P3227736	C6orf227	Homo sapiens chromosome 6 open reading frame 227 (C6o NR_027908	-1.27418	-18.80104	2.76E-13
1162291	A_23_P215331	CRHR2	Homo sapiens corticotropin releasing hormone receptor 2 (NM_001883	-1.28339	-19.20403	5.49E-22
1140976	A_24_P30557	TBX5	Homo sapiens T-box 5 (TBX5), transcript variant 1, mRNA NM_000192	-1.29405	-19.68121	4.95E-18
2332835	A_19_P00324742		lincRNA:chr5:54162493-54208143 forward strand	-1.30987	-20.4114	6.82E-21
2332602	A_33_P3404257	VN1R103P	Homo sapiens cDNA FLJ43558 fis, clone PROST2018607. [AK125546	-1.33867	-21.81086	1.83E-20
2313136	A_19_P00321802		lincRNA:chr6:64472442-64498999 reverse strand	-1.33898	-21.82626	1.14E-15
2333454	A_19_P00329766		lincRNA:chrX:115401722-115413847 forward strand	-1.34922	-22.34698	2.43E-21
2332480	A_33_P3265254		Unknown	-1.35574	-22.68483	4.34E-16
1141555	A_23_P385199	EPHA10	Homo sapiens EPH receptor A10 (EPHA10), transcript vari NM_173641	-1.39648	-24.91625	1.09E-21
1147323	A_23_P316472	DNHD1	Homo sapiens dynein heavy chain domain 1 (DNHD1), trans NM_144666	-1.39871	-25.0442	1.05E-21
2316774	A_19_P00807075		lincRNA:chr1:224624052-224647602 reverse strand	-1.40834	-25.60564	3.77E-17
2325165	A_33_P3358943	GRM2	Homo sapiens glutamate receptor, metabotropic 2 (GRM2), NM_000839	-1.40849	-25.61499	2.15E-21
882326	A_23_P29608	IQCF5	Homo sapiens IQ motif containing F5 (IQCF5), mRNA [NM_C NM_001145059	-1.42745	-26.75789	2.40E-17
2333525	A_19_P00319066		lincRNA:chr8:122966846-123139423 reverse strand	-1.44404	-27.79938	1.49E-21
2330100	A_33_P3507270	EFCAB5	Homo sapiens EF-hand calcium binding domain 5 (EFCAB5) NM_198529	-1.51262	-32.55487	4.26E-19
879191	A_23_P347610	HAVCR1	Homo sapiens hepatitis A virus cellular receptor 1 (HAVCR NM_012206	-1.52206	-33.27081	5.65E-21
2317054	A_19_P00813078		lincRNA:chr4:74537611-74598511 forward strand	-1.52792	-33.72248	5.66E-22
2332859	A_33_P3294277	CYP4F3	Homo sapiens cytochrome P450, family 4, subfamily F, poly NM_000896	-1.52822	-33.74596	3.07E-21
1160252	A_24_P169507	MUC17	Homo sapiens mucin 17, cell surface associated (MUC17), n NM_001040105	-1.53063	-33.93393	2.75E-19
882334	A_24_P203000	IL2RB	Homo sapiens interleukin 2 receptor, beta (IL2RB), mRNA [I NM_000878	-1.53412	-34.20722	3.41E-21

Supplementary Table 3. Continued.

2312901	A_19_P00323817		lincRNA:chr11:129133890-129145940 forward strand	-1.55651	-36.01689	1.39E-19
2332860	A_19_P00804713		lincRNA:chrX:39980256-39991281 forward strand	-1.56545	-36.76616	1.01E-19
869222	A_24_P390495	CX3CL1	Homo sapiens chemokine (C-X3-C motif) ligand 1 (CX3CL1 NM_002996	-1.61899	-41.5901	7.65E-23
1161067	A_23_P148641	TTTY12	Homo sapiens testis-specific transcript, Y-linked 12 (non-p NR_001551	-1.62203	-41.88262	2.27E-20
2316927	A_33_P3221578		PREDICTED: Homo sapiens FLJ45872 protein (FLJ45872), XR_114214	-1.62972	-42.63082	2.23E-20
2313034	A_33_P3303982	UBTFL1	Homo sapiens upstream binding transcription factor, RNA p NM_001143975	-1.64645	-44.30507	1.24E-20
2332858	A_33_P3261695	LOC647310	Homo sapiens testis expressed gene 22 (LOC647310), mRN NM_001195082	-1.65824	-45.52351	1.93E-22
2317053	A_33_P3236122		Unknown	-1.66349	-46.07733	1.00E-20
1137544	A_23_P425681	CCK	Homo sapiens cholecystokinin (CCK), transcript variant 1, n NM_000729	-1.68606	-48.53609	1.93E-22
2313823	A_33_P3754922	RNU11	BP873537 Sugano cDNA library, embryonal kidney Homo sa BP873537	-1.70751	-50.99257	1.41E-22
886683	A_32_P54274	DRD5	Homo sapiens dopamine receptor D5 (DRD5), mRNA [NM_0(NM_000798	-1.7391	-54.83998	7.07E-23
881201	A_24_P128713		ankyrin repeat domain 44 [Source:HGNC Symbol;Acc:25259 AK097086	-1.74131	-55.12009	2.14E-21
2313161	A_33_P3331150		Human IG rearranged H-chain mRNA V-region, partial cds. L03830	-1.74812	-55.9916	1.71E-21
885170	A_23_P355394	STK32A	Homo sapiens serine/threonine kinase 32A (STK32A), trans NM_145001	-1.75271	-56.58576	7.69E-22
2328230	A_33_P3348395		Uncharacterized protein ENSP00000372125 [Source:UniProtKB/Swiss-Prot;	-1.75821	-57.30698	1.09E-21
2325685	A_33_P3214670	HK2	Homo sapiens hexokinase 2 (HK2), mRNA [NM_000189]. NM_000189	-1.90182	-79.76683	2.24E-22
1155509	A_24_P492083		AF150244 Human mRNA from cd34+ stem cells Homo sapie AF150244	-1.96759	-92.80843	1.36E-22
1145348	A_23_P82099	NOX3	Homo sapiens NADPH oxidase 3 (NOX3), mRNA [NM_01571 NM_015718	-2	-100	2.84E-23
1139895	A_23_P304304	ARSF	Homo sapiens arylsulfatase F (ARSF), mRNA [NM_004042] NM_004042	-2	-100	5.05E-23
2313224	A_33_P3403270		coiled-coil domain containing 33 [Source:HGNC Symbol;Acc CN265765	-2	-100	6.09E-23
2313619	A_19_P00805987		lincRNA:chr18:53398227-53474402 reverse strand	-2	-100	8.93E-23
2332992	A_33_P3276386		Unknown	-2	-100	5.69E-23

Supplementary Table 4. List of genes expressed greater than 2.0 folds in *S. mutans* TW295 infection.

		Fold increase		
Gene	Accession number	TW295 vs TW295CND	TW295 vs no infection	TW295CND vs no infection
ADHFE1	NM_144650	19.07	10.99	-
ANKMY1	NM_017844	2.78	2.30	-
ARHGAP9	NM_032496	8.51	3.35	-
ARHGEF38	NM_017700	2.90	3.47	-
ARNT2	NM_14862	3.04	2.96	-
ATP8B3	NM_138813	3.95	3.67	-
AURKC	NM_001015878	3.07	2.63	-
AWAT2	NM_001002254	2.44	3.96	-
BMP10	NM_014482	6.14	12.67	-
C1orf14	NM_0.0933	7.34	3.56	-
C21orf54	NR_024102	100.00	100.00	-
C22orf43	NM_016449	3.06	2.56	-
C4orf40	NM_214711	15.18	15.25	-
C8orf41	NM_025115	2.04	2.15	-
CAND1	NM_018448	3.02	3.09	-
CBLN4	NM_080617	3.16	3.55	-
CRMP1	NM_001014809	2.16	2.59	-
DEFB103A	NM_001081551	5.46	5.60	-
DOC2A	NM_003586	7.32	6.40	-
EML5	NM_183387	3.88	3.22	-
ESX1	NM_153448	6.63	7.36	-
FAM178B	NM_001122646	4.29	6.46	-
FBXW12	NM_207102	3.23	2.07	-2.33
FLJ21408	AK025061	4.29	11.44	-
FLJ25363	NM_001145553	2.01	2.36	-
FLJ37505	NR_033987	15.09	10.78	-
FOS	NM_005252	6.96	5.62	-

Supplementary Table 4. Continued.

Gene	Accession number	Fold increase		
		TW295 vs TW295CND	TW295 vs no infection	TW295CND vs no infection
GPR179	NM_01004334	2.01	3.44	-
HAMP	NM_021175	3.03	2.77	-
IL18RAP	NM_003853	2.61	2.36	-
KCNAB2	NM_003636	4.71	2.33	-
KIAA1239	NM_001144990	8.83	2.66	-
KIAA1804	NM_032435	2.46	2.30	-
LCE1E	NM_178353	2.13	2.15	-
LLGL1	NM_004140	3.94	3.85	-
LOC100128496	CR738909	4.72	5.00	-
LOC100128942	CD049182	39.65	39.40	-
LOC100129931	NR_033828	2.65	2.24	-
LOC100130148	NR_024560	9.19	9.08	-
LOC100130857	AK097050	11.36	2.33	-5.09
LOC100131355	AK124217	2.08	2.09	-
LOC100131479	XM_001715121	2.83	2.26	-
LOC100132815	AK056856	2.50	2.14	-
LOC284276	NR_015417	5.41	4.32	-
LOC284440	NR_026956	2.07	2.10	-
LOC729770	XR_112442	3.17	3.38	-
LRRC2	NM_024512	3.07	2.44	-
LY6G6E	NR_024541	3.07	6.04	-
MFAP3L	NM_021647	6.37	6.17	-
MPL	NM_005373	4.73	4.66	-
NDOR1	NM_001144026	3.62	3.59	-
NMUR1	NM_006056	3.54	4.72	-
PCMTD1	NM_052937	2.75	2.28	-
PCNX	NM_014982	2.90	2.88	-
PEX5L	NM_016559	100.00	100.00	-

Supplementary Table 4. Continued.

Gene	Accession number	Fold increase		
		TW295 vs TW295CND	TW295 vs no infection	TW295CND vs no infection
PFKFB2	NM_006212	2.59	2.74	-
POFUT1	NM_015352	2.26	2.12	-
POU1F1	NM_000306	71.73	2.36	-
PTK2B	NM_173174	2.14	2.33	-
QPRT	NM_014298	2.25	2.66	-
RESP18	NM_001007089	8.68	2.30	-
RREB1	NM_001003700	2.09	2.15	-
SCARNA9L	NR_023358	2.33	3.85	-
SCNN1D	NM_001130413	5.21	5.00	-
SHOX	NM_006883	3.53	39.40	-
SLC10A7	NM_032128	3.13	2.24	-
SLC16A7	NM_004731	3.00	9.08	-
SOX 18	NM_018419	4.06	2.33	-
SOX30	NM_178424	2.70	2.09	-
SPDYE3	NM_001004351	3.05	2.26	-
TDGF1	NM_003212	2.59	2.14	-
TMED7-T1CAM2	NM_001164469	2.22	4.32	-
TMEM201	NM_001130924	2.49	2.10	-
TRH	NM_007117	3.09	3.38	-
TTTY18	NR_001550	8.75	2.44	-
VAX2	NM_012476	2.76	6.04	-
VGLL3	NM_016206	2.58	6.17	-
WFDC5	NM_145652	4.23	4.66	-
ZNF208	NM_007153	3.74	3.59	-
ZNF676	NM_001001411	2.20	4.72	-
ZNF737	NM_001159293	2.07	2.28	-
ZNF880	NM_001145434	2.22	2.88	-

Supplementary Table 5. Primers used in the RT-PCR assays.

Gene	Primer	Sequence	Size (bp)
ARHGEF38	ARHGEF38-F	TGT CAT TGT TGG AAG AGG CC	240
	ARHGEF38-R	TGA GAG GAG ATG GGA ATC AT	
ARHGAP9	ARHGAP9-F	TGG ACC AAC CCT GTT TCG GC	240
	ARHGAP9-F	AAG TCA CAC TCA TGA AGA TA	
GPR179	GPR179-F	CTG ACC CGT TCT CGG AGC ACC TAC AGA GAG	300
	GPR179-R	CTC AGC TTT CCG TTC CCC TAG ACC CTG GGT	
GAPDH	GAPDH-F	GGA GTC AAC GGA TTT GGT CGT ATT G	180
	GAPDH-R	CTT CCC GTT CTC AGC CTT GAC GGT G	

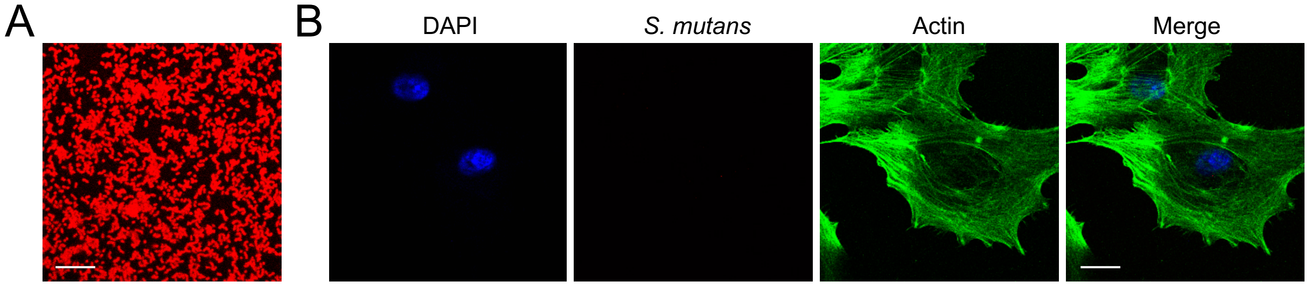

Supplementary Figure 1 Representative confocal laser scanning microscopic images of *S. mutans* MT8148 (CBP-negative) and MT8148 infecting HUVECs. (A) Representative confocal laser scanning microscopic images of MT8148. Bacteria cells are stained red (anti-*S. mutans*-Alexa Fluor 555). Scale bar, 10 µm. (B) Representative confocal laser scanning microscopic images of MT8148 infecting HUVECs. Nuclei are stained blue (DAPI), bacteria cells are stained red (anti-*S. mutans*-Alexa Fluor 555), and actin filaments are stained green (phalloidin-Alexa Fluor 448). Scale bar, 10 µm. All confocal laser scanning microscope images were taken using LSM510.

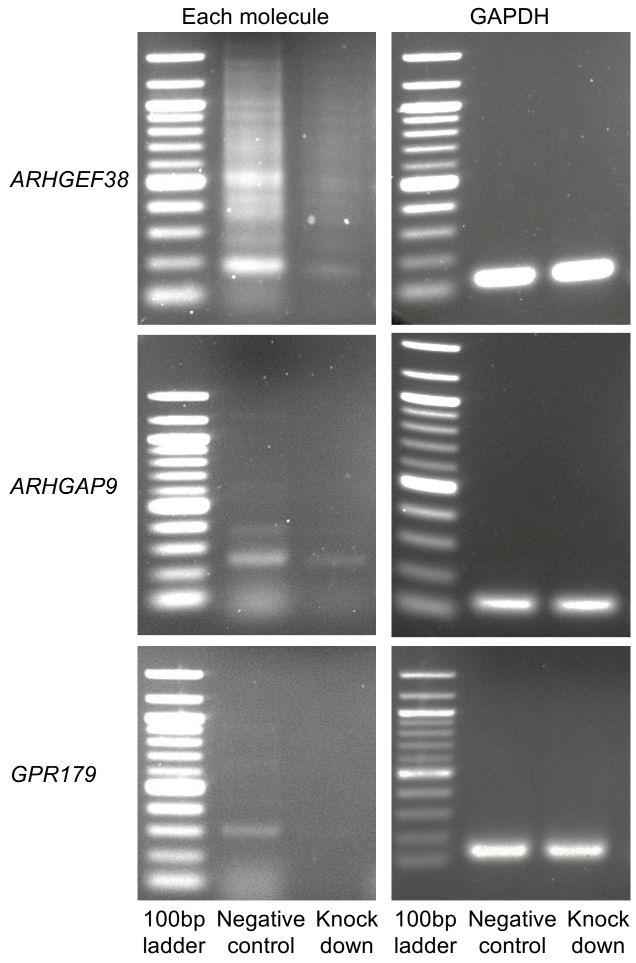

Supplementary Figure 2 RT-PCR analysis of HUVECs transfected with siRNAs targeting *ARHGAP9*, *ARHGEF38*, and *GPR179*. GAPDH mRNA expression was analyzed as an internal control.

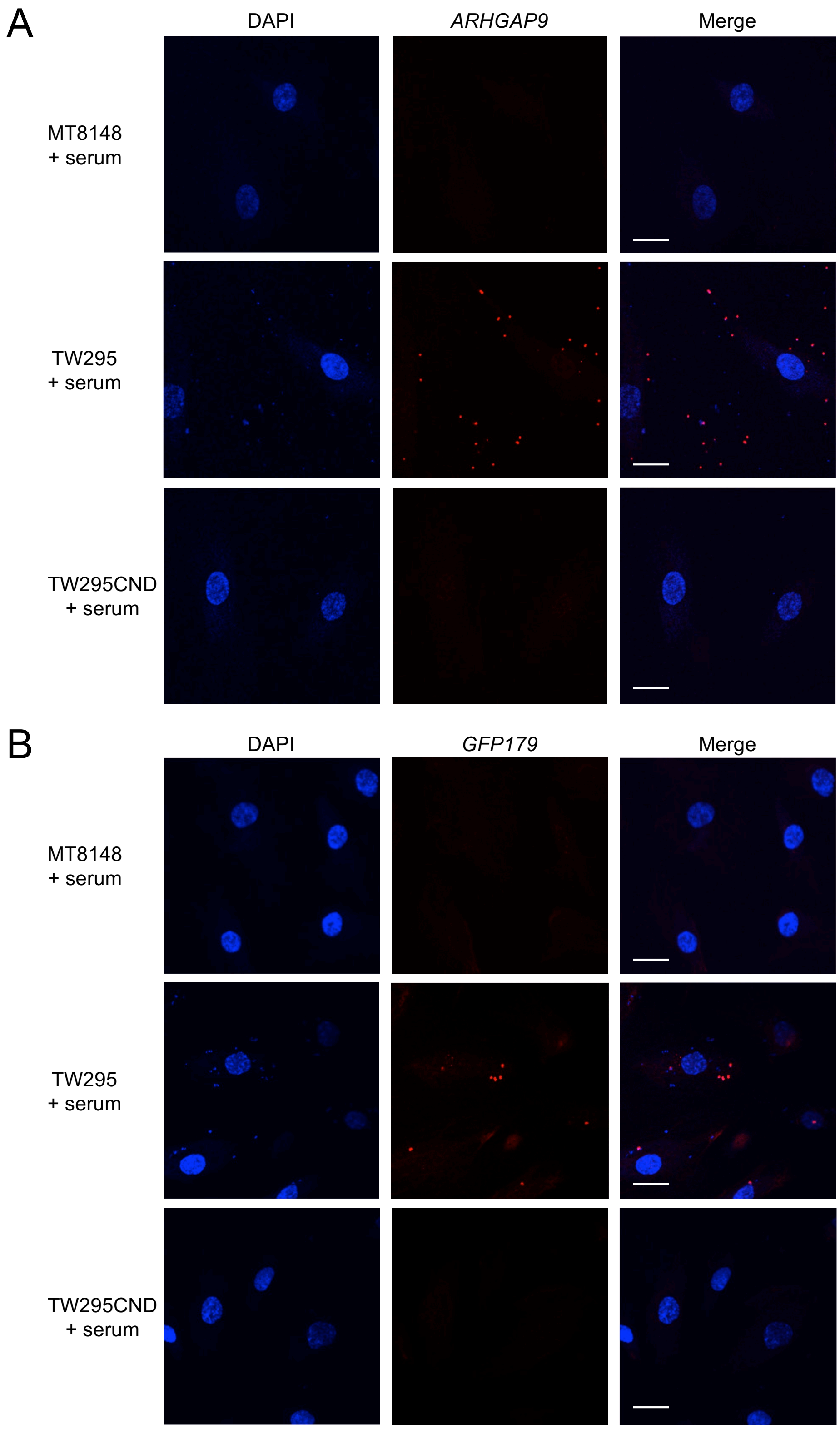

Supplementary Figure 3 Representative confocal laser scanning microscopic images of *S. mutans* invading HUVECs. Nuclei are stained blue; *ARHGAP9* (A) and *GFP179* (B) are stained red. Bar; 10 μm. All confocal laser scanning microscope images were taken using LSM510.

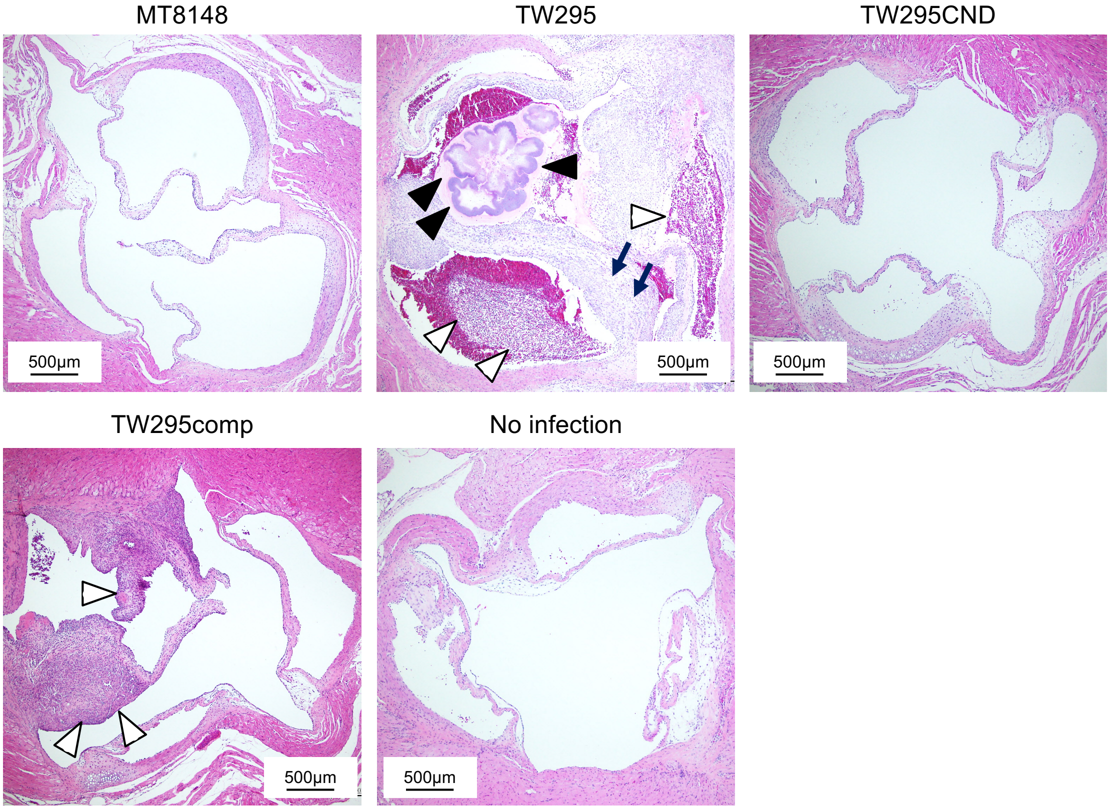

Supplementary Figure 4 Representative histopathological images following hematoxylin-eosin staining of tissue sections of extirpated hearts from rats infected with *S. mutans*. White arrowheads indicate infiltration of inflammatory cells and black arrowheads indicate fibrin-like deposition. Black arrows indicate hypertrophy of the endocardium.
